# Sizing, stabilising, and cloning repeat-expansions for gene targeting constructs

**DOI:** 10.1016/j.ymeth.2020.07.007

**Published:** 2021-07

**Authors:** Remya R. Nair, Charlotte Tibbit, David Thompson, Ross McLeod, Asif Nakhuda, Michelle M. Simon, Robert H. Baloh, Elizabeth M.C. Fisher, Adrian M. Isaacs, Thomas J. Cunningham

**Affiliations:** a Mammalian Genetics Unit, MRC Harwell Institute, Oxfordshire OX11 0RD, UK; b Board of Governors Regenerative Medicine Institute, Los Angeles, CA, USA; c Department of Neurology, Cedars-Sinai Medical Center, Los Angeles, CA, USA; d Department of Neuromuscular Diseases, UCL Institute of Neurology, London WC1N 3BG, UK; e Department of Neurodegenerative Disease, UCL Institute of Neurology, London WC1N 3BG, UK; f UK Dementia Research Institute at UCL, UCL Institute of Neurology, London WC1N 3BG, UK

## Abstract

Aberrant microsatellite repeat-expansions at specific loci within the human genome cause several distinct, heritable, and predominantly neurological, disorders. Creating models for these diseases poses a challenge, due to the instability of such repeats in bacterial vectors, especially with large repeat expansions. Designing constructs for more precise genome engineering projects, such as engineering knock-in mice, proves a greater challenge still, since these unstable repeats require numerous cloning steps in order to introduce homology arms or selection cassettes. Here, we report our efforts to clone a large hexanucleotide repeat in the *C9orf72* gene, originating from within a BAC construct, derived from a *C9orf72*-ALS patient. We provide detailed methods for efficient repeat sizing and growth conditions in bacteria to facilitate repeat retention during growth and sub-culturing. We report that sub-cloning into a linear vector dramatically improves stability, but is dependent on the relative orientation of DNA replication through the repeat, consistent with previous studies. We envisage the findings presented here provide a relatively straightforward route to maintaining large-range microsatellite repeat-expansions, for efficient cloning into vectors.

## Highlights

• Large GGGGCC repeat expansions within BAC vectors are highly unstable.
• CRISPR-Cas9 screening of BAC vector clones to determine repeat length.
• CRISPR-Cas9 cloning of GGGGCC repeat expansion regions into the linear pJazz vector.
• pJazz dramatically stabilizes GGGGCC repeat expansions over 4 kb in length.

## Graphical abstract

**Figure:**
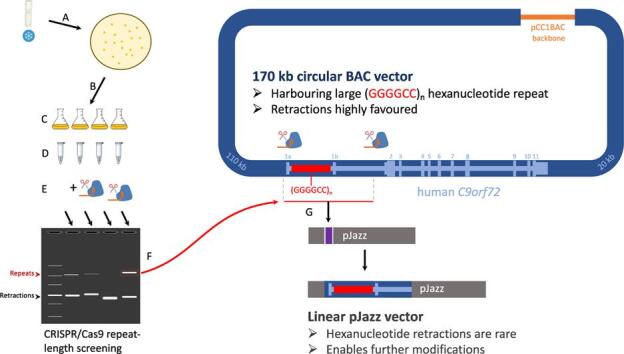

## 1 Introduction

Tracts of repetitive DNA are widespread throughout the human genome and are often highly polymorphic due to relative instability compared to non-repetitive DNA [1]. Repeats that fall within the proximity of genes can cause disease, frequently neurological in nature, due to local gene dysregulation or due to the production of toxic repeat containing RNA or protein molecules [2]. However, in many instances, disease mechanisms are poorly understood and cures remain elusive. Thus, accurate disease models are needed, both *in vitro* and *in vivo*, to facilitate better understanding and to develop new therapeutics. For some diseases, knock-in mouse models of repeat expansion disorders have been achieved, including mice that successfully model the polyglutamine expansion disorders spinal and bulbar muscular atrophy and Huntington disease [3], [4]. In each case, creation of these mouse models involved cloning relatively manageable stretches of CAG triplet repeats (<450 bp) into vectors for gene targeting. However, cloning repeat expansions for much larger repeats remains a major challenge. Engineering and manipulating large repeat sequences, maintaining stability of large repeat sequences in vectors for growth in bacteria, and characterising the size of large repeat sequences – especially in large vectors – are all major hurdles to overcome. At the *C9orf72* locus, a hexanucleotide repeat expansion with 100% GC content (GGGGCC) can expand into the hundreds, and even thousands, of repeats, and is the most common heritable cause of both amyotrophic lateral sclerosis (ALS) and frontotemporal dementia (FTD) [5], [6], [7]. *In vitro* models of *C9orf72* expansion typically constitute overexpression of relatively short repeat expansions, with the exception of patient-derived induced pluripotent stem cells. Similarly, existing *in vivo* models, including fly, zebrafish, and mouse, are achieved through overexpression and most harbour short repeat lengths [8]. Specifically in mouse, repeats have been introduced via AAV virus delivery or via traditional transgenesis using bacterial artificial chromosomes (BACs) derived from patient genomic DNA randomly integrated into the mouse genome [8].

Four different *C9orf72*-repeat-BAC transgenic mouse lines have been published, harbouring up to a maximum of 1000 repeats, although repeat length heterogeneity was reported in each study, and phenotypes are highly variable [9], [10], [11], [12]. All strains reported the presence of repeat-associated RNA foci and dipeptide repeat proteins linked to disease, although none exhibit *C9orf72* downregulation seen in patients, due to the nature of the model. Only one model, harbouring 500 repeats, was reported to exhibit motor phenotypes, together with classic TDP43 pathology observed in patients [11]. These differences between strains are hard to reconcile, but may be due to factors such as the different genetic backgrounds used, and genomic insertion site (which is random in each case), which likely influences transgene expression levels and repeat stability. Towards our goal to engineer a more physiological mouse model of *C9orf72* repeat expansion, we sought to find methods to stabilise *C9orf72* hexanucleotide repeat containing DNA sequences in bacterial culture, in order to facilitate further cloning to engineer a targeting construct for knock-in to the mouse *C9orf72* locus. Here, we report methods we have developed to accurately size, and ultimately stabilise, a long GGGGCC repeat derived from a BAC vector, which can be applied to other highly repetitive and unstable DNA sequences.

## 2 Materials and methods

### 2.1 Optimal conditions for growth and isolation of *C9orf72* repeat carrying BAC vector

Starting from a frozen glycerol stock of repeat-BAC harbouring DH10b *E. coli* bacteria, we streaked-out some of the frozen prep (using a sterile pipette tip) onto LB-agar plates. After 24 h incubation at 30 °C, colonies were picked into 5 ml LB liquid starter culture and incubated for 6 h at 30 °C with 200 rpm shaking; the starter culture was then seeded into a 500 ml culture for 16 h, 200 rpm at 30 °C. All the above steps included supplementation with 12.5 μg/ml chloramphenicol. BAC DNA was purified using NucleoBond BAC 100 kit (Machery-Nagel; 740579) following the manufacturer’s instructions.

### 2.2 Design and synthesis of sgRNA guides

sgRNA guide sequences were selected based on cutting efficiency score via the online tool guidescan.com [13]. IDT gBlocks were purchased to act as DNA templates for sgRNA synthesis, via *in vitro* transcription, with the following sequences (composed of T7 promoter, guide sequence (bold), and tracrRNA sequence; note guide should be preceded with GG (underlined) for efficient T7 synthesis, if not present at the 5′ end of the guide): sgRNA guide A template: CGTAATACGACTCACTATAGG**AACGTTTTAATCATTCACCG**GTTTTAGAGCTAGAAATAGCAAGTTAAAATAAGGCTAGTCCGTTATCAACTTGAAAAAGTGGCACCGAGTCGGTGCTTTT; sgRNA guide B template: CGTAATACGACTCACTATAGG**TTTCTGAATACAAAGCCTGG**GTTTTAGAGCTAGAAATAGCAAGTTAAAATAAGGCTAGTCCGTTATCAACTTGAAAAAGTGGCACCGAGTCGGTGCTTTT; sgRNA guide C template: CGTAATACGACTCACTATAGG**CATAACCAGAGAGTTCACTG**GTTTTAGAGCTAGAAATAGCAAGTTAAAATAAGGCTAGTCCGTTATCAACTTGAAAAAGTGGCACCGAGTCGGTGCTTTT

HiScribe T7 High Yield RNA Synthesis Kit (NEB, E2040S) was used to transcribe sgRNA in the following reaction: 1.5 μl 10X reaction buffer, 1.5 μl ATP, 1.5 μl GTP, 1.5 μl CTP, 1.5 μl UTP, 1.5 μl T7 RNA polymerase mix, 5 μl gBlock template, and 6 μl nuclease free water, incubated at 37 °C for 16 h. The sgRNA from the above reaction was then treated with DNAse I (Turbo DNAse, ThermoFisher; AM 2238) in a 100 μl reaction, following the manufacturer’s instructions. sgRNAs were purified using Megaclear Kit (Ambion, AM1908), quantified and stored at −80 °C. To assess RNA size and integrity, 500 ng of sgRNA was heated to 70 °C for 5 min, snap cooled on ice, and run on a 1% agarose gel at 7 V/cm for 10 min.

### 2.3 Sizing of repeats by CRISPR-Cas9 digestion

Purified BAC DNA was CRISPR-Cas9 digested in the following reaction: 0.5 μl Cas9 (3 ug/μl) (Takara Bio; 632640), 2 μl sgRNA guide A (150 ng/μl), 2 μl sgRNA guide B or C (150 ng/μl), 2 μl NEB Cas9 buffer, plus 5.5 μl endonuclease free water was incubated at 37 °C for 5 min for ribonucleoprotein assembly. Next, 2 μg BAC DNA, plus endonuclease free water was added to a total volume of 20 μl and incubated at 37 °C for 2 h. Digested BAC DNA was run on a 1% agarose gel pre-stained with GelGreen nucleic acid stain (Biotium; BT41005-5) at 7 V/cm for 2 h.

### 2.4 CRISPR-Cas9 cloning of *C9orf72* repeat region into pJazz

The CRISPR-Cas9 digestion reaction detailed above was upscaled in a linear fashion to digest 25 μg of BAC DNA from a clone harbouring a large repeat expansion, using sgRNA guides A + C. The 11 kb repeat band was excised under a blue light transilluminator (not UV light to prevent DNA damage), purified using NucleoSpin Gel and PCR Clean-up kit (Machery-Nagel; 740609), and blunt cloned into the pJazz-OC vector according to the BigEasy v2.0 Linear Cloning Kit (Lucigen; 43018) instructions. Briefly, a 10 μl ligation reaction was set up with 1 μl of vector, 1 μl of CloneSmart DNA Ligase, 1 μl of CloneDirect 10X Ligation Buffer (includes ATP), 2 μl H_2_O, and 5 μl (75 ng) of isolated repeat region DNA; incubated at 25 °C for 2 h, followed by inactivation at 70 °C for 15 min. 1 μl of the ligation reaction was electroporated into Big Easy-TSA Electro competent Cells (supplied with the kit) via *E. coli* Pulser™ Transformation Apparatus (Biorad) at 1.8 kV using 0.1 cm gap Gene Pulser/MicroPulser Cuvettes (Biorad; 1652089). Transformed cells were recovered in 975 μl recovery medium for 2 h at room temperature (RT), 150 rpm. The whole recovered culture was plated on 3 separate low salt LB-agar plates + 12.5 μg/ml chloramphenicol, 20 μg/ml X-gal, and 1 mM IPTG and incubated at RT for 60 h. Eight white colonies (indicating replacement of the pJazz *lacZ* stuffer cassette with insert) were picked into 5 ml low salt LB liquid culture + 12.5 μg/ml chloramphenicol and grown at RT, 150 rpm for 48 h. Vector DNA was isolated using QIAprep Spin Miniprep Kit (Qiagen; 27106), and screened via restriction enzyme digestion using BamHI and XbaI. DNA samples were heated to 65 °C for 5 min and chilled on ice before digestion, and before gel loading following enzymatic digestion, to reduce secondary structure formation. The following primers were used to Sanger sequence through the repeat from both sides (3 independent forward direction primers, F, and 3 reverse, R): F1, GCGTCAAACAGCGACAAGTT; F2, GCCCACGTAAAAGATGACGC; F3, CACCCTCTCTCCCCACTACT; R1, CAAGGAAGAGGCCAGATCCC; R2, AAGGAGACAGCTCGGGTACT; R3, ATGCAGGCAATTCCACCAGT. Glycerol stocks from repeat carrying clones were made by mixing 500 μl miniprep culture with 500 μl 50% glycerol and stored at −80 °C.

### 2.5 Sub-culturing and sub-cloning conditions to test stability of pJazz-*C9orf72*-repeat vectors

For subculturing, 500 μl original miniprep culture was split into 8x subcultures of 5 ml media (using low salt LB + 12.5 μg /ml chloramphenicol) for growth at RT, 150 rpm for 36 h, and screened by XbaI digestion. For sub-cloning, frozen material from glycerol stocks were streaked out using a sterile pipette tip onto low salt LB-agar plates (+12.5 μg/ml chloramphenicol) and grown in the below conditions (using low salt LB + 12.5 μg /ml chloramphenicol). Sub-clone vector DNA was extracted as described above, and screened by XbaI digestion. Condition 1: Incubate colonies on plate at RT, 72 h; 5 ml liquid culture, RT, 72 h, 150 rpm. Condition 2: Incubate colonies on plate at 30 °C, 24 h; 5 ml liquid culture, 30 °C, 16 h, 150 rpm. Condition 3: Incubate colonies on plate at 37 °C, 24 h; 5 ml liquid culture, 37 °C, 16 h, 150 rpm

### 2.6 Pacbio sequencing

PacBio sequencing, including library preparation and bioinformatics analysis, was performed by the Centre for Genomic Research, Institute of Integrative Biology, University of Liverpool, UK. BAC DNA was purified with 1x cleaned Ampure beads (Agencourt) and the quantity and quality was assessed using Nanodrop and Qubit assays. In addition, the Fragment Analyser (using a high sensitivity genomic kit -Agilent) was used to determine the average size of the DNA and the extent of degradation. This procedure was also used at the steps indicated below to determine average fragment size of the DNA. DNA samples were sheared with a Diagenode Megaruptor using short hydropores and a setting designed to create 8 kb fragments. Samples were DNA damage repaired and end repaired using the template preparation kit 1.0 from Pacific Biosciences. After an Ampure clean up, the samples were ligated to specific barcoded adapter sequences. After the ligase was destroyed by heating at 65 °C, the samples were treated with two exonucleases at 37 °C for an hour. The SMRTbell library was purified with 0.5x ampure beads. The library was size selected with 0.75% blue pippin cassettes (Sage) in a range 3.5 kb-50 kb.The recovered library had an average size of 7 kb. SMRTbell libraries were annealed to sequencing primer at values predetermined by the Binding Calculator (PacBio) and a complex made with the DNA Polymerase (P6 C4 chemistry). The complex was loaded by Magbead loading on a single RS11 SMRT cell. Sequencing was done using 360-minute movie times. Sequences were then imported into SMRT link software (version 5.0.1.9585) and assembled using the HGAP 4 pipeline, which includes a contig polishing step to remove sequencing errors. Sequences were also aligned to a human *C9orf72* reference to identify sequence variances using: (a) the Variance Calling pipeline included in the SMRT link software (version 5.0.1.9585); (b) bwa mem aligner (version 0.7.17) followed by FreeBayes (Garrison and Marth, version v1.1.0–60-gc15b070) to identify putative sequence variants. Variants encompassing the repeat region were further scrutinized to assess repeat lengths. As a final analysis, subreads were converted into circular consensus sequences (CCS), using options: ‘--minPredictedAccuracy = 0′ and at different numbers of minimum passes (0 to 5, e.g. option ‘--minPasses = 0′ for 0 passes), using the ‘ccs’ tool provided with the SMRTlink.

## 3 Results

### 3.1 Sizing and screening of *C9orf72* repeat-BAC clones

We began by obtaining a ~ 170 kb BAC vector derived from an ALS patient harbouring the *C9orf72* gene and ~800 GGGGCC hexanucleotide repeats [12]. The original study reported the highly unstable nature of the BAC in bacterial culture, with the majority of sub-clones exhibiting major retractions in repeat length. Southern blotting was the primary existing method to screen sub-clones for the presence of unretracted repeats, but this is a labour-intensive technique that does not facilitate a high-throughput approach for identifying clones and optimising bacterial growth conditions for repeat retention.

**Fig. 1:**
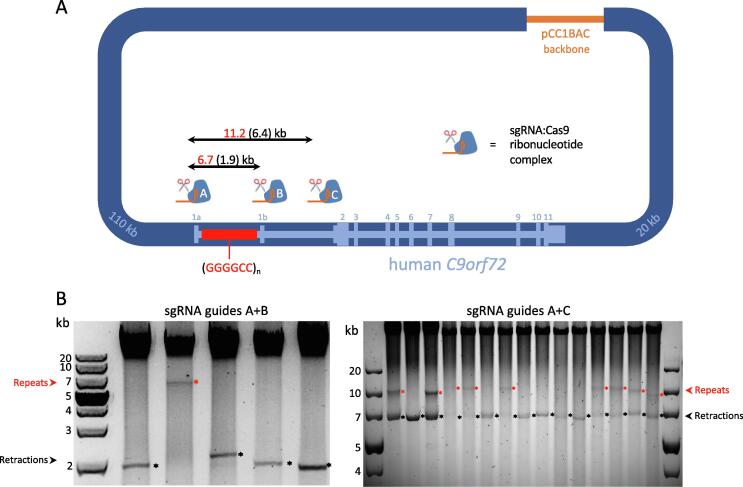
CRISPR/Cas9 digestion of *C9orf72*-ALS patient BAC. (A) Schematic of the *C9orf72*-ALS patient BAC vector, harbouring the *C9orf72* gene (light blue; exons numbered), a pathogenic GGGGCC repeat expansion in intron 1 (red), and large flanking genomic regions (dark blue; sizes indicated in light blue). sgRNA target sites A, B, and C are shown together with the expected size of the released fragments in red (and a fully retracted band size in brackets), following digestion with sgRNA A + B or A + C, assuming a (GGGGCC)_800_ repeat. (B) Agarose gel electrophoresis images of CRISPR-Cas9 digested *C9orf72*-BAC DNA sub-clones. Repeat bands are marked with red asterisks, large retractions are marked with black asterisks. (For interpretation of the references to colour in this figure legend, the reader is referred to the web version of this article.)

Instead, we developed a simple CRISPR-Cas9 based screening method (Fig. 1A). Specifically, we designed and synthesised 3 sgRNA guides; guide A (1 kb upstream of the repeat), guide B (840 bp downstream of the repeat), and guide C (5.4 kb downstream of the repeat). Digestion of BAC DNA using Cas9 protein and guides A + B or guides A + C releases a 6.7 kb band or a 11.2 kb band, respectively (assuming an unretracted repeat), for visualisation by agarose gel electrophoresis.

Screening with this method quickly and reliably identified clones of interest (Fig. 1B). Following identification of large-repeat harbouring clones, we sent a BAC DNA sample for PacBio sequencing to confirm the presence of a large repeat and to more accurately assess repeat length. Coverage across the repeat was poor, with only a single read spanning the large repeat, which was revealed to be 728 repeats in length, with 100% GC content, and with 95% of the repeat region reading GGGGCC, and interruptions constituting gain or loss of single C or G nucleotides (Supplementary Data 1). Table 1 outlines repeat retention rates in BAC sub-clones, highlighting that the majority of clones underwent major retractions. However, even in cases where a large repeat was detected, these clones were heterogeneous in nature and a retracted band close or equal to wild type in size was also present in the vast majority of cases (Fig. 1B).

**Table 1:** Summary of repeat retention rates following screening of *C9orf72* BAC subclones.

Total sub-clones screened	Clones with 500–800 repeats, and no retracted band present	Heterogeneous clones with 500–800 repeats, plus repeat at or close to WT size (estimated < 50)	Heterogeneous clones with 300–500 repeats, plus repeat at or close to WT size (estimated < 50)	Clones only with repeat at or close to WT size (estimated < 50)
108	2 (2%)	31 (29%)	8 (7%)	67 (62%)

### 3.2 CRISPR-Cas9 cloning of *C9orf72* repeat region into the pJazz vector

**Fig. 2:**
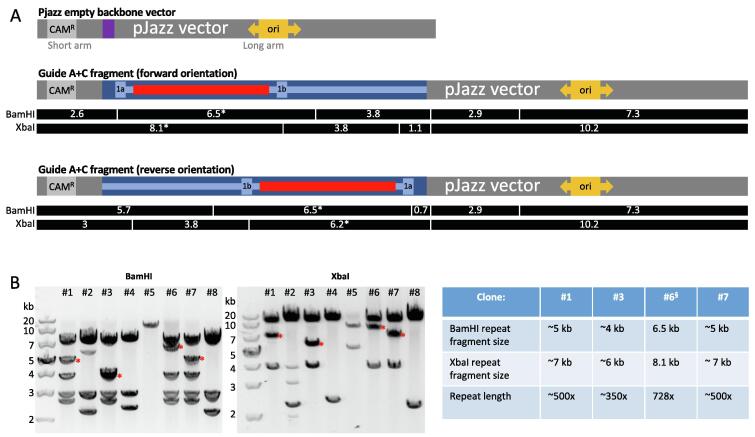
CRISPR-Cas9 cloning of *C9orf72* repeat into pJazz. (A) Schematic of the pJazz cloning vector showing chloramphenicol resistance cassette (CAM^R^) on the short arm, *lacZ* stuffer cassette (purple), and origin of replication (ori) on the long arm. sgRNA guides A + C were used to release the *C9orf72* repeat (red) containing region, which was gel purified and blunt-cloned into pJazz (replacing the *lacZ* casette). The repeat containing fragment is illustrated in two possible orientations within pJazz (exons 1a and 1b added for reference), with expected BamHI and XbaI restriction fragment sizes shown below, assuming a 728x repeat. (B) Agarose gel electrophoresis gel images showing digestion of miniprepped clones with BamHI and XbaI, revealing 4 clones carrying a repeat in the forward orientation (#1, #3, #6, #7); asterisks indicate repeat containing band. The table displays the estimated repeat sizes for clones #1, #3, #6, #7; it is assumed clone #6 is unretracted^§^. (For interpretation of the references to colour in this figure legend, the reader is referred to the web version of this article.)

Due to the instability of the *C9orf72* repeat within a large BAC vector, we hypothesised that cloning the repeat region into a smaller total vector size may improve repeat retention. The pJazz vector (Lucigen) is a linear vector that does not supercoil, or suffer supercoiling-associated torsional stress, and is reportedly capable of stabilising repetitive, unstable sequences, including 220x CGG repeats from the Fragile X microsatellite repeat [14], although larger 100% GC-content repeat sequences were not reported. Since we had already developed a strategy to release the *C9orf72* repeat from the BAC using CRISPR-Cas9 (Fig. 1), and Cas9 cleaves DNA at least in part with blunt ends [15], [16], we attempted to blunt-clone our CRISPR-Cas9 guide A + C fragment into pJazz (Fig. 2A). We chose CRISPR-Cas9 guide pair A + C simply because it cleaves a proportionately larger fragment (11 kb) from the 180 Kb BAC (compared to A + B; 7 kb), which can be better visualised in an agarose gel for subsequent isolation.

Fig. 2B shows the results of this cloning step, with 8 clones screened via BamHI and XbaI digestion. Clone #6 exhibited the correct band patterns based on a 728x repeat inserted into pJazz in the forward orientation, while clones #1, #3, and #7 exhibited the correct band patterns for the forward orientation, but with 1–2 kb smaller repeat-band sizes, approximately 350x (#3) and 500x (#1, 7) repeats in length. We did not generate clones carrying repeats of any length in the reverse orientation; with the remaining four clones representing rearrangements that we were unable to resolve.

**Fig. 3:**
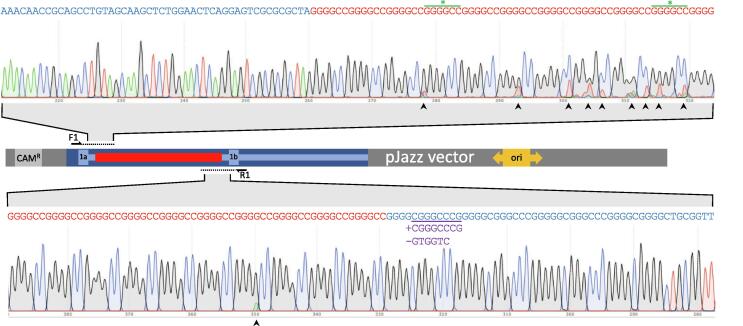
Sanger sequencing through 5′ and 3′ repeat junctions from pJazz-*C9orf72*-repeat clone #6. Sequencing chromatograms via sequencing primers F1 (top) and R1 (bottom) are shown, revealing the presence of human intron 1 (blue text) correctly juxtaposed to hexanucleotide GGGGCC repeats (red text). Green asterisks represent 2 hexanucleotide sites that read as GGGCC (missing G) in PacBio sequencing. Purple text highlights the insertion/deletion event (−GTGGTC + CGGGCCCG) downstream of the repeat. Arrowheads indicate erroneous lower peaks; independent primer sets (Supplementary Data 2) show these to be in random positions, indicating sequencing noise. (For interpretation of the references to colour in this figure legend, the reader is referred to the web version of this article.)

With the repeat cloned into a smaller vector we were able to investigate the purity of the repeat further. Sanger sequencing using several independent primer sets flanking the repeat region confirmed the presence of the repeat in all four repeat-containing pJazz clones (reading ~350 bp into the repeat sequence, with increasing noise due to the difficulty in sequencing such regions) (Fig. 3 and Supplementary Data 2). The use of independent primer sets confirms that erroneous lower peaks, observed in Sanger plots close to the 5′ and 3′ junctions of the repeat region, are in random positions and most likely represent sequencing noise (e.g., compare files a and b in Supplementary Data 2). According to PacBio sequencing of the original BAC vector, 5% of the repeat contained interruptions to the GGGGCC sequence (Supplementary Data 1); close to the 5′ end of the repeats, two individual GGGCC (i.e. missing G) sites were called, which were in range of Sanger sequencing capabilities. Sanger sequencing showed these PacBio calls to be errors (i.e. Sanger sequencing called the equivalent positions as GGGGCC) (Fig. 3 and Supplementary Data 2). At two other sites within the repeat, additional C bases were called by PacBio, generating ApaI (GGGCCC) restriction sites. Digestion with these enzymes did not reveal a band pattern consistent with this scenario, but rather yielded a pattern without the addition of these sites (Supplementary Fig. 1). Therefore, the four sites we scrutinised appear to be PacBio sequencing errors rather than true repeat interruptions. We cannot account for the remaining potential repeat interruptions at the time of writing, although long-read sequencing is known to be error prone. Finally, both PacBio BAC sequencing, and Sanger sequencing of all four repeat containing pJazz clones, showed the presence of an insertion/deletion event immediately 3′ to the repeat (-GTGGTC + CGGGCCCG) (Fig. 3 and Supplementary Data 2), similar in nature to that seen previously in a subset of patients [17], [18], [19].

### 3.3 Stability assessment of the *C9orf72* repeat in the pJazz vector

**Fig. 4:**
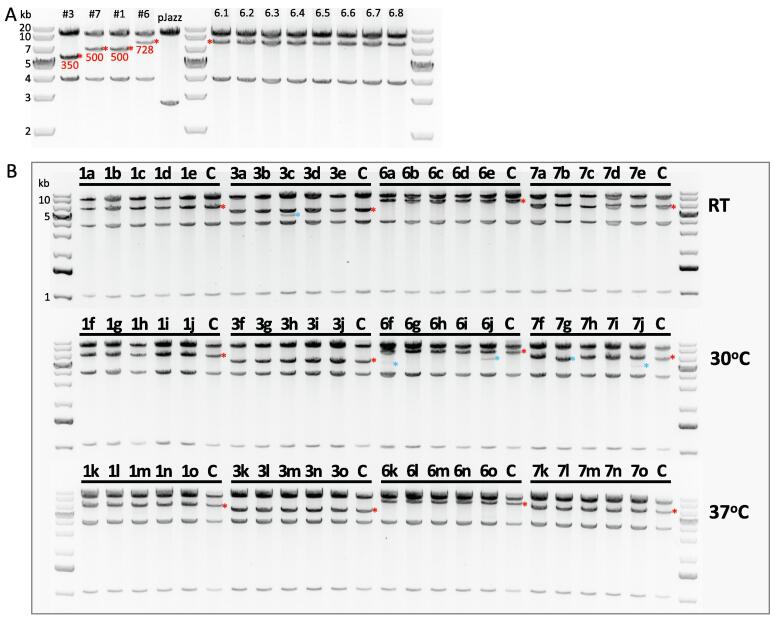
Stability assessments of the *C9orf72* repeat inside pJazz. (A) 500 μl clone #6 was split into 8x 5 ml subcultures, denoted 6.1–6.8, and purified DNA was digested with XbaI. Clones #3 (~350 repeats), 7 (~500 repeats), 1 (~500 repeats), and 6 (728 repeats, assuming unretracted), plus pJazz vector were run as controls in size order (left). Red asterisks indicate repeat bands, with approximate repeat lengths indicated in red text. (B) 15 sub-clones from each original clone carrying repeats were subjected to growth at different temperatures. Sub-clones 3c, 6j, 7g, 7j displayed evidence of retraction (blue asterisk). DNA from the original clones were included as controls, indicated by C, with control repeat bands indicated by red asterisks. (For interpretation of the references to colour in this figure legend, the reader is referred to the web version of this article.)

500 μl culture from clone #6 was split into 8x 5 ml subcultures for extended growth, and the repeat remained intact (Fig. 4A). To further analyse repeat stability, glycerol stocks from clones #1, #3, #6, and #7 were streaked-out and grown at RT, 30 °C, or 37 °C. 15 colonies from each clone were grown at these 3 temperatures, and extracted DNA was again analysed via XbaI restriction digestion (Fig. 4B). We saw only minimal evidence of retractions (5/60 total sub-clones), during the screening of clones by restriction digestion. For the largest repeat (#6 sub-clones), 100% of sub-clones retained the repeat across all conditions; with minor evidence of retractions in 2/15 clones (only at 30 °C growth). Where present, retractions typically were not complete and only represented a small fraction of total DNA, with the exception of sub-clone 7g, which showed homogenous retraction of < 1 kb in repeat loss. Surprisingly, 37 °C represented the most stable temperature, with no evidence of retraction at all across all subclones, showing that reducing growth temperature in this context does not have an appreciable positive effect on repeat length stability.

## 4 Discussion

**Fig. 5:**
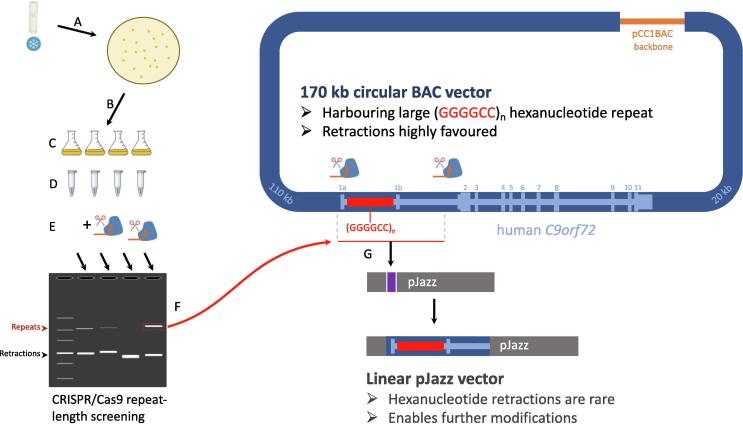
Summary workflow to screen and subclone *C9orf72* repeat region. (A) Frozen glycerol stock of repeat-BAC harbouring DH10b E. coli bacteria is streaked out onto LB-Agar plates. (B) Colonies are picked, and (C) grown in liquid culture for (D) BAC DNA extraction and purification. (E) BAC DNA is CRISPR/Cas9 digested to release the repeat region and screen for subclones that harbour large repeats. (F) Large repeat containing bands can be excised from the gel, purified and (G) blunt cloned into pJazz vector for stabilisation and further cloning steps if required.

Here we present methodology to manage and manipulate large repetitive sequences, especially those present in large vectors such as bacterial artificial chromosomes derived from patient DNA. Our simple CRISPR-Cas9 based methods serve to both screen for repeat retention and as a means to clone regions of interest into alternative vectors (workflow summarised in Fig. 5). Whilst this approach likely lacks the sensitivity to visualise repeat regions from genomic DNA of patients via gel electrophoresis, targeted CRISPR/Cas9 digestion of mammalian genomic DNA has the potential for cloning such regions from genomic DNA, as has already been shown for cloning large bacterial genomic regions [20]. The pJazz vector system has been shown capable of maintaining both AT and GC rich sequences, including repetitive GC rich sequences several hundred nucleotides in length [14]. Here, we demonstrate the capability of this vector system to stably harbour repetitive GGGGCC sequences, several thousand nucleotides in length, derived from a *C9orf72*-ALS patient.

**Fig. 6:**
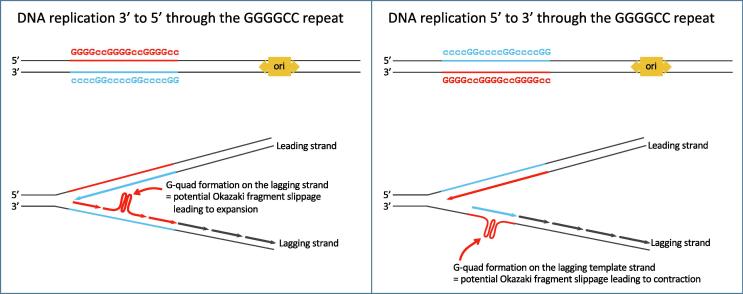
Schematic to demonstrate the potential directional impact of DNA replication on repeat stability. Lagging strand DNA synthesis is prone to slippage events when repetitive DNA is present. On the left, DNA replication is shown to run through the G-rich GGGGCC repeat strand (red) in a 3′ to 5′ direction, resulting in G-quadruplexes forming on the lagging synthesis strand, which can lead to expansion events. On the right, DNA replication is shown to run through the GGGGCC sequence in a 5′ to 3′ direction resulting in G-quadruplexes forming on the lagging template strand, which can lead to expansion events. Blue text represents the antisense CCCCGG repeat strand. Origin of replication is denoted by ori. (For interpretation of the references to colour in this figure legend, the reader is referred to the web version of this article.)

Interestingly, we only achieved success cloning the repeat in a single orientation, consistent with previous reports that the directionality of replication impacts stability of microsatellite repeats [21], [22], including bacterial studies using short GGGGCC repeats within circular plasmids with unidirectional origins of replication [23], [24]. DNA replication is asymmetric on leading and lagging synthesis strands, with continuous synthesis on the leading strand; and discontinuous synthesis and the formation of Okazaki fragments on the lagging strand, which is vulnerable to DNA polymerase pausing and strand slippage in the presence of repeat sequences. Specifically, strand slippage can purportedly result in expansions when G-rich G-quadruplex structures form on the lagging strand, or can result in contractions when G-rich G-quadruplex structures form on the lagging template strand (Fig. 6) [21], [22]. Both strands of the *C9orf72* repeat sequence (GGGGCC and CCCCGG) form G-quadruplex structures, but the G-rich strand forms a more thermostable structure [25], [26]. Due to the linear nature of the pJazz vector used in this study, the bidirectional origin of replication, on the long arm of the vector, only passes through the cloned region from one direction; in the case of all our repeat carrying clones, in a 3′ to 5′ direction through the GGGGCC repeat, placing the G-quadruplex forming G-rich sequence on the lagging strand of replicating DNA, whereas clones with the repeat in the reverse orientation would place the G-quadruplex forming G-rich sequence on the lagging template strand. Therefore, our repeat carrying clones may be prone to expansion events, although we did not observe this, which may suggest these events are rare and/or place the bacterial clones at a growth disadvantage. Repeat carrying clones in the reverse orientation would be prone to contraction events, which could explain why such clones were not observed. We have previously found that shorter GGGGCC repeats are more stable than the reverse GGCCCC repeats in standard bacterial cloning vectors and that reversing the origin of replication enabled stable cloning of GGCCCC repeats [24]. The large, circular *C9orf72* BAC vector used in this study contains a unidirectional Ori2 bacterial origin of replication (and also a transactivatable bidirectional origin for high copy number initiation that we did not utilise) that passes through the repeat in the 5′ to 3′ direction, placing the quadruplex forming G-rich sequence on the lagging strand, which favours contraction events, likely contributing to the instability of repeats observed, although vector linearity also contributes towards stabilisation [14].

Origin of replication sites, and their differential usage, have been linked to repeat instability in the human genome at the *FMR1* locus and its associated CGG repeat, causing Fragile X syndrome; specifically, preferential use of a downstream origin versus an upstream origin, very early in development in fragile X cases, is thought to lead to a net gain in expansion events [27]. Genome-wide mapping of human genome origins of replication places origins of replication both at the *C9orf72* promoter and immediately downstream of the gene [28], and it is tempting to speculate whether differential usage of these origins impact repeat length and stability, and whether genetic factors are involved. Since the *C9orf72* promoter is in the vicinity of the repeat region, existing *C9orf72* BAC transgenic mouse models (and including the BAC used in this study) likely include this upstream origin of replication. Some BAC transgenic models also include the downstream *C9orf72* origin region, but others do not; in addition, different BAC models have distinct genomic insertion sites and therefore will be within varying proximities to different origins of replication [9], [10], [11], [12]. These latter variables may lead to differences in repeat stability among models and may explain the observed model-to-model differences in phenotype. Thus, modelling repeat instability in animals may require a wider physiological genomic context, beyond the boundaries of the repeat, for maximum physiological relevance. Overall, our methodology presented here greatly increases our capability to model *C9orf72*-associated disease *in vitro* and *in vivo*.

Funding

This work was funded by the Medical Research Council.

## CRediT authorship contribution statement

**Remya R. Nair:** Methodology, Investigation, Writing - original draft, Writing - review & editing. **Charlotte Tibbit:** Investigation, Writing - review & editing. **David Thompson:** Investigation, Writing - review & editing. **Ross McLeod:** Investigation, Writing - review & editing. **Asif Nakhuda:** Investigation, Writing - review & editing. **Michelle M. Simon:** Writing - review & editing, Formal analysis. **Robert H. Baloh:** Conceptualization, Resources, Writing - review & editing. **Elizabeth M.C. Fisher:** Conceptualization, Writing - review & editing, Supervision, Funding acquisition. **Adrian M. Isaacs:** Conceptualization, Writing - review & editing, Supervision. **Thomas J. Cunningham:** Conceptualization, Methodology, Writing - original draft, Writing - review & editing, Formal analysis, Supervision, Project administration.
