# Supplementary material for: Sizing, stabilising, and cloning repeat-expansions for gene targeting constructs
Source: Methods. 2021 Jul;191:15–22. doi: 10.1016/j.ymeth.2020.07.007 (PMC8215685; doi:10.1016/j.ymeth.2020.07.007)
Supplement: Supplementary data 1 [file mmc1.docx]

**Supplementary Data 1.** Consensus sequence of *C9orf72*-BAC vector plus 728 repeats, deduced from PacBio long-read sequencing. Repeat is highlighted in red on page 14. Interruptions to the hexanucleotide GGGGCC sequence are in green. C (cytosine) insertions that generate ApaI restriction sites, but shown by restriction digest to not be present (sequencing errors), are highlighted in yellow. Two non-hexanucleotide sequences at the 5’ end of the repeat, also shown to be errors (i.e. true GGGGCC sequences) by Sanger sequencing, are highlighted in grey. Orange text represents the BAC vector backbone.

AAGCTTCAAATAGGGACAAGCTCCTGACTGCTCAGTGAGGGAGACTGAGAAGATTAAATGGAACATGGCACTGAAATGAGCCAGAATAGGAATTCAAGAACCTTTAATTACAGAAATTAAAAGAAATATTAAGAATGATTGCGAACGACCAGAGTATAGAATTATTATCTCTACTTCCCCCAAACACACAGTACCTTCTATTTTACAGAGTACTGTGAGACTGAGAAAGTGTTTACATATTCGTCTCCCTCATTCCTGATGACAGTCCCTGTGTTGTGGTATGGTAGGCATCACTACCCCATTTTAAATGTGGGAATATTGAGAGCTTTAGAAGTACAGTGAGGTGCCCACAGCAACAAAACTAGTAAGTGATCCAACTGAGACTCAAACCCAAGCCTAGTTTTTTTTTTCCACTGTGTAATAAAAAACTTAAAAAAAATGGAAACAGTAACACTTAAAACATCTTCCCTATAATTCTCCGGCATAAAGGCCAAGGTGCCAGGTGTCATTGAACTTAGAAAGGGATAAGCAAATTGGTGGTTGCTATGGAGTCACCTTAGCACTTCAGTGTTTTTGAAGTTGGGGTTGACACTGAAGGCTATTAAAGTTAAAGGGAAACATGAGCCAATACTTATGTATGTTTCTAAGTAATGACAAAAATATGACATGATTCTCATGGAACTGGAAAAAGTAGTTCAAAATGCTACTAGATAAAGGGAGAGATTTAATGGAGGAATCAGAATAAATCAGTAGATAGAGCCTTTGATGAGAAATAATCATTTTGAAACAATTATGTAAAATAAAAACTATAAATCATGAATATAGAATAAAATACCATTTGTTGTTACCAAAGAGAAAAGTCAGAAATGATGAAATGTAGGTAGGAGACCTAAAAGATTTTTAAAAATGCAAATACATAGATTATTACTGATCACAGAGGCAATAAGAGATTAGTATAAAGTTGCAGAAAACTCTAAAGCTGTTTGATAATTAGTTTTTCCTCAGAAATTGGAAATGTGTATTATTGAAGAGGTCCTTAATGTCACTAGATGTAGCCCATACTGCAAATCTGTTGGAAAATAAACAGGAAGAAAGACTTGTACCTGTGCTACACTTAATATCAGAGTTATTTGCTTTGAAATAACTTGAAGACTAGGAATAAATAAATTGGGGTAGGTCTCATTTCTGTGATCTGATACTAGACATGGGCCTGGTTTCTCAGAAAGCACTCACTAGGTGCGGTGGCTCACACTTGTAATCCCAGCATTTTGGGAGGCCGAGGTGGAAGGATTGCTTGAGCACAGGAGTTCATGATCAGCCTGGGCAACATGGTGAAACCCCATCTCTACAAAAAGTACAAAAAATTAGCCAGACACGGTAGTGCATGCCTGTAGTTCCAGCTATTCTGGAGCTTAGGTGGGAAAATCATTTGAGCCCAAGAGATGGAGCTTCCAGTGAGCTGTGATTGCACCACTGCACTCCAGCACCTGTGTGACAGAGCGCGACCTTGTCTCAAAATAATAAAAATAAAGCACTCACTGGGGGTAGCACAAAGGGTAATTCAGGAGATGAGATGTGCTTCAAAAGAGTTACAAGGAGAAAAAAATTAACTAAAAGACCTTGATGGAAGAAAGGACCTATGGAACTCTAACGTGTTTAATAAAGAATGCCAAAACATAGCAAGATCTCATCTCTACAAAAAAAAAAAAAAATTTTTTTTTAACCACATCTAGGATGTGGTATGGCGGTACATGCCTATAGTCCCAGCTACTTGGGAGGCTGAGGTGGGAGGATTATTTGAGCCCAGGAGTTTGAGGCAGCTGTGAGATATGATTGCAGCACTGCACTCTAGCCTGGATGACAGAACGAGATCCTGTCTCAAAAAAAAAAAGAATGCTGACATCAAATTCCATCCTGTGAGACAGAGCATCATATAAAGGGCACAACGAGTAGACTCTCAGAAATCTGGACCCAGAGACTCAGAAAGGCTGCCACTGGTTAAAAACAGCAGATTATTTAGTTGTTAATATGTGGAGGATTGCCTCTGTCTACTCCCAGAGAAACCCCAAGCAACCTGAGGAGAAAAACAAATGCAAAGGAGACACAGGCTCTGCAAACCATTTTCAACAGCCATTACCCTCCCCTGGGTCAGCCCTCACATTGAGCAATGATTTAGATACAAATCAAGAGAGAATGCCGATCAAATTATTTGAGGAAATAGCAAATATGTTAAATAACAGAGGTGCGATAAAAAGAAATGGCCTTGAAAGAGAAGAAACTGGTAAGATTAAATTCAATAGAGCTAAGTGTATTACTATGCCAAATGTAGGTTCAGAAAACCAGTTATGCAACTTGAGGGAAGAAATTTAAGAGCAATGTATAGGAAAATGAACTCCTTGTTTGAACTGGCTACAAGCTCAATGTCAGTCTACCTTTAATACAGTGATTCTTAACCTCTTTGGGAAAACATTCCTTTGGGAACATGATAAAAATTGAGTACATTCACCTTAGAAACACATATTTTTATACAGCATTTTGCACTTAGTTTCAGAAAGCTCATGGAAGTCTCATGAGGCATCCATTTATAGTGTGATGGTTTAAAATATGAGACCTGTGCTAAACAAATCTACAGTCAGTCAAGGACCCCGTCATGTCTCTGCCACTCAGAATTTCTTATTATGGTTTTTAAAAAGTAAGATAACCTCTATCCACTCTGAGTTTCCTCATCTGTAAAGGGGAGATAATAAGATAATAATACTTGCCCTCTGGTGTTATTATAAAAGATTAATAAGATAAAGCACCAATAGCTTTTATTTTTGTCATAATTATCATTTTATTATATTTTAAAATATTAATATATTTTTATTATATGTATATTTCATCATATGTATTATATTACCAAAGTCAGTTGCTACCTTATGTTAACTTTTAAATAACTTTTAGGTTTGGCTTACAGGACAAAAGAAAGGGTTGCTCCCCTGGGCTGCCTGCCACTTAAAACAATATCTGGAGCTTTTAGCTCTGATCTTGGCATCTTGTTTTAAGAAACACAGGTGTCACCTAGAACACAAGTAAAGGAGGCCAGCCAAGATAGTAATGAGATACATAGGGTATTTACAAGAAGACAAGTGGAAGAATTGGGGATCTTTGACCTAGCAAAAGGAGGATTTAACTAAATTCAACTACTTCAAATGCTATTGTGCCAGAAGAAGAAAAATTATTCTGAATTATTTCAGTGGCCAGAATTTGGGCCAAAAGCGTAGGAAACTTCAGCTACATGTAAGGAAGGCTGTCTTAGAAATTAGATGCCAGCATTTCTTCATCACTGAGGGATCAACATATTCATTCATTCAACAAACATTAATTAAGCATCCACTGTGCACCAGAGACCATGCACGTTACTGGGGATACAAAGATGAGTGAGGCACAGATCTTGCTCCTTTAATAGACTCCAAGTTTGGTGGGAGACACAGGTAAAATAAGTAATGACAATGCAAACAGAGGCAATGGTATTGCCTGGAAAACAAGGCAGATATCTACTAAGAAAAGAGTTGAGCTATAATAGATTAAAAGAAACAATCTAGGAACTACGCTAGTCATGCTAGGCAGAAGACAAAAGGCACAAAGGCAAGAAACAGCATGATGTATTCAAGAAATTCTGGGCACAAAGGAAAAGGCAAAAAAAAAAAAAAAAAAAAAAAAAACAAGGAGAGAGCTTAGATGTGATCCTATGAGCTCAATAAAGCCATTACAGGATTTTAGGCAGGGGAGTGACCTGGTCAAAACTGTGTTTAGGTATGGATGTATCATTACTAAATATCTGGACACAGATCTTATATTTGTGATATTTCTACATGTTCCCATGAGAGGTCGTTAAACTAGTTAAGTAAGGCACTTCATGAGAGTTCAACATTTTTTCAATGGGTTACTTAGACTTGTGTTATACAAGAATGTTTCAAATGCGCTGCTTGTTTTGGGTATTGAGCCAAGTTGACATCTAAAAGCAAAATAAGAGACAGAGGTAAGAATCCAGGCAAGAGACATAAAGGCCTGGATTAGGTCTGCAAAGCTGTAGAAGGATTGGGAGATGGCACTGATACTCTCCGGGATCTTTCCAAAGCTTGGACTCTATGCAGTTCAGCCCTGACACAATCACAGAGTTGTAGAATTGTAAGGGACACTGACACCATCTCATCCAAGTCTTCCATTACAGAGACTAAACCCCAGGAAGAAAAAGTAGCTTTCTCCCTTCTCCATAGAGCAAGGTAATAAAGAAGTTAAAACTCTGAAAAGCATCACCCTTCAACCTTGATCCAGTGAGTGACCTCTTATCCTGCCACATCACGCAGATTTTCATTAAATCTTTTTTGTAGCATATACACTTTGTTAATTAAGCCTACAAAAACAGGGTGCTAGGATATAAGCTGTTCGTCAACTGGAAAAAAGCAAAGAATTTAAATGTTGCCATGAATTACAGGTATCTTATGAAGCAGGAAGGGTCTCCTATTTAAACTTCTCAGCATAGCATACAAAGCAAAGGATGATTCAAAGCTGAGTCCTCCATTTGCCAACAAAGTGGCATTGAGGAAGTCACTTAACCTCTGAAGCTCATCTGTAAAATGAATAAATAATTCTCACCCTAAAAGTTGTAGATATATATAATGCCTTCACAATGTATAGAGCCCAGCGCACGTTAGGTAGGTCATTTATTTCTCTGTTCCTTCCTTCCTTTCCAAATTTTGCCTTCAAAATTTGTAAGGAGAAGTTATCTGCCATCTTCTCCAGCCCACTAGAGACAGTACAGCAAACGAGTTAGAATTTGGCCTCTTGTGTCAGAAAAACTAAATGGTGACTCTTGGCCCTACATGAACACAGACACATTCTTCAGTCTCTCATGACTGTTGATACATCTATGATAATCTGAAGACAACTATTGCAACCTCATAGGGTTGTTGTGAGGATTATATGAGCTAAATATATGAAAGCTTCTTAGAGTAATGCCTGACATAGAGTAAACACTCAAGTTATGGCTTGATTATGTTGACTTTGCATGCAGAGAACCACGTTACATTGTAATCTCCCTTATCTTCTCATCCTCATAGCTAATGATCTTCTCTTTCATACACAACTTCATATTTTCAAGAAAATTACATTTTCTTCATGCATGTCTCTTCAGACATGGAAGAAGCTAAAAAACCTCTTTTCTCAAAAGTAGTTAAGGTAAATGAGATCAATGGATCCAACTGACTGCCAGAAATGGAGTAGTGGAGCCCTTAATATCATCAATTCAAGATATTGGACACCCATCCATTGATTTTCTCCCTGTCTCTATTAGAAGTCAATAGCTCCTCATCACACATTCGACAGGTGAGGATTCCTAGAAGTGCTGTAAGTGGACTGAGGTAGTTTGGTGTGCCTGTGTCCAGAGCATTAGGTGAGAATTAGAATTGACCTCTGTTTATACCATCCTCTATCCCACACACCTTATAGGGACTCGAAAAACATCCACTGATCCATGAACCTTTCCTCACTGAGTGCCTTGGTAATTAGGACATGATTAACTCTAGGGGCTACCTTGCATTGCATTTGAGAACACAGGACTAAAGTGGATGGCAAAAAAATAATGAGTAGGAGATTGGGCTCCATGGTGAGACCATTTGAATCCTGGCTTCATCATATTTGTAGCTTTAGGCATGTCACTGACTTTCTCCAAATCCCAATTTTCTCTTCTATAAAATTAGGAGCATAACAGGTATACTAAACTAGCAGAGTTCTCATGAGATTAAGTGAAAGAATTTTTGTAAAGAGTTTAGCACCAAGCAAATGTTCAGTAAGTGCTCTTACTGTGATTAGTTCAATATAAAAATGACTAACTGCTGTATCATGTTATATTGCTTCAAAATGCAAAAATAAGAATTTACAGAGTGCGAGATTACTGTGGCACTAACGAGAGTATACACATATTAACTAGGGAGATGTTTCTTAAACAAGATAGGTTCTGACCTAGATCTTAGAGAATAAATAGGTTTAGATCTGCAAAGGGCTCTCCCCATGGGCAAAAGAGCCAGGAGGATAAAATCCCTGAGTATGCAGAAAAATAAGTGAACAGTCAGAATTTTGAATAAGTACCCAAAGGCTAGAAAAGGAACATTTCAAAATCCACTATGGAAGTGCCAGGCAATTTCAGAAAAGATGAGCTATCTAGTGATAATAACTGGCTGATCAAAACCATAGTGTGGTAGGTAAGCAAAGGTTTTAGCAGCAGACAGGCCTAAGTATAAATCACAGACAAACTACCTAACATTTCTAAGCCTCACTAGTCCCGTGTCTGGAATTGGTTCCTTGCAGTGGGTTCTTGGTCTCGCTGACTTCAAGAATGAAGCCGCGGACCCTCCTGGTGAGTGTTACAGTTCTTAAAGATGGTGTGTCCGGAGTTTGTTCCTTCAGATGTTCAGATGTGTCCAGAGTTTCTTCCTTCCAGTGGGTTCGTGGTCTCGCTTGACCTCAGGAGTGAAGCCACAGACCTTTGCAGTGAGTGTTACAGCTCTCAAAGGTGGCGCCTCTGGAGTTGTTTGTTTCTCCCAGTGGGTTCGTGGTCTCGCTGACTTAAGCAGTGAAGCCGCAGACCTTCACAGTGAGTGTTACAGAGCTCATAAAGGTAGTGCAGACCCAAAGAGTGAGCAGCAGCAAGATTTATCGTGGAGAGTGAAAGAACAAAGTTTCCACAGCGTGGAAGGGGACCCTCGTGGGTTCCGCTGCTAGCTCGGGTGGCCAGTTTTTATTCCCTTATTGGGCCACACCCATATCCTGCTGATTGGTCCATTTTACAGAGAGCCGATTGCTCCATTTTACAGAGTGCTGATTGGTGCATTTACAAACTTTTAGCTAGACACAGAGTGCCGATTGGTGCGTTTTTACAGAGTGCGAACTGGTGCATTTACAAACCTTTAGCTAGACCCAGAGCGCTGACTGGTGTGTTTACAATCCTCTAGCTAGACAGAAAAGTTCTCCAAGTCCCCACTCAACCCAGAAGCCCAGCCGGCTTCACTTCTCAGTCCCATCTTTGTTATGAAATTATTTTCACCTACCTTGCAAAATTTTGGGGAGAATTAAATGAGCTGTTGAAGGTAAAACAGTCATCTCAGGGTCTGACTCAGTGTCAGCTAGCGTGATTGCCTAGTGATATTCTAAGAAAAACAAATACTAAAAAAAGTTATTTAAATTTACCTGGACAAACACCAAAGTGGGGTTGCCTAACTGAGGCAGGAGAATAAGGTCTGGAGGTAGGGCACTTAAGACCAATTCATGCTGAATCAAAGAAAAACACCTTGATTTCCTAGGCGCAGGGAATCTGAGGCCAATTTGTGCTGACTTCCTAAAATAGAAAACACCAAGGTCTGGGATTGGGGAATCTGAGGCCAATTCCTGCTGACTTCCCAAAGCTGGATCAAAAGGAAAACACCTGGGTCTAGGGGCAGGGAACCTAAGGCCAATTAACGCAAACTTCCTAAAGCTAAACCCAGAGGAAAAACCCCATCGCCCCAGGCCAAGTAACAAAGGATCAAAGGCTACTCTCCCTATAGCCCTCCCCATTCCACCACATCTCAGATGGAAAGGGAGATTGCCTCGGATTGACCGTAGGCCTAGCAAGGACCATCCCTTCGTGTGCATAGAGCGCCAATTCACCTCAGCCTTATAATTAGCCAAAGACCAAATCCTTCATTTCAGATAACAGGTAACCAATAGAGTCCTCAAAAAAAGTACTTAAAACCCAGAAAACCTTGTAACCGGGCCCTTTAGCCTCTTGCTCCGGCCCACTTCCACCCCGTGGAGTGCTTTCTTGCTTTAATAAAGTCCTGCTTTTGCTGCTCCCTTCCTGTGTTTTGTTCCTTTGTTACTTTGTGCGTTTTGTCCGGTTCTTTGTTCAAAACGCCAAGGACCTGGACATTTACACTCAAGGCCCTCCTTCCGGTAATATAACTGTAGAAGAATCAGGGGAAGTTTTATCCTCATTCAGTGTCCTAAATGTTCTTACTGGTTTCTGTAGGTATCTTATACATAGTAAAATTTCAGTGTTCAATAAATACAAGGCACTGTCATCATGAATAGACAGAGCTAATTGTTACTCCACTTAATCCTCACACAACCCTGGAACAAAGATAATTTTATTTGTCCTGTCTTACACATTTACAGAGCTTAGTCACTTATGCAAGCTCACACAGAAAATAGGGAGTAGAACTAGAATTTGAGCCAAAGCAGCCTGTCTCCAAAGACTGCGTGCTTTGTGTGCCTCTGTAGACTGCCAGGAGATAACTCTGAAACCTTCAGACATGTTCCTGCTGATCTGAACCTGCTATGACAGTGTGAGCCCACCACCTGAATTCAAGAAGGACAAAAAAAAAAAAAAAAAAAAAAATAGCCCAACAGGGAAAAGGAGACATGATTAGGTAGCTGGCTGGATAGAAAGTAGGCTGACAGAATCACCTGCAATTTTCCTTTCAATCTTTCTCTGTAATTTAATTCTTATTTGAATTCATAGTGAACAGCCCTTACACTTAAGGGTGCTGCAGATCTTGCCTCTGCTTCAGGGCAAAGTGCTTGTTGGAGCTATTGCCCTGTAATGACCGAGAGACAGAATGCCTCCAAAAAACAAAATTCTTAAGTAGGTCTGGTTGTGCCAAGTGATATTAATTACACAATCCATACTAGCACTATCAACTGCAAACTTACCTGGAAGAAGGGCTTGCCCCCTTAGATACTACTTAGAAACTATTTGCTCCTCCTGTCAGTGCCAGAAGTAACTGAAAATCGAATTTGAGAACATTATGGAAAATCCAGAGAGGATTCAGTAAAGGAGGGGGGAAGTTAAACCATTGGTTCTCAATTTTGTTTTCTTTTAAACTTTCCCCATACATTCTCCTCAGGTGTCCCATAACAAATCCAACCTTCTTGTCCCTAACTTCGCCAAAGGAGGGCAGGGAGAGAGAGTGCATGAGAGAGATGAAAGAAGAAGAGGTATAGAGGAGGAAGAATATAGAGGAAAAAAGGAAAGAAAGAGAATCAGGGAGGTAGATATCTACAGATTTGGGGTCTTCGAGAGGTTAAAAAAAAAACAGATGTAGCTAGAAAGAGGGATCAAGAGAGCAAGAGATAGACAAAGTTCTCAGGATTTCATCAGAGGAACATCCTTGTACTCACAAGGCTCAAGAGCATGAGTAGATTTCAAGTGCGTTGAACTGGAACTGATTAGAAATAAAGAAGCTGGAATGTAGAGAGGAAATCGCCCAAGCTGTTAGTTGCTAAATATTCACTTTTCTGGACTTCCCCAGGAATAATTGGGCATTCCATTCTCTGGGCTCCCATGCTGTTTGTGATGGTGTTAGTAAGGTGAAGATAGGCTTCTGGCATAGTTTACATCACAGTGAAGTGTGTATTTATTTAGATGCTGGACTGAGAAGCTTTGAAGCAGACACTGTATTCAATTCATGTGTGCCTATATTAGTGTTTAACAAATGTCTGCTGGAAGAGAGGAAAGAAAAGTAGGTGTAAAGGAGGGGAAGGAAGAAGGAATAATGGGCAGAGAAGCATTCAGGAAACAATTCCTCCTTATCTCAATCGGCATGAGTCAAACAGTAATTTTGGTCTCATGTCAGCAATTTCATTCTTTCAAATCTAGTGTATATTTGCCCTGACAATTAGGAGAGATTGAAAAAAAATGAGGCAAAGTGATTCATAGTCTATCTTTTCATTAGAAAGTTCAAAAATGAGCAGCAGCCAATATTGTGAATAACTAGAACTCAAGGGCTGATATTATGAACTTAATAAGCCATTATCTCTAAAATAATTATTCTGAATGAGCTTTTAAAAATGGTTTTATGCCTTTTATGTCCCCATCTGTCCCATGCCAAAATACCCTCCACTCTTAACATTTTGTCTTTGAAATCTGAACTATAATGTTCAGAAAAATTACAGTGATTTTTCAACCTCCTAAAAAATAGAAAATGCAGTATTAATCGTAGTGATATCTTCTATGTTAGGCATGTTCTTTCTTTTTCAAACCAGTTTTTAAATCTTTCGTCTCCTTTAGTGATCACAATAAGGATCTGAGGTTGTTAGAGCGAGTACTCTCATCTCCAGCCTATTAATGTGCAAAGTGAGGAAAGGGAAGTAACAGCACACAGTAAGATAATGTCTGAGCAAAAGTGAAAACCTAGAGCTCTGACTCTGAGCTCAGCACTCACCCTGCAGCAATGCTTAGAGTAGAGTAATTCTAGAATGTTCTTTGAGTATGTCTTTTAAATCAAAAGGAGAAAATATTTCCCAATTTTTTACATATCAGTCATGTTCTTACATATGCTAATAGCCTTATTTTATAAGATAGTTTATTATTATTATTCTCACCCAGCTGTGGTTTACATGTTTCTTTTAGCAGCAAAATTTTATTATCAAAATCTCATGCCAGGCCAGGCGCGGTGACTCACGCCTGTAATCCCAGCCCTTTGGAAGTCTGAGGCAGGTGGATCACTTGAGGCCAGGAATTCAAGACCAGCCTGGCCTACAAGGTCAAACCCTGTCTCTACTAAAAATACAGAAATTAGCCAGGCATGGTGGCACACCTCTGTAATCCCAGCCACTTAGGTGGCTGAGGCAGGAGAATTGCTTGAACTCAGGAGACAGAGGTTGCAGCGAGCCGAGATTGCACCACTGTACTCCAGCCTGGGTAACAGAGTGAAATGCTGTCTAAAACATAAAAAAAAAAAAAAAAAAAGATCATGCCAAACCTCTCTATATAAAACAGATAAAAGTGTATCTGTACTGGAATTGGAACAAAGCTGAGAGACCCAGAAGAGGTTTGCTGATAAGGGACTGTTGGCCTCAATCTCAATACCAAATACATAGAGATCACCAGAACACCACTGAACATAAGAGACTGTTGGCCTCAATCTTAACACCAAATACACAGAGATCACCAGGACACCACTGAACACAGAATGAAGACCTCGTCATAGAGCATGTCTGACTTCTGGTGAAGCGAGAATACTGAAGACTTCACAATCCAACTGATTCCAATTTCAAATCTACCACTTGCTCTCTGAGTGCATTGAAGAGGTTATCAACATTTCTGAGCCTCAGTTCCCTCATCTGTAGAAATTGTAGTAACACCTGCCAGATGAGTTTGTTCTGAAGATTAACTCAGATGCCCCAAGTGAATCGTGAATCGCAGTGCTCAGCACGCAGTAGATACTCAGGCAATTTCCTTCTGGAAGACCCTTGCCAAACTTACATATGCTTTGCTGACAAACCAGATGTGTTCTTGTTGCCTCAGAGGGTATAAATCCCATTTGTTTTTTTAAGTCCAGCTCATGCCGTTAGCATTTGAGAGCTTGCACTACTCCCTCAAACATTTAACATTTTCTGTTCAGTTAGCACAGCATCGAGACTTCAAAAGCAGCTTGAAAATAAACTTGATTCCAAATATGATTTATAATGTCCCAAGAAACAGCTCATTTACCCAAGAAGGCTTCATCAATTATTGACAGATGTACCCTCTGAGGAGAAATAGCATTAATACTAGGAGACTGAGGGAAAAGCATGCCTCAACCCCATAAGCCAATTATCCAAGGTGATTTTGAGCATTAAATCCTTAGATATGCTTAATTTACTGTCCCTTGCCTATAAGCACAGGAAGAAGTATGAGAAGGGAAAAAAATGGTAACCAATTTAAGACTGCTTTTAAAAACAAAAAGAAGGCATAGCATCAAGGGGAAAATGTATTGGAGGGAATAAGTGGGTGTTGGACACATATGAGCTTATAGATTGAACATCCCTGGACTTCAAGCATTTTGCCCCCTCAGTGCAGAGCCAGCTACTACCCACCTGTTTTTTTTCAGTACAGAAATAATTGTGCTTACCTGTCTCTGGCCTAATAATTATAGCAGAGACTGATTTTATTCTCAGCATGTTTATTGTGGTCGGATTTTCTCAGTTTTCAGGGCAAAGGCCTTACTAAGGATGCTAGCATTATTTGCTTTAACATCAAGCTAGTGACAAGGCTATGCAACGTTTTGTAAAAAGCCAAGAGGCCAGTGTGAATCCAGCCCAGGGGGCTTGTAATCTGTGTTTGACATGCAAATATTAGAAAACCTTGGCAAGACTACAAGGTTTACATGCAAGGGAAGAAAGAAAATACATGAGGCCAATACATTTGAGAATGCTAATATATTTCTCTACAGTTTTCTCAAAAGGAGGAAAATAGGAGAGGGTTATTTTTCCTAAAATCCAAATTATAAGGCAAAAGGTGACCAATTATGACTACTTAGCTTTAATTTTCATGTACTGAGGAGTGTGTGTGTGAGTGTGTGCATGTGTGCATGCATGCCTACATACATTGCATGCATGTTTTGAATGAGTGTGCGTAGATGGAAATAAAATAAACTTGGGATAGTGAAGAGGTCTACCTATGTAAAAATTTTTGCATGATGAATTGAACATTTCTATATTCAGAAATAAACCCTGCCACACGAAACCTAAAAAATTACTACCCCGACTTAATGCAATCTATAAAGTACAGTTATAGAACATTCTAAAATTACCTGCTTATACCTCATGACCTGGCAGAATAATTATAGGCTAGATTCTTTCATCTTTTTTACACTCTTTTTATTGAGAACTAAGAGATTCACTGTATTGGGTTATTAAAAGCCTGACCTCTTATAGCATGCATTCTTATCCCATTATTAAGCTGTGTATATAAAATTCAGTAGGCCTTCACTTTTATAGCTTTAACTTCTAAATTTCAAATTCAAAATCAGCATTGAAGTGCCAGTTGGGTGCCAGGCACTATGCAAGTGCTTTTAAAGGTACTGCCACATTTGATCGACACCCAGATTCCTGGCCAGGAATATTCTGATTCCAATCTAGTAAGCATCCTACGACACCACAACCTTCTATCATTGCCTTGCCATCAAGTCTTGCAAGGATTTTAGACAGAGAATTGGAGGGGAGGAAATCAGCAAAGCCAGATCCATGTCTGGTCCACCACTGGCTTTTTACTAGAAGTGTACGCCTTTATTCTTTGGAGACTCCAATTTCTCTTAACATAAAACTGAAGCAAGAATACAGAATTCTTGATCAACTCACAAAAACATTATCAAGCGTCTTAAGTACATTCACCCCTCTGAGGTTTCCTCGATGTTCAAGGTTTGAATAAACCATGTAACCTAAATAAACCATCAAATAAATGGTGTATCCTTGAACAACAGATAAGCTTTTTATTTTTAGATGGGGTCCCTCTGTGTTGCCTAAGCTTGTCTCAAACTCCTGGCTTCAAGCAATCCCCCACTGTCCGCCTTGCTGGGATTAAGGGCATGAGCCATGGCACCCAGCTGAGATGAGTTTTAACAACTAGTTATTTTAACAACGTTGCAATTGCTCAAAATCTTGTGTTGGGGAGAGGAGCAGTCCAGGGCACAGGGAGTCCTCTTTGGAATTGCCATCATCCCGAGAATGCATTCTTAACATCCTCAAATGTTCATTCTTTAAGGGATGGAAAATTTTTATCCTTTGGAAGCACATTTGAAACATCCAGAAGTTATTCAGAGCCAAGGGTGATAAAGTGAGGGATCAAATGGAATATGACTTTCTATAAATATAAGAGGTGACTATAAAGTAAAGTAACTCATTATCTTGAATAACTTGTAAGCTAGCATTGAAGACAATCGCAAAAGAAAATTCCAGAATATTTTGGGTATCTGTACTCAAGATATTTTAGGTAACTGCACACACCAATTCACAAAGTTACTTCTTCACAATAGTTAGATAGCTAAAACAGAGAGGAAAATATAAGAATACATTTCTTCTGTTAAGTAGGTAAGAAAACTGATTGGAAAGGTAAGCCATTGGCCTGGGCCATGTGTGTGTTTGCATATGTGTGTGCATGCCACACACAAATGGGAACTGCCCTCGTTCGACCATCATTTTGAAAGTTCTGCTACTTACCTCGTCTAATTATCTGAGCACCTGAATCTTGAGCATAGCTGTCATACCTCCTTAGAGCATGTTTATATCTTCGCATTCTGGGAGGGATGTAATTTTCTTGACCTGAAAAAAAAAAAAAAAAAAAAGGAGTGATGTCACAAGCTCTGCTTTTAATTACAACTATGTACTCTCCAAAAGAGTAAAGCATGATTACATATACAAGGATACCTTGTTTTATTGTGCTTTATTTACTGCACTTTGTGAATACTGCATTTTTAATAAGTTGATGTTTGTGGCAACTCTGCATCCAGAAATTCTATTCAGGGACATTTTTTCGACATCATGTGCTCACTTCATGTCTCTGTGTCACATTTTGTCAATTCTAGCAATATTTCAAACTATTTAATTATTATTATACCTATTACTGTGATCTGTGATCAGTGATCTTTGATGTTATTATTGTAACTGTTTTGGGACACCACAAACCCCACCCATATAAAAGGATACATTTAATCAATAAATATTGTGTTTGTTCTGACTGTTCCACCAACCAAACATTCCCTGACTCTCTCCCACTCCTCAGGCCTTCTTATTTCCTGAGAAACAACAATGTTGAAACTAGGTCAATTAGTAACCCTACAATGACTTTGTCTTCAAGTGAAAGTAAGAGTTGCATGTCTCTCACTTGAAATCAAAGGTTAGAAATGAGTAAGTTGATTTGGGAAGACATGTCAAAAGCCAAGATAGGCTGAAAGCTAGACCTCTTGTGCCAGTTAGCCAAGTTGTGAATACAAGGGAAAAGTTCTTGAAGGAAGTTAAAAATGCTACTCCAGTGAACACACAAATGATTTTTTAAAAAATGCAAAACAGTCTTATTTCTGATATGGAGAAAGTTTGAGTGGTCTGGATAGAAGGTCAATCCAGCCACAACATTCCCTTAAGCCAAAGCTTAATCCAGAGCAACGCCCTAACTCTCTTCAATGCTATGAAGTCTGAGAGAGGTGAGGAAGCTGCAAAAGAAGAGTTTGAAGCTAGCAAAGGTTGGCTCATATGATTTAGGGAAAGAAGACATCTCTATAACAAAAAAAAATGAAGGTGAAGGAGCAGGTGCTGATGTAGAAGCTGCAGGAAGTTATCTAGAAGATCTAGCTAAGATAGCTGATGAAGGAAGCTACACTAAACAACAGATTTTCAGGATAGATGAAATTTCAGTATAGATGAAATAGCCTTACATTGGAAGAAGATACCATCTAGGACTTTCATAGCTAAAGAGGGGAAGTCGATGCTTGGCTTCAAAGCTTTAAGGGACAGGTTGACTCTCTTGTTAGGGCCTAATGCAGCTGGTAACTTTAGGTTAAAGCCAGTGTTCATTGATTATTCTGAAAATGCTAGGGCCCTTAAGAATTATGCTAAATCTTGAGCCAGGTGGTGTGGCACACCTATAATTGGGAGTCTGAGGTGAAAGGATTGCTTGAGCCCGAGAGTTTGAGTCCAGCCTGGCCATTACAGCAAGACCTCCATCTCAAAAAACAAACAAACATATTATGCTAACCTACCCTGCCTGTGTTCTGTAAGTGGAAGAACAAAGCTTAGATGACAGCACATCTGTTTAGAGCACAGTTTACTGACTATTTTAAGTCCATTGATTAGATCTGTTGATTAAAGGAAAGATTCTTTTCAGAATATTACTGCTCATTGACAATGCACGTGGTCAGCCAAGAGCTCTGATGGAGATGTACAAGAACATTAATGTTGTTTCCATGCCTAGTAGCACAATATTCATACTGCAGCCCATGAGTTCAGGAATAATTTTCACTTTCAATTCATATTATTTGAGAAACACATTTTGTAAGGCTATAGCTGCCATAGAGAGTGATTTCTCTGATGGATCTGGGCAAAGTAAATTGAAAACCTTCTGAAAAAGATTCACCATTCTAGATGCCATTAGGAACATTCATGATTTACTGGAGCCTGAAGATGTGACTGAATTGCTGCTGTAATTTCATGATAAAAACCTTAATTGATGAGGAGCTGCTTCTTATGAATGAGCAAAGAAAGTGGTTTCTTGAGATGGAATCTACTCCTTGTGAAGATGCTATGAACATTATTGAAATGACAACAATGTATTTGGACTATTGCACAAACTTAGTTGCTAAAGCAGTGGCAGGATTTGAAAGGATTCACTCCAACTTTGGAAGAAGATCTACTGTGGATAAATTGCTATCAAATAGCATCACATGGTACAAAGAAATATGTTATGAAAGGAAAGGTCAATAGATCAGCAAACTTCATTATTTTAAGAAATTGTCACAGCCATCCCAATTTTCAGAAATCATCACCCTGATCAGTCAACAGTCATCAGCAGCAAGGCGAGACCCTCCACCAGCAGAAGGATTACAACTCATTGAAGGCTCAAATGATTGTTTGCACTTTTTAGCAATAAAGTATTTTTAATTAAGGTATGTACTTTTTTGGCATAATGCTATTTCACACTTAATAGAGTATAGTACAGTATAAACATAACTTTTATATGCACTGGGAAATCAAAAAATGTGTGTGAGACTCACTTTATTGCAATATTCACTTTATTGCAGTGGTCTGGAATATAACCTCAGTATCTCCAAGGTATGCCTGTACAATAAACTATGCATTGAGTACAGTGTTTCTGATCCTGCCTTCCAAACATATGTAGTTCTTAGAAAATGGAATAAAGAAGATGAAGCACCTTCCAGTTTGTGTATGGGAATACCTTTTGCAAAGGTTTTCCTTGCCTCAAAAGCCAAAAATCATCATTATAATAATAACCATCCAGATAGTGTGATTTAATACAAAGGTATCATGAACTTAGAAAATTGTGAACTAATACTATACTCTAGTTATAGTATTAGTTGCTTCTCTTGGAGGTATGGGTTAGAAATTCTAAAACCAGTTTATGTTTATACTAAGGTTGAAAAAAATAAGTAAATATATTGTAGGCAGTGAGGACAAAGTTTCTTGAGCTTGGGAAAGAAATTACAAGTAAGGAAACAGGAAAGAGAAATAGTACCCTAATAGCAACAAGCACACCTATTGCTCAAACCTTGGTGGTTGATTCCAAGGCTGGGGCAAGGAAAATACAAGATGAGTCTGGAATATCTTATAATTCTGGAAAATAAAGAAGTGCTCAACAAAAGATGGGGAAATGCAGAAAGGGCACAGGAGCCTACCTGAAAGAGCTCACAATGGCCAAAAGTGAAGCAATTTGAGCAACAAAATCAATAATGATAGCATTGTGTCATAACCCATAGAATAAGGTAAATAAATATTCCTGAATCTATACTTTCATAAATAAATGATTGAGCAAATAAATAGAGGGGAGAAGACATAGTTCTTCCTTTAGATAAATTCCAATTAAGAAACATAGGAAAATGTTAGAAATATGAAATCACCTCTTAAAACACCACAAATTGTTTCAGGCAAGATCTGTGGCTAGCCCCTGTGTCCCAGAGCCCAAACTACCTGCATCCGTCAATGAAAGTGAAGCAGCTTCGCTTGTCTGGGGTAATACCTGAGGTTCATTGCCTCACACCAAGGAAATCAAGGACGCAGACACACAAAATGTGAGGTTAAGCTTGGAGGTTTAAGAGGCAAAAGAAAGAGAGAAATTCTCCTGTAGAGAGAGAGGGGCTTCCGGTCCATGGCGAAGTGCAGGAGTTTTTATAGATGAGCTTGAGGAGAAGGTATCTGATTTACATAGGGCATATTTACATAGAGAGATAGTTTGGATCAGGTGTGCTGTTTGCACAGCGTGCGAAGAATCTGGCTGCCCCACCCTACTCTTTTATTATGTAGATGGCTTCTCTACCTGGCTAGCACCATGTTGCATGCTTCTTTATTGCACACGTGGCAACAAAGAAAAGGGAAAATGGATTCTCCCTATTGAACATACCTCACTTCCAAGTGTCCCTTTTCTATTAGTGCAGCTGCCCGCATATACCTATGCAAGCTTTCAGCTTGCTTATCTATGTTTGCAGCTTGATTTTTCAGGCTGCTTTTTGTTAAAAAAGATGATTTAGGGGCTGCTTTTTATTAAAAGGAAATTCCACCGAGAACTCTCTTACCCTCACTATCTGCCTAAATAGCTTCTATATCAAAAGTCCTAGTGTTACCATCTTTATCACAATTATTTGAGTCAGCCTTGCTAGAAAACATGTTAAGAAATGACACCTCCCCTTGGCATGGAGGGTCTATTTCTTGCCGGACTGGTCACCCAGGCTGAGATTCTACACTCAGAAAATGAGTAACTTCTTATCAAGCTGCACATTCATTTTCGCAGAGAAAGAAAGTTGGCACCTATTTTTCCAGGTTCCATTATGCCTGTTCTGGTTATTATAATAATCGTGCTTCACATTTCTATAGTGTCTTCAGTTTATAAGTTTCATTCATATACTGTACCTGAGCTAACCCCCAAAACCACCCTTTGAGATTAGTTTATGAATCAGTCAACAAATACTAACTGAGAGCCTGACACATGTCAGGACCTCAGAGTGAAACAGTGAACAAGACCAATGTGATCTTTGCCCGCCTGGAGTTGATATTACGGGGGGAAGATAACCAGAAAAAGTAAACACATAAAATAAGTACAAGGTATAGAGTTATAAAGAAAAGGAAACAAGCATACCAGACCAGGAAATTTTTAAAAATCAAGCATAATGTGAGGAGACCTACTTTAGAGAGATGTGAATGGAAGCCTGTCTGAGGGGGTGGTGTTTGATGAGAAGCAGGCAGCCTTGCAAAGCTTGGGGTAGCACCTCCCATCAGAGCAATCAGCAAGTGCGAGGGCGGTGTGTGCGGGAGTGGGTGATGTCAGGGGAGAGTGAAAACAGCTTGGTACATTCTGGAAACTATCAGAAGGCATGTTCTTCTGGAGAATTGAGGAGGAGGGGGATGGGGGCCTGAAATAAGGGCACAGAGGAAAGCAGAAGTTGCTAGCAGCCTGTGGATGAAGGGTTTTACTCTAGTGCATTGGGAAGAGGTACTGTCGTCTTACAGGTGAATAAATTTAAAATCAGAGATGTTATATGATTTCTCAGATTTCTCAGAGTTCTTACATCTGGCAAGTTACATCTCTGTCCCAAGTCTTTCCATTCCCTCCTTCTTTCTACAAATATGTATGAAGCACAGGAGATCAAGACTAGCCTGGCCAACATGGTAAAACCCTGCCTCTACTAAAAATACAAAAAAAAATTAACTGGGCATGGTGGCGTGTGCTTGTAGTCCCAGCTACTAGGGAGGCTGAAGCAGGGGAATCACTTGAACTTGGGAGATGGAGGCTGCAGTGAGCCGAGATCGCATCACTGCACTCTGGCCTGGCAACAGAGTGAGACTCTGCCTCAAAAAAAAAAAAAAAAAAAAAAAAAGCCAAACACATTACGACTTGCTAGATAAGATACAACTTTAATCTCAACTAGGTTAAGTCTAGAGGTGAAGAATGATTGTGTGAACAAATGCAACAAAAAGTGATAAACATAGGGCTGGGCATGGTGGCTCACACCTGTAATCCCAGTGCTTTTGGAGACCTAAGCTGGAGGATTGCTTGAGACTAGGAGTTCGATCAAGACCAGCCTGGGCAACATAGTGAGACCCCATCTCTACAAAAAATTAAAAAATTAGTCAGGCATGGTGGCACATGCCTGTGGTCCCAGCTACTTAGAAGGTGGAGGCAGAAGGATTGCTTGACCCCAGGAGGCAGATAGAGGCTGCAGTGGACTATGATCATGCCACAGCATGCCAGCCTAGACAACAGAATGAGACCCTGTCTTTTAAAAAAAAATGACAAATGCTATATCCAAAAATTTATACAGAATATAGAGAAGACCTGGAGAAGGGAGAAGTCAGTTCTGTGTGGGTAAGACCAGTAGGGTCAGAGATTTAGCACTGAGCTCAATCTAGAAGATGAATGGCAAGGGAAAGGAGGATGGGGGAGGATAATCAAAGCAGATTGAGCAAAGATACAGACAATAAAACAGCCTGGGTGGCTTCTGGGAATACTGAGCTGTTTCAAGCATGGGGTGAGGGTGCAGAGATGGAAAGTGGAAAAATAGAAATGACCAAAGGCCAGATCGTGAGCCCCTGATATGGCAAAGTAAGAAGCTTGGACTTCATTTTCCAAGCAGTGGAAGCCAGGAAGCAGGGAGCAACATGACTGGATTCGTTTCAGATAGGTCGCATCAACAGAACTATAGAGTTGAATTGTGGTGGTGCCAAGTGATAGTGGTGGAAGCAGGGAGAGCAGTTAGATGACTTCCAAGGTCCATGCAAGCAAGAGGAAATAGAGGCCTGAAGTAAAACTCCGGGTGTGAGTTGGAGAGATAAGGGTAGATTGGAAAGAAGGTGCAGAACTTAGAACACTGGGTGTTTCGGGTGAGGGAGACAGGGAATTCAGGGTGATTTCTGGGTTTCTTTTGACTCTCAGTTCAATACTTCTAATATGAAAAGTTTTTCTACCTTATATAAAACAGCATCTTTTGTTTGTTGCAAACAGTTTAACATTTCTTAATTGCCAGCTAAGAAAAACTCACCTGAGTTTATTCGCATTTTAAAGAGAGTATAATACATTTCCCTCTGACAAACTATTATTTAGAAGACAATGAAACTTTTTTTTTCCTAAAATGATCAGCATAATATCCTCTACTGAGATGAAGAACAAATTTCTACATTGCCACTGGCAAGACCGATATCTAAACAGAAAAAGAAGGTAGATTTTAACAAACTGAATTTTCCTACAATTTAAGTTTCTATCAAAGTAAAAATATTAGCACTCTATTTAAGAAAATAGTTCTGTCTCTGGGATCCTTGGAAAATGTGAAATAATAATTCCTTTTATGTTCAGACCTGTCTTGACATGCCCTGCTACCATCATAAACACAGCACACATAAGTTCAATTTCTATTCTCAGTTGCTCAAGAAATAACGTCAAATTCAAGTCTAAGAACTGACTTGAAAGATATGATATTAACATTTCATACATGGTGTCTAAAAAAGCAAAACATTTTAAAATTATATTTAAAGATCTTTGAAAATAGCTTGGCTATAGAAGACAAATGTCCATGACATCTGTACACTAACTATTCCAAGGGCGTATGTAGGACAGCTTCCATATACATTCTACCATCTCCATGTTTCCCCTTTATTGCCCATTTTATTTTACCATCTTATTCTTTTTGAGAGGTGGAGAAAACTCTTAACCATGCTTAGGGGGAGAAATGGTCTGAGATTTTGATTATCTTAATGGTTTACATAGAATGTGTTATGTGCAGAAAACTGTCAAAGAAAATGAATTAGGACTTTGAATTGTCTTGTTGAAAGCATCCCCACTGATGGCCATCTGGAAGAACTGTGCCACCTTCTCTAAAGATTCAAGATATATGTTATTATCTCCATGGTAAAGATGAGAAAATGGAAAATTAGAGAAGCTTAGTAACTCACTCAACATCACACAGGCCACTAAGTGATGGAATTGGGATTCAAGCCCACATCTGTGACTCTAAAGCTGAGGAAAAGTGTAGGTAGCAATTCCTAATTCTAGTGAACCTCCTAACACTAACCATTTTGGGGAAAACTTACAAGTGTCCTCTAGATATTTTTTCGAATTCACATTTTCTTTTCATAAATGTGGAAAATAATTTTGATTTATATGCATGTATCCTTAATTGGCTATCTTGCTTATTTATTTCAGAATGCAAAGTTACAATTATTAAACTTCTGGGTGTTTTTGCAAATTGGCAAAGGAAACAGAAACAATGGACATAACACACTTCTTGCAATAGCCTGCAGTTAATGGAAAGGGCTCTCATAGCCCTAGTTAACTCAGCTGTAAACTCACATTCTTCCCATCTAACTCCAGAAGCAAATCTCTGGGTGAAAAAATGAGTTAATTTGCTCTCTGTGCTAAAGTGGACCAAAGAGAGGCAATTCTCTATTGAGTAAGCAGTGTTGGAGTCTGTTAATGACAATCTTGAAGTAGCCTAACATTATGGTCTCATATGATAAATTTCAGTTTTCTAAATCTAAATTGCAGACTACTAAAAATCAATTGCTTAAGGAGAAAGAGGCTGTCGACAGAAAGAATGACGGTGGGAAGGAGATGGTTGGCATATTTTAAAAATAGATTTTAATATAATCAATTATTTTCCAGAGATATGGGTGACTTAAACCAGATGAGTAATTTCCCCAGGCTGCTTGCTACCTCTGTAGACTTACTTTGTATATATCATCAATGTTACAGGCTATGGGATGTTCTAGGAATCTTGTCTGTATTATGGAAGTTACAGGATGTTTATGGATGTTATAACAGGTTACAGGACGGTATAGAAAGTCATAGAATGGGTGAAGAGTTAGGAGAATTGAATGTAAATGGATGGAATATAAAACCCAGGGAGGTAGATGTATTCTCACCAGTGGAAATACCAGGACAACATTTGGGGATCTCTCAGTAGTGGACATGGTATTAATTTCCTATTGTGCTACAACAAATTTCCACAAGCTTAGTGCCTTAAAACAATGCAAATGTATTATCTAACACAAGGGTGTCCAAACTTTTGGCTTCCCTGGGCCACATGGGAAGAATTGTCATAAAATACACTAAAACTAGTGAAAGCTGATGAGCTTTAAAAAAAAATACAAAAAAAAAACTCACAATGTTTTAACAAAGTTTACTAATTTGTGTTCAGCTGCATTCAAAGCCCTTCTGAGCCACATGCGGCCAGCACCACCGATTGGACAAGCTTGATCTAACAGTTTCTAAAGGTCAAAAGTCTGGGTGGCTGGGCTAGATACTCTGCTTAGGGTCATCTCACAATGCCAAACTGAAGGTTTTGGTAGGGGTGTATTCCTTACTCCAGGTGCTGGGGAGAGTCTGCCTCCAAGCTCATTCAGGTTGTTGGGCAAACAGTTCCTTGCGGTTGTGGAACTAAGGTCCCCGTCCTTGCTACTGTAAACCAGGAGGCAGTTCTGGCTCCTACAGCTGCCCTGGTTCTTTTTTATTTTTATTTTTTAATAAATTCCAGGTGTCCAGTGCAGTTTTGATACATGGATATATTACACAGTGGGGAACTCTGGACCTGTATCCTTTCTTATGCTTTCCATGTGGCCCCCTTCAGCAATGGCAGTGGATTCCCTTTCCTGCTTTAAACTTTTCTGACTTCTCCCACATCTCTCTGACTCCAACAAGTTCTCCAGAAAGTTCTCCACTTTTCAGGGCTCATGTGATTTGATTGGACCAACCCAGATAATCCAGGATAACCTCTCTATCTTAAAGTCTTCACCTTAATAACATCTGCAAAGTCCCTTTCGCCAAATAACATAACAGCAATGTGCCACCAGAGGACAAAGTTCATGGGGCCGTAAATCCTGCCTACCACCAGCACCGTTGTTTCCCTGGGGACCTGGCCCACAAATGCTTTATTAAAGAATGTGGCAGCTAAAACCACCAGGCTACCTTCCAAACGTTGTGAGTGTGAGGATAATCCCCTGCAGAAGTGCCTTTCTGTTTAGAAAGGAACAAAATCAACTGGGTTCATACAAAACTTTTTTGATCTCTGACAGACACAGAGTACTTTAAAATAATGTACAAGTCAACAGAAAAGAGATCTGTCTTATTACTTTTTTTAATGTCATCAGAAAATATTGAGGATAGGTTTAGTAATAATAATAGCTATCCATTTCTTATTTGTACCAGATATTACATAGTCTGGAATATAGGCTGTTGTGACCATATATATATATATATATATATATATATATATATATATATATATGACTACATAAACTGAACTTACACATATGCACGATGTGGTAGAATAAGATTGTGCATGACATGGTTCATTGCTGCGACAGAATTGTTGACTAGTGCAATCACCAACCACTGTCACAAATCTCAAATCTGCTTTGACATCATGGTCTGCAAATTATAGTCTATCAGCAGACACAGCTCACTGGTCTCTGCTCAGTCATTTTCTTATGCAATAAAATCATTTCCCAGCAAGTTTACAATTGCGTGAGAGTCAGTTCATAGTGAGAACCATCCAGCACAGTCATTAAACTCCAGGGGCTTATACAAAAAAACCCAGACGTTTTTTAATTCTGTGAGTTAATATTTAAAGGACCAAGGTGTTTAATATATTGATAAGTGTAGAAATTCTTTATTTCTAAGGGTATTTAACATTGATCTTAATGATAGTGATTTGTATTTTAATGAATCCTAATGACAATAATTCAAATAATTTAAAAATGTATTTATTTCCCTCTGGAAATAAATTCGCAGCATACTGAGTTAAAGCCAGTTGTCTGGCCGGGCACGGTGGCTCACACCTGTAATCCCAGCACTTTGGGAGGCCGAGATGGGCGGATCACCTGAGGTCGGGAATTCGAGACTAGCCTGACCAACATGGAGAAACCACGTCTGTACTAAAAATACAAAATTAGCCAGGCGTGGTGGCCCATGCCTGTAATCCCAGCTACTCAGGAGGCTGAGGCAGGAGAATTGCTTGAGCCTGGGAGGCAGATGTTTTGGTGAGCCGAGATCCCACCATTGCACTCCAGCCTGGGCAACAAGAGCAAAACTCTGTCTCAAAAACAAACAAACAAACAAACAAAAAACCAGTTGTCACTAAAAGAGCTCAGGTAGTATGTCCTATGTTTTATTTAGAAGTTACCACTAAATTTTGGAATAGCCATCATTCCTAAGGTAATTTTCTACCTTCCTTTAAGCTAAAGTATTAGCAAATGGAAAAGAGTAGCAAAAATAACTCATTCTGTATCTTTCTATGCTTTCCATTCTGTTCCTTGTGATTGGCAAGTGTTGTTGGAGACTAATACTCACTGAACTTTAGAATGATTCCGAATACCTGTAATTTGTATTAATATCCGCTGGAACATTTCCCTGTGTTACCTTTTTCTTAAAAATAAAATAATTGATTAGAAGATTTAGATACTATATTAACATAGATTTCATTCTGATTAAAATTAGGAAAAAATTGGGGTTTTATGATTTTTTAGCATGTCACACTCCAGTTATAAATATAAGACTGCGTATTTACTCATAAATCCAGATTTAAATATTAGATAAAGCATATTTTTATATCAAAAATAAAAGTTTTAATCACTTTAAATTTAAGTCACTTTACTAAGTAGAAAATGAATTCAGTAAAGATACTATCAGCTTTTATATGAGATCTGCCAAATGGAAGCTCTCTAATAGAAAACTGTGACCTTTAACATAGTTTAACATCTACAATGAAATCTTATTTTCATTCTCTGTTCTACATATTATTTCATGTATTGTTGGTCCCAGAAAATTTTTAATGACAATATTTTCATTGACAGCATCTTAATATAACCTATATTCAAATTCATCGAAAGTCATGTATCACTTGTACAGCCTCTTTCTTGCCATTATTTTCAAAATATTGCTTGCATCCGATCCTTTTCTGCACATACTCAAATTCATACTAGGGCTGTTATGGAAATAGCTAATTTACATTTAGATTAAAAATTTCCTTGAAGCCCATTTACACCCTGATGAGTTAAAGACAAAGTAACTCAAAGCTGATAGCTGGCTTTCATTAAATCCAAGTATTATTACATATTCAACTAGTCAAAATACAGGCTTCAGGTTCATGGCTTAGATCCTAAAATGTCAAATAGGGTGATTGTAGCTCAGCAGACATAGCCAAATTCAGATATACTCAATGTTACTGCAGATCTTGAGACACTATCCTTTAGATTGACTCACTTGTTTGCTTATTCTTTATGTTTCATCCAATACTCATTAGAAAATAGTTGTTTAAAATCAAATCAGTTAGCAGGCCATTTGCAGTCTAGCCAATTTTAATTAGACTTCAGGAAAGAATTTTCTTTCAAAATGACTGGGACACTTCAAGTTTAACTGGCTGCTTAGTTTGGTAGCTCTATTCTGTCTGCCTGTCTGCCTGTCCATCTACTAAACTTCAGGGAAGCATTAGAAAATTGGTGCCTAGTAGGAAAGGCTTTTTAAACACTTCCAGTAAGTAGAAGACCATTCACAGTGGTTATGTCATTCAGTTTGTCTGCCATGCCCAACTTTTCCTTAAACTTTCCTACACATGACCCCTACCCAAAGGTGAAGGGCTAAGAGGCTGGATTAGGAGTACGTGACCCTGCCCCCAACCTGGCCACAGGCATTAACAAAAGATGAACAGCTCTTCATACACCTGTCCATCTGTTCAACTATCTGTTACCAATTGGATTCCCTGTCTTGAGAATTTGAACTTCTATCCACATAGATTGTGTCAGGAAAAGCTGAAAGGCCTTGTAGAACTGAGGCAAGCCACACTGATAAGAAGCAAGGTTATGGATCAGCAGATTAAACCATCCAGCAAAGAAGATAAAGAATACGTCTTTCAGGAAATGACAGAGTTGAAAGAACATATTGGACAAAGGAAACACAGTGAAGGAGAAATAAGGACAGAAGGAGACAGAAAGACCCACAGAGAGATTCAGAGAATGATGACATCAGAGACAGAGAACTTGTGAGTTCCCAATGGCATTCCATCTCCTGCGCCACTTCTCATGAAGCTTGGCCTTATTTGCTACCCTCATTTTGATGACATAACCTTACCTATTCTCCACATACCTCTTTTTCACTTCAACTGATGTGAATAGCTGTTCCTTTTAAGCAAACTGATTAAAACACGATGTTCAAGGATGCCTTCAAAATAAAGCAATAGATCAACTGCTTTGTATCTGTTTTTAACAGAATAAAAGAACAAAAACAATGTGTTTTCTAGAAGAGGAACAATTTCTGGACATCTTGACATGGAAAGATTCTTTTTTTTTTTTTTTGAGACGGAGTCTTGCTCAGTCGCCCAGGCTGGAGTGCAGTGGCGCCATCTCGGCTCACTGCAAGCTCCGCCTCCCGGGTTCACGCCATTCTCCTGCCTTCCCGAGTAGCTGGGAGTACAGGTGCCCTGCACCATGCCTGGCTAATTTTTTTATTTTTTTAGTAGAGATGGGGTTTCACCATGTTAGCCAGGATGGTCTCGATCTCCTGACCTCACGATCCGCCTGCCTCGGCCTCCCAAAGTGCTGGGATTACAGGCGTGAGCCACCATGCCCGGCCGGAAAGATTCTTTAACCTAACACCCAACATAATGTGCTCTTAAAAAACAAAGAAAAACCCTATGTCTGCTTTCTTCTTGTGAAAAAGTAATACATCCCTAACAGTTTTCACCCTTTAGAGTTTCTAAAGAACCCAAGGAAAAGTGACATGTTCGTATTCTAAAAGATGAAAAGGACTTAAAGACAGATGGTTTTGAAATTGGTTCTGTTCTCAGCATTTCACTCTGGTATTTATTTAAAAGGCGTTTCAGCCTACTTCTTCTGTATTCATCTTGCTCCATATACCTAACCACTCCTTTCTACCCAATTCACAGGCTGATGATCCTGACACTGTCTCTCTGTCTCCAGCTCTCTTCTTTATGACCTTTACCACAAAGAATACTTCCCTTTTTCCATATATAAACTAAGCTCCCAGTTTCCTGAAATATAGGCAACACTACAAAAGACATTGCAGCATATCTGGAGTACTGAAACAGGAAAAGTTCCCTTGCCCCCTTCACAGGGTGTGCGATGGGGGTGTGGCTAGCTTCTTCAGTACCCCACTGCTCAAACTTTTAGGGGAGCACACAGGCAGGCAGGCTGTGGGGCTCTGACCCCACAGCAGTGTCTGGGAGTGAATGTTTACAGCTGAAGCTCCAGTGGGCATATGTTACAAGGTGCTCTTTTAGTTTAGCCATCCCGCTGCTTGTGTTAGTAAGCTCAATTAGACCCACGCGTTGTCGTAAGGACGGAGGGCTTTCTGTATCCTGGGGTTCTTGCCTTGGTGTACCGGAAGAATCGGATCACACATGGGCTTTGAGAATAAATGCAAGCTTTTATTGAATGGAAGTAGCACTTAGCAGATGAGAGAGCCAGAAGGGAGATGGTTTTCCCCTGGAGTCAGGCTGCACAGTAGCCTGGGCTCTCCTCCAACTGCCCCTGCCAAACTCTGCCTCATTCCGCTGGTCGATGGCCTGATGGCCTGTTGGCGTGCCAGTGTCTGTCATGTGCTCTTCCACCAGCATGCTCCCCTCTACATCCTCTCAACGTCCAGCCATTTGTGTCTTCTTCCACCAAGGTGTTCCTCTCAACGTCCAGCTGCTTGTGTGTCTGCCTGCTAGTCGCGGTTTTTATAGGCACAGGATGGGGGCGTGGCGGGCCAGGGCGGTGCTGGGAAATGCAGCATTTGGGCGTGAAGGTAGAAGTGCCTGTCCTCACCTAGGTCCGTGGGCACAGGCCCAGGGGTGCAGCCCTCGCCGGGGCCCTGCCCTTCTCCTCCCAGCACTTCCTTGCTCCCCTTCCATATCACTACTGCCAGAGTATAAGAAGTTAAACACCAACTAAGATAAAAAGAAAGACTATGGCTCCTTTCTGCAGAAGAACTTTGATCAAATGTTTGTATTACAAATAAGGAGGTATTTATTTGAGGCTAAAATGACCTAATTCTAAAATAAATAAAATAAAGCAAACATAAGAGAAATTTTAAATAAAAACCTTTACTCAGCAATTCTACCTTTGTTTAGTCAAGAGAAGTAAAATGCATGTCCATACAACTATTTGTGCAAGAATATTTATAAAAGCTTTGTTCATAATAGCACCATACTGGAGACAGTCTAAAAGTGCATTAGTAACAGAATACATAAACAGATTGCAGTATATCCACATAGTGAATATGTTCAACAATAAAAAGGAATGAAGTACTGATATATGCAATAGAATATATGAATATCCATAATGTTATGCTGAAATAAAGAAACCAAACACAAAAGAGTGCGTACTATATGATTCCATTTATATGAAATTCTAGAGAAAAGCAAAACTAATCTATGGTAACAGAAAGCAGACCAGCAGTTGACTGAGGCCAGGGATGGGGGTGATGGGGGAGATGACTACAAAGAGGCATGAGGCAATTTCTTGGGGTCATGGAAAGGTCTTGATTGAGATAGTGTTCCCATAGTCATATACATTTGCAGAATGTCATCAAAATGAACCTTTTAAATAGGTACATTTTACTGCACGTTAATTATACATTAAAGTCGACTTAAAAGCAAAACATCCTAAAGCTGGGCCTTTCCCAATTCTTATTACTGTAAAGACATAATTGACTTTAAGTGACATTGTTATTTACATAAATGTTAAAAATATTTTTATTTAACATAGATTCTTCATGCAGATACATTAATTTTCTTAGTCATAATTTAATGGAATTTAATAATTCATGATATTCATATGTCAGAGTGGTACAGTCATGTGTTACTTAATGATGGGGATACATTCTGAAAAATGTGTTATAGGTGATTTCATATTTGTGTAAACATCATAGAGTGAACTTACATAAACCTAGAGGGTATAGCCTGCTACATACCTAGGCTATAGCATAGCCTATTGCTCCTAGACTACAAACTGGTACAGCATGTTACTGTACTGAATACTGCAGGCAGTTAAAATGATGAAAAGTATTTGTGTATCTAAACCTATTTAACATAGAAAAGTTACAATAAAAGTACTGGTATAAAAGATTTAAAATGGTACGCCTGTTCAGGGCACTTACCATAAATTAAGCTTGCAGGACTGTAAGTTACTCTGGGTGAGTCAGTGAGTGAGTGGTGAGTGAATGTGAAAGCCTAGAACTTTACTACACACTACTGTGGACTTTATAAACACTGTACACTTAGGCTACATTAAATTTATAAAAAGCTACTTTCTTCCTTCAATAATTAATTAGTCTTAGCTTGCTGTAACTTTTTAAATTTATAAGTTTGTTAGTTTTTTAACTTTTTGACTCATTTGTGATAACACTTAGCTTGAAACACAAACACATTGCACAACTATACAAAAATGTTTTCTTTCTTTATAGCCTTATTCTATAAGCTTTCTTCTGTTTTTATTTTTTTTTACTTGTTAAGCATTTTTGTTTAAAAAATAAGATACAAACACATTACCCTGGGCCTACACAGGTTCAAGATCATCAATATTACTGTCTTCCACATCCACATCTTGTCCCACTGGAAAGTCTGAAGTGCAGTAACATACATGGGGCTGTCACCTCCTATTATAACAATGCCTTCTTCTGGAATACTTTTTGAAGGACCTGCCTGTGGCTGTTTTATTGTTAACTTTATTTTTATAAGTAGAATGAGTACACTCTAAGGTGACAATAAAAAGTACAGTGTAGTAAATACATAAACCAGTAACATAGTCATTTATTATCATTATCAAGTATTATGTACTGTGCATAATTTTATGTACTATACTGTTATATGACTGACAGCACAATAGGCTTGTTTACACCAGCATCACCACAAACACATGGGTAATGTATTGCACTATGAGGTTAAGACAGCTATGATGTCAGCAGGAATTCTTCTGCTCCATTATTATAATCTACGAGTATTATAATCTACGAGACCAACATCACATGTGCAGTCTGTCATTGACCAAAACATTATTACAGAGCTCATGACTGTCTTAGGAAAACATGTACTAACCATCCACATCTTTGTTTTTTTTTTTAAGAGTTGGGAGTCTCACTATGTTGCCCAGGCTGGTCTTGAACACCTGGCCTCAAGCAATCCCCCGGTTTCAACCTCCCAAAGCATTGGGATTAAAGGCATGAGCCACTGCACCTGGCCGTATCCTCATCTTTATAGAACAAAAAGTCCATCATTCTTACATAGTACTCAGGCAAGAAGGATAAAAGTTTGGTTGACTTTAAAGAGAAAATAAGATATAACTCACTAGTCATTTTTCTTCTCTAAAATAAGCATTTTTGTATGTGTACATGTACTTTAGAGTACATTTTGTTTATGATTAACTTCTCCAGGTGCTAAGTCTAGTTATCCCTATTTGAAAACACCAAGTCATTTTCAAAAAGTAAAGAACTTCTGTCCCTGTAAGCCATATCAGGCCATCCCCTCTACCTGACAAAATCACTGATGTACCTACTGGTGGAAGCAATTCCATCTTCCATCAGGAGACTATTTCCTGACTTCCAGGAAACCTCCAAGAAACTGTCATTGCCCTGTGGCTATGATAAATTAGTCTGATTATCACATGAAGGAGACAGGTGTTAACTTTGGATCCCCAACCCCACCCACCCACTCATGCCACCTAGAACATCGATGAATCCACTAAGACAAAAATGCCCCAAAGTGGCTCCAGTGAAAGAAGCTCTAGGAACAACAACAAAAAAAAGATTTATAGCAAATAAGCTGCATTCTTCCAGGTCAAAAGCTTTGGGACTTGTTGTCTATTCTGACGGACATTTTCTACCCTTCTTATAATCTCATATGGCACAACATTTTTTTAAGTTTTTTTCTTTTTTTTTTTAAATTTTACTTTAAGTTTCAGGACACATGTGCAGAATATGCAGGTTTGTTACACAGGTATACATGTGTCATGGTAGTTTGCTGCGCCTATTGACTCATCCTCTAATTTCCTTCCCCTCACCCCACAACACCCAACTGGCCCTGGTGTGTGTTGTTCCCCTCCTTGTGTCCACATGTTCTCATTGTAAGACTCCCACTTATAAGTGAGAACATGTGGTGCTTGGTCTTCTGTTCCTGTGTTAGTTTGGTGAGGATGATGGCTTCCAGCTTCATGCATGTCGCTGCAAAAGACATGATCTCGTTCATTTTTATGGCTGCATAGTAATCCATGGTGTATATGTACCATATTTTCTTTATGCAGTCTATCATTGATGGGCATTTGGGTTGGTTCCATTACTTTGCTATTGTAAATAGTGCTGCAATAAATATATGTGTGCATGTGTCTTTATAGTTGAATGATTTACATTCCTTTGGGTATGCACTCGGTAATGAAATTGCTGGGTCAAATGGTATTTCTGGTTCTGGATCCTTGAGGAATCACCACACTGTCTTCCACAATGGTTGAACTAATTTACATTCCCACCAACAGTATAAAAGCACTCCTATTTCTCCACAGCCTTGCCAGCACCTGTTGTTTCTTGACTTGTTAATAATCACCATTCTGAGTGGCATGAGATGGTATCTCATTGTGGTTTTGATTTGCATTTCTTTAATGATCAGTGATGTTGAGCTTTTTTTCATGTGTTTGTGGGCCCCATAAATGTCTTCTTTTGAGAAGTGTCTGTTTATATCCTTTGTCCACTTTTTGATGGGGTTGTTTGTTTTTTCTTGTAAATTTGTTTAAGTTCCTTGTAAATTCTTGATATTAGACCTTTGTCAGATAGGTAAATTGCAAAAATGTTCTCCCATTCTGTAGGTTGCCTGTTCACTCTGATGCTAGTTTCTTTTGCTGTGCAGAAGCTCTTTAGTTTAATAGGATCCCATTTGTCAATTTTGGCTTTTGTTGCAATTTCTTTTGGCATTTTTGTCATGAATTCTTTGCCTATGCCTATGTCCTGAATGGCATTGCCTAGGTTTTCTTGTAGGGTTTTTATGGTTTTGGTTTTTACATTTAAGTCTTTAATCCATCTTGAGTCAATTTTTGTATAAGGTGTAAAGAAGGGGTCCAGTTTCAGTTTTCTGCAGCTGGCTAGTCAATTTTCCCAGCACCATTTATTGAATAGGAGATCGTTTCCCCATCTTGAGTCAATTTTTATATAAGATGTAAAGAAGGGGTCCTGTTTCAGTTTTCTGCGTACAGCTAGTCGGTTTTCCCAGCACCATTTATTAAATAGGGAATCCTTTCTGCATTGCTTGTTTTTGTCAGGTTTGTTGAAAATCAGATGGTTTTAGATGTGTGGTGTTATTTCTGAGGTCTCTGTTCTGCTCCATTGGTCTATATATCTGTTTTGGTACCAGTACCATCCTGTTTTGGTTACTGCAGCCTTGTAGTATACTCTGAAGTCAGTTAGCGTGATGTCTCTAGCTTTGTTCTTTTTGCTTAGGATTGTCTTGGCTCTACAGGGTCTTCTTTGATTCCACATGAAATTTAAAGTAGTTTTTTCTAATTCTGTGAAGAATGTCAATGGTAGTTTGATGAGAATAGCATTGAATCTGTAAAGTACTTTGGACCATATAGCCATTTTCACGATATTGATTCTTCCTATCCATGAGGATGGAATGTTTTTCCATTTCTTTATGTCCTTTCTTATTTCCTTAAGCAGTGGTTTGTAGTTCTACTTGAAGAGCTCCTTCAAATCCATTGTTTGCTGTATTCCTAGGTATTTTATTCTCTTTGTAGCAATTGTGAATGGGAGTTAATTCATGATTTGGCTCTCTGCTTGTTTATTGTGGGTGTAAAGGAATGCTTGTAATTTTTGCACATTGATTTTGTATCCTGAGACTTTGCTGAAGTTGTTTATCAGCTTAAGGAGTTTTTGGGCTGAGACAGTGGGGTTTCCTAAATATAGAATCATGTCATGTGCAAACAGACAATTTGACATCCTCTCTTCCTATTTGAATACCTTTATTTATTTCTCTTGCCTGATTACTCTGGCCAAAACTTCCAATACTATCTTGAATGGGAGTGGTGAGAGAGGGCATGCTTGTCTTGTACTGGTTTTCAAAGGGAATGCTTCAAGCTCTTGCCCATTTAATATGTTATTGGCTGTGGGTTTGTCATAAAGAGCTCTTATTGTTTTGAGACATGTTCCATCAATATCTAGTTTATTGGGAGTTTTTAGCATGAAGGGATGTTGAATTTTATCATAGGCCTTTTCTGCATTTACTGAGATAATCATGTGGGTTTTGTCTTTGGTTCTGTTTATGCGATGGATTATGTTTATTGATTTGCATATGTTGAACCAGGCTTGCATCCCAGAGATGAAGCCGACTTGATCATGGTGGGTAAGTTTTTTGATTCAGTTTGCCAGTATTTTATTGAGGATTTTCACATCAATATTCATCAGGGATATTGGCCTGAAGTTTTCGTTTTCTGTTGTGTCTCTGCCAGGTTTTGTTATCAGGAAGATGCTGGCTTCATTAAATGAGTTAGGGAGGAGTCCCTCCTTTTCAATTCTTTGTAATAGTTTCAGAAGAAACGCTCCTCTTTGTACCTCTGGTAGAATTTGGCTGTGAATCTGTGGTCCTGAGCTTTTTTTTTTTTTTTTTTTTTTTTTGGTTTGTAGGCTATTAATTACTGCCTCAATTTCAGAACTTGTTATTGATCTATTCAGGGATTTGACTTCTTCCTGGTTTAGCCTTAGGAGGGTGTATGTGTCCAGAAATTTATCCATTTCTTCTAGATTTTCTAGTTTATTTGCATAGAAGTATTTATAGTATTCTCTGATGGTAGTTTATATTTCTTTGGGATCAGTGGTGATATCCCCTTTATCATTTTTTATTGTGTATATTTGATTCTTTTCTCTTTTCTTCTTTATTGGTCTAGCTAGTGGTCTGTCTATTTTGTTAATTTTTTCAAAAAACCAGCTCCTGGATTCATTGATTTTTTTGAACGGTTTTTCATGTCTCTATCTCCTTCAATTCTGCTCTGATGTTAGTTATTTCTTGTCTTCTACTAGTTTTTGGATCAGTTTGCTCTTGCCTCTCTAGCTCTTTTAATTGTGATGCTAGGGTGTCGATTTGAGATCTTTATAGCTTTCTGATTTGGTCATTTAGTGCTATAAATTTCCCTCCTAACACTGCTTTAGCTTATCCCAGAGATTCTGGTACATTGTGTCTTTGTTCACATTGGTTTCAAAGAACTTCTTGATTTCTGCCTTAATTTTATTATTTACCCAGGAATCATTCAGGAGCAGGTTGTTCAATTTCCATGTAATTGTGGTTTTGAGGGAGTTTCTTAATTCTGAGTTCTAATTTGATTGCACTGTGGTCTGAGAGTTTGTTCGTTATGATTTTAGTTCTTTTGCATTTGCTGAGGAGTGTTTTACTTCCAATTATGTGGTCGATTTTATAATAAGTGCCATGTGGTGCTGAAAAGAACATATATTCTGGGCTGGGCACAGTGGCTCACGCCTGTAATCCCAGTACTTTGAGAGGCCGAGGTGGGCGGATCATGAGGTCAGGGGATCGAGACCATCCTGGCTAACACAGTGAAACCCCATCTCTAATAAAAATACAAAAATTAGCTGGGCTTGGTGACGGGTGCCTGTAGTCCCAGCTACTTGGGAGGCTGCGGCAGGAGAATGGCATGAACCCCGGAAGCAGAGCTTGCAGTAAGCTGAGATCATGCCACTGTACTCCAGCCTGGGCAACCGAGCAACAACAACAACAACAAAAATGTATATTCTGTTGATTTGGGGTGGAGAGTTCTGCAGATGTCTATTAGGTCCACTTGATCCAGAACTGAGTTCAAGTCCTGAATATTTTTGTTAATTTTCTGTCTTGTTGATCTGTCTAATATTGACAGTGGGATGTTAAAATCTCCCACTATTTTTGTATGGGAGTCTAAGTCTCTTTGTAGATCTCTAAGAACTTGTTTTATGAATCTGGGTGCTCCTGTATTGGGTGCATATATATATTTAGGATAGGTAGCTCTTCTTGTTGAATTGTTCCCTTTACCATTATGTAATGCCCTTCTCTGTCTTTTTTGATCTTTGTTGGTTTAAAGTCTGTTTTGTCAGAGGCTAGGATTGAAACCCCTACTTTTTTTTTTCTTTCCATTTGCTTGGTAAATATTCCTCCATTTCTTTATTTTTAGCCTATGCGTGTCTTTGCATGTGAAATGGGTCTTCTGAATACAGCACACTGATGGGTCTTGACTCTTTATCTAATTTGTCAGTTGGTGTCTTTTAATTGGGGGCATTTAGCCCATTTACATTTAAAACTAGTATTGTTATGTGTGAATTTGATCCTGTCCTCATGATGCTATCTGGTTATTTTGCACGCTAGTTGATGCAGTGTCATTGGTCTTTATATTTAGCTGTGTTTTTGTAGTGGCTGGTTTTTCCTTTCCATATTTAGGGCTTCCTTCAGGAGCTCTTGCAAGGCAGGCCTGGTGGTGATGAAATCCCTCAGCCTTTGCTTGTCTGGAAAGGATTTTACTTCTCCTTCATTTATGAAGCTTAGTTTGGCTCAATATGAAAATCTGGGTTGAAAATTCTTTTCTTTAAGAGTGTCGAATATTGTTCCCCAATCTCTTCTGGCTTGTATGGTTTCTGCCGACAGGTGTGCTGTTAGTCTGATGGACTTCCCTTTGTACATGACCTGGCCTTTCTCTCTGGCTGCCCTTAACATTTTTTTCTTAATTTTGACCTTGGAGAATCTGATGATTATGTGTCTTGGGGTTGATCTCCTCGTGGAGTATCTTAGTGGCATTCTCTGTATTTCCTGAATTTGCATGTTGGCCTGTCTTGCTAGGTTGGGGAAGTTCTCCCGGATAATAAAGATCCTGAAGTTTGTTTTCCAGCTTGTTTCCTTTCTCCCCATCTCCTTCAGGTACTTCAATCATTCGTAGTTTTGGTCTTTTTACATAGTCCCATATTTCTTGGAGGCTCTGTTCATTCCTTTTCATTCTTTTTTCTCTAATCTTGTCTGCATGCCTTATTTCAGCAAGGTGGTCTTCAAACTCTGATATCCTTTATTTTGCTTGTCCAATTCAGCTGTTGATACTTGTATATGCTTCATGAAGTTCTTGTGCTGTGTTTTTCAGCTCCTTCAGGTCATTTATGTTCCTCTCTAAACTTTTTATTCAGGTTAGCACCTCTAACCTTTCATCGAGGTTCTTAGCTTCTTTGCATTCTGTTAGAACATGCTCCTTTAGCTCAACAGAGTTTTTTATTACCCATTTTCTGAAGCCTACTTCTGTCAATTCGTCCAACTCATCCTCTGTCCAGTTCTGCACTCTTGCTGGAGAGGTGTTGCAATCATTTGGAGAAAAAGAGGCACTCTGGCCTTTTGGGTTTTCAACATTTTTTTCATTGATTCTTTCTCATCTTCATGAGTTTGTCTAGCTCCGATCTTTGAGGCTACTGACCCTTGGATGGGGATTTTATGGAAACTATTTTTTGTTGTTGATGCTCTTGTTTTTGCTTTCTGTTTGTTTGCTTTTATTGCAGTGGTAAGGTCCCTCTTCTGTAGGGCTGCTGCATTTTGCCAGGGTTCACTTCCGGCCCTATTCATCTGGTTCATGCCCATACCTGGAGGTGTCAGTCAGGGAGGCTGGAGAACAGCAAAGATGGGTGCCTGCTCCTTTTTCTGGGAATCTCTGACCTTGAGGAGCACCAATCTAATGCCAGTAGGTTTGCTCCTATACAGGGTGTCTGCCAACCTCTGTTGGAGGGTCTCACCCAGTTGGGTGGCGTGGGGAACAGGACCCACTTAACAAAGCACTTTGACTGTCCCTTGGTAGAGGGAGTGTGCTTCCCTGAGGGGAAACACACTTGTCTAGGCTGCCCAGATTCCTCAGAACCACCAGGAGGAAAGGCTAAGTCTGCTGATCCACAGAGACTGTGGCCACCCCTCCCCCTAGGGAGTCAGAGCCAGGGAGATCAGGGCTCTGGCCCTGAGCCTCTGGCTGGAGTTGTTGGAGTTGCTACAGGGAGGCCCTGCCCAGTGGGGAAGGATGGGTCAGAGTCAGGCCTGAAGAGGAGCTCTGGCCACAATCTGCCACAGCCAGTGTGTTGGGCTGTGGGGGACAACTCTTTGAACCAAGCCATCTAGCCTTCCTGGCTCCAGCAGGGGAAAAGCACAGGCTGGAGCTATAGAGATGGATGCCGCCCTTCTCCTGCCCAGGGAGCTTAGTGTGTTAGGCAGTTATGAGACCCAGTGCTGGCTGCTGCTCCTCCTTCAAGGAGCTCAAATGGCTTAGACAGCAGGAAGCCACAGCTGTGGTGCTGGTTGCCCCTCCCCCCACCCCAGGATTACAGCAGATTTAAGTAGATTCTAGCTGAGAGGCTGTTGAGAATCTGTGCAGCTCCAGGGTTTGGTCCCTAGGCCCGGGTGGTGAGGGTTCACAAGTGGGATCTTTCAATCCGTGGGTTGCACAGTTCTGTGGAAAAAGCATGTGTTCCCCAGCTGGGTAGCACGCTTACTCACTGCCTCCTTTGGATAGGGGGTTGGGGACTCCCCTGCCCCATGTGCCTCTCAGGTGGGCTGCTGTACCCCACTGCTCTTTCTTCCTCACCATGTATCATGCCAGCTGCCTAGTCAGTTCTGATGAGAGAACCTGAATACCTTGGTTGCCAGTGCAGAAGTCACATGCTTATTATGGTTCTTTTTGATGGGAGCCTCTGATTGTCACTGCTTCTAGTCGGCCATCTTGGCCCTGGCCCTTTTTTAGTGCTTTGAGTATATTATTAGTTGTTACCCCCAGGTAATCCACTGTCTCACTTGATCTATGATCTTTATAAAATGCTTCAAGAGCACAGCATAATAATTGAATTTTGACAGCTGATATAAAGAACACCCACGTGCCACCTGCAGCCAGGATATTGCTGGCTTCTGATAGGCTGGATTCACCTGTGAGACAGGGGGTTGGAATCTCAGTCATTGATGTACATCCACAAGAGTCCAGGGTGATAAAAGCTCTTGCAGTGAAATAATGTTGCCCACATTCGTTACAGTCCCTCACAGTTTTTACCTGTCCTAAGTGAATGTCTAACAATGTCCAATTCAGTGAGTGATGCTTCTCCAGCACAATAACTTCTCCTAAAATAAATGAAAACACGTGATTTGTGTACATAAAACATGAGGCACAAATATCTTCTGTCTTCTTAGGCCTGGTTGGTATAGTCAGAAGAACTTGGCGTTCCCCTCAAGCTGTCCCATTATTAAAGTATTAAAGAGATCCCTCACATATATGCTATATAAGGAAAGTCCTATGCAGAGACCAGACCCTCCTTTTGTTCATAATCCAGTCCCCATTGCTCACGTCACCTTTCCAGGGTCAGCTGAGAAAACCTTACCACCCTCATCAGCCATCCTGAGAATAGCACTGTTCCCACAAAGAATGATGTCTCTGTACACTCCCATAGTGTCTAATCTAAAGTAGCCAGAATACAGAAGAGGAAGCTTTCTTCCACATCTGTGACACACTTTGTCACTGGGCTCCCAAGGACTTACTGATCTGGGAGTCTCACAAACTGAGCTTCTAACTCTAGCTTCTTCTTTCTGTAATGTTTCAAAGCTAGTTCAAGGAAACTTGTCCTGGAAAGCTGCCATACATCCACATCATACTCTTATCCCCTTTCCCCCAACTGCTACCTGAGGTGATGTCTGAATGGTAGGAATGAGGGACATATATACACTGGTGTATTAGTCGGGGTTCTCTAGAGGGACAGAACTAATAGGATATATATCCTATTAGTTTATACATACATATATATGAGTTTATTAAGTGTATATATATATAAAGGAGAGTTTATTAAGTATATTAGTATATATATCTTATTAGTTTATGTATATACACACACATATACATACGCATATGGGAGTTTATTAAGTATTTACATATGTATATAAAGGGGAGTTTATTAAGTATATTAGTATATATATCTTAGTTTATATGTATACACACACATATATACACATATGTATATGGGAGTTTATTAAGTATTTAAATATATATATAGGGGAGTTTATTAAAGTATTAACTCATATGATCACAAGGTCCCACAATAGGCCCTCTGCAGGCTGAGGAGCAAGAAGAGCCAGTCCGAGTTCCAAAACTGAAGAATTGGGAGTCCAATATTCCAGGGCAGGAAGCATCCAGCACGGGAGAAAGATGTAGGCTGGGAGGCTAGGCCAGTCTCTCTTTTCACATGTTTTTTCCTGCTTATATTGTAGCCACACTGGAAGCTGATTAGATTGTGCCCACCCAGATTAAGGGTGGGTCTGCCTTTCCCAGCCCACTGACTCAAATATTAATCTCCTTTGGCAACACCCTCACAGACACACATGGGATCAAAAGTTTGTATCCTTCAATCCAATCAAGTTAACACTCAATATTAACCATCAGAAGTCCACCCCTCGCCAACTTGAACCCATACACATCTCGTGAGATCATAATCTTCAAATAAAGACAATAATAAGGTCATAATTATGCCTAACATAATACAACTATCCTTCCTTCATACAACCATAAATGTACCAGTCTCCAACTCAAATACTATTACATAAAGTTAACAATACTTGAATGCTGATGTGAAGTCAATAAATCTCATGTCACATGATAAAGTAAACAGGAAATAAAATGAAGATATTTTCTTAGTACAAATATATACATGCACAAACAGGTTTTTACAGAAGGAGGAAATAATCATGACAATTACAGTCCTCATTTCTGCAGCTGGTCATGTGATCATAGCTGATATTGATGACTACCGTCTTCTACTACCCATTCTGTATTCCCTTTGCCTTCAGCAAGCATCTTAGCAGGTTGTGGTTTTTTTCCTGGTGGAGTGGCCCAAACCTTTATTCCTAAAGGGTCTGGGCCATTTGTAGTCCTGGCTGGACTGATCTGTTGTAGTTTCCCATTGACCTGAATCACAGGGCATGGTAATACTAGGAGATGCCCTAATCGATCTCCTGTATTCCATGCATACTCTTCCTTACCTCCATTGTGGAGTAGTAGACTAATTTCATCTTGATAGTCTGGGTCAATCACCCCAGTCAACACTGTAACTCCCTTCTTAGCCTGTTAACTTAAAGGTAGGAGGAGCCCAAGTGTCCAGGTGGCAATCTTAACTCCCAGTTTAATGGAATTATTGTTGTGTCTCCTGGTGGCAGCATTCCTCTCTCTGGAACTAAGACCTCTATGCTGGCAGAACATAATGTCGTGGAAACAGGAAGGAAAAATTTTGCTCATGGGTCATTAGGGGTGATGGTGAGTGGTGTCACTTCTACTTCCACCCCTTGGTTCCTGGACCTGTAAATCCTGGCTATGGGAGAAACAGTACCATATATTGGACACTGATTCAGAGCATACATGGCCTTCTTAAGAACTTTGCCCCATCCCTGCAAAGTGTTGTCACCTAGTTGGCATTGTAATTGTGACTTCAAAAGGCCATTCCACCGTTCTATCAATCAGCTGCTTCAGGGTGATGGGGAACATGATAAGACCAGTGAACTCCATGAGCATGAGCCCACTGCCACACTTCTTTAGCTGTTAAGTGAGTGTCTTAGTCAGAGGCAATGCTGTGTGGAGTACCATGCCAGTGGATAAGACATTCCATGAGTCCATGGATGATAGTCTTGGCAGAAGCATTGCATGCAGAGTAGGCAAACCCATATCTGGAGTAAGTGTCTATTCCAGTGAGGACAAACCTCTGCCCTTTCCATGATGGAAGAGGTCCACTATAATCAACCTGCCACCAGGTAGCTGGCTGATCACCCTGAGGAATGGTGCCATATCGAGGGCTCAGTGTTGGCCTCAGCTGCTGGCAAATTGGGCACTCAGCAGTGGCCATAGCCAGCTCAGCCTTGGTGAATGGAAGTCCATGTTGCTGAGCTCATGCATAACCTCCATCCCTGCCATGATGACCACTTTGTTCATGGACCCATTGGGTGATGACAGGGGTGGCTGGGGAAAGAGGCTGAGTGGTGTCCACAGAACAGGTCATCCTATCCACTTGATTATTAAAATCCTCCTCTGCTGAGGTCACCCATTGGTGAGCACTCATATGGGATACAAATATCTTCACAGTTTTTGACCACTCAGAGAGGTCCATCCACATACCTCTTCCTCAAATTTTCTTGTCACCAATTTTCCAGTCATGCTTCTTCCAAGTCTCTGGCCATCCAGCCAAACCATTGGCTACAGCCCATGAATCAGTATATAATTGCACATCTGGCAATTTCTCCTTCATGTATAGTGCACAGCCAGCTGCACTGCTCAAAGTTCTGCCCACTGGGAAGATTTCCCTTCACTGCTGTCCTTCAGGGATGTCCTAGAAAGGGACTGTAGTGCTGCAGCTGTCCACTTTCAGGTGGTGCCTGCATATCATGCAGAACCGTCTGTGAGCCAGACCCTAGTCTTCTCTTCCTCCGTCTATTGATCATAGGAAACTCCCCATGAGGCCATCAGTGCAGGCTAGGGAAGAGAAGGCAGGATGGCAGGAGTGGAGGCCATGGGCATTTGAGCCACTTCCTCATGTAACTTACTTGTGCCTTCAGGACCTGCTTGAGCCCAATCACATATATACCACTTCCATTTGATGATGGAATGCTGCAGTGCATGATCCACTTTATGGCTAGATGGGTCAGAATGCACCCACTTCATGATAGGCAGTTCAGGTTGCATGGTGTCTTGTTGGCCCATAGTCAAACATTCAGTTTCCACCAAAGCCTAGTAACAGGCCAAGGGCTGTCTCTCAAAAGGAGAGTAGTTACCTGCAGAAGATGGCAGAGCCTTGCTCCAAAGTCCTAGAGGCCCGCACCGTGATTCACCTATGGGGGCCTACCAAAGGCTCAAAACAGCATCCCTATCTGCCACTGACACCTGGAACACCATTGGATGTGCTGGGTCATATGGTCCGAGCAGCAGAGCAGCTTGCGCAGCAGCCTGGACCTGTTGCAGACCCTTGCCCTGTTCTGGATCCCACTCAAAACTGGCAGCCTTTCCGATCACTCGATAAATGGGCTGGAGTAACACACCCAAATGAGGAATGTGTTGCCTCCAAAATCCAAATAGGCCCACTAGGCATTGTGCCTCTTTCTTGGTTGTAGGAGAGGCCAAATGCAGCAACTTATCCTTCACCTTAGAAGAAATATCTCAACAGGCCCCACACCACTGGACCCCTAGAAATTTTACTGAGGTAGAAGTTCCCTGAATTTTAGTCAGATTTATTTCTCATCCTCTGGCATGCAAATGTTTCACCAATAAGTCCAGTGTGTTTGCTCCTTCTTGCTCACTGGATCCAATCAGCATAATGTCATTAATGTAATGGACCAGTGTGATATCTTGCAGAAGCAAAAAGTGATCAAGTTCTCTCTAAATAAGATTATGACACAAAGCCAGAGAGTTGATACACCCCTGAGGTAGGACAACAAAGGTATATTGCTGGCCTTGCCAGCTGAAGGCAAATTGTTTCTGGTAGGCCATATGGACAGGAATGGAGAAAAAGGGATTTGCCAAGCCAATGGCTACATACCAGTTACCAGGAGAAGTGTTCATTTGCTCAAGCAATGAAACCACATCTGGTACAGCAGCTGCAATTGGAGTCACCACTTGGTTAAGCTTATGATAATCCACTGTCATTCTCCCAGACCCATCTGTCTTCTGCACAGGCCAAATGGGAGAGTTGAATGGGGATGTGGTGGGAATCACCACACCTGCATCTTTCAGGTCCGTGGTGGTGGCACTAATCTCTGCAGTCCCTCCAGGGATGCGACATTATTTTTGATTTACTATTTTTCTATGTACAGGCAGCTCTAATGACTTCCATTTGGCCTTTCCCACCATCATAGCCCTCACCCTACCAGTCAGGAAGCCAATGTGAGTGTTCTGCCAGCTGCTAAGTATGTCTATGCCAATTATGCATGCTGGCACTGGAGAAATAATCACAGGATAAGTCCAGGGACCCACTGGACCCACTGTAAGTCAGACCTGAGCTATAACTCCATTAATTACACGACCTCCATAAGCCCCTACTTTAACTGGAGGACCACAGTGGTGTTTTGGGTCCCCTGGAATCAAGATCAGCTGAGAGCCAGTGTCCAGTAGTCCCCACAATGTCTGATCATTTCCCTTTCCCAATGCATAGTTACCCTGGTAAAAGGCCGGAGGTCTCCTTGGAGAAGGATGGGAGAAAGATTCACTGCATAAGTTAATGGTAATGTAGTGGGGTCCTTCCTCAAGGGGATCTGGCCTCCCCTTCATTCATGGGGTTCTGGGTCTGTAAGCTGGCTCAAGTCTGGAAATTGATTGAGAGGCTGTGATTCTCTGTTTTTATAATTCAAATTAGTCTTTTGTCCATTTGACCTTTTCTGCTTGTATAAATTAAGTAGGAAGGTGGTAGGCTTCCTATCAATTTCACTTCTAGGAACACCATGATTAGTTAGCCAATGCCAGAGCTCTACATGAGTCAGACTATTCTGATTGCCGCTTTACCTCTGCTCTGCATTACAGTAGCTACACCCACCTTGCCTTCGACAGCTGAGTGCCACCACTTGGCTCCTGCAACCTCGGGATCCAATGATTCCCATTGTATTTAAATTTTGTAGTTGAGAGACTGCAGTTCCCACTGTTAGATATGACATACGGAGAAGAGCAATTACAGGGCTCTTCAAATATGCAGGACGTGCCCTCACAATTCTATTTCACAAGTCATTGGCCAAGGGCTTATCTTCTGAACCCTCCCAGCTGGGATGAGTAGGTCTAAAGTGACTAATCCACTTCACCATCCCAATCTCCCTAAGTTTTTGGATCCCTTCCTCTACTTTAAATCAAGGGAGATAAGGGATTTCCCACTCACTGACGATGGGCCATCTTTTAATCTATATTTCAGCTAACCAAGCAAATAAACTATTAGGACCTTTTTTTAACTCCCTGAGCTGCAACATTAAATGCAGAGTCCCTAATTAGTGGCCCAAAGCAAGAAATTCTGCCTGATCCAACTCTATGTTCCTTCCACCATTATCCCACACCCTTAATATCCATTCCCATGCCTGTTCTCCAGATTTCTTTTCACATAAATTAGAAAACTCAAGCAATTCTTTTTGAGTGTAGTGGACCTCCTCATGGGTCACACTCTCAACCTCATCTCCAGGGGTCTGCTGGGACTTTAGTCTAGTTATAGGTATAGGAGCAAACAGGGGTGTTGGGGGTGACTCCTGAGGAGAATCAACATTATCTTGCCTGGCAACTATCCCTGGGGAGGCCATCACTGTTGCCTCAGGCAACATAGGGTTTATCTACTCAGACAAAGGTGGAAAGGCTGATGGCAGCATGGGTTGGTGAGGGGACGTTGCCACTACTGGGGATGGGGAAGCTGTTTCCTCTGGCAAAAATGTTCAAACTCAGTGTCCCCAGCTTCATCAGGGTCCTCCCACACATCCCCAACCAAGTTTCAGGATCCCATTATTTTTCTAGTCAATGCCCTCACTTTAAGGGTAGACACCTGGTGAGGCTGTGCGTGCACATTTTGTTGCAGTTCAGCCACTGACATGATAAGAGCTTGTGTCTGTTTTTCTACAATTTCAGCTCTTTCTCTACAGGGGGTAAGACTCTCACTCAGGACTATCTCAGCAGATCTGAGGCTCAGTATCTGCTTCTGAAGCCAGGAGACAGAATCCCTGAGTTCATCATTTTCTTTCATCACTTTGTCCACTGAACTTAGGAGCAACCAACCAGCTTCATTATGTTCTTTGGTTCTCCACATATAGTCAAAGGTATTATGTATAGAGTCACTAAACTCCTTGCCTCTCATGAATGGTGTCAAATGCATTTATTTTGCATAACTCTCTAAGCAGTTCACACCAAGGACTATCAGTGTTCTCCATACTATTAGAAATAGTGTCTTTAGCATTTTTGGGTCAATCATATTAAGCAGCTAACTCCAGAAACCCCAAAACCAACAAAAGAACTCCATCCTTAATATTCTGTTTCTCTAGAACCACTCCTGGCACCAATATCTGTATTAGTCAGGGCTCCCTAGAGAGACAGAACTAATAGGAGATTAGTTAGATTTTATATATATATATATATATATGGGAGTTTATTAAGTACTAACTCACATGATCACAAGGTCCCACAATAGGCCCTCTGCAGGCTAAGGAACAAGGAGAGCCAGTCCAAGTTCCAGAACTGAAGAACTTGAAGTCTGATGTTTGAGGGCAGGAAGTATCCAACATGCGAGAAAGATGTAGGCTGGGAGGCTAGGCCAGTCTCTCTTTTCACATTTTTCTGCCTGCTGATATTCCAGCTGCACTGGAAGCTGATTAGATTGTGCCCATCCAGATTAAGGGTGGGTCTATGTTTCCCAGCCCACTGACTCAAATATTAATCTCCTTTGGCAACACCCTCAGAGACAGACACAGGATCAGTACTTTGTATCCTTCAGTCTAATCAAGTTGACACTCAGCATTAACCATCACAACTGGCAACATGCTCTATTATACCAACACTTAAGCAAATGGGTACAGAATCAAGGGAGGGACCCAATCTATTTTTTCTCAGTGTGTGATCTCTGACAACCTGCACAAAATACTTGAGTGCTAGTTGAAAATACATATTTTTCCTCTGATTCCACCCTAGACCACTATATCAATCATCTGGAGAGGTGAATTCTAAATCTTTAAACAAGATCATGAGATGATTTTTGCAGAGACCTAAGTCTGAAAATCATTGCTGGATAATCTGAGGAAAAACACTGGTACAATTCAAATTTTTTTGAGATAAGCAAAACATGGGTATTCTCATTCATCTCTGAGACCCTTCCTCAGCAAGTAGCCACTAAATCGTATTATTGACATTTGACTAAAATGGGGGTCATTCAAAGGATGCAGGCCCTAAAAAAATGAAGCCCAGGAGGAACGCCTTGTAGACATCCACAACTTTCACATGGATATTTCCTAACGAAAAAATGGAAACTGCTGGAAATGTTTTTAAATGATAATGAGGACACTGATATTTAATTTTTGCTTTTAAGGAGAAAATATAGAAATATCAGGAAACAATAAGATTTATTTGTAATCCTAGTTTTTAATTCACTATTGATATAAAATAAAAGATAGGTTATTTGTTTAGAAAGATAAGGAATATGTTTAATTTGCTTTGCTCTGTCAACCTAAATGGCAAACAGAATGAGGCTCTGTAGAAGAAAGTGATAATCTATGCATGCCATAGTAAACTATGTACATATTCAGGAAGGTAAAGGAAGACAAAGGTTTTTAAAGGAAAAATGAAGAGGATTATATAATGTTTTGAGATAATTATCCCTGACTACCTGGTTCAATAAGAAGGGTGATGCTAGTCCAAGGTTGGATAGGCAGTTGCAGGGCAGACTCTGCAGAAGTACTTTTTGTGTAAGGTTGTGATGGCCTTTGTGCAAGGTTGTGGTTCTTGTAGAGTCTTTTGTGATAGTTTTTGTTATGAGACATTTATGCATGAGAACCTCTCTCTTTGAGGCCTTCCCCAGCTCTATTTGTTAGGATTTTTTTTTTTAGCACAGTTGACTTCATTTTGATTCTTCTAACTTCCACAGCTTTTAGAAAATATTGTCATATTTCATGTGTTAAACATAAGAGTATAGTTTGTGTCAAGAATAATTATTGGCCAGGCACGGTGGCTCACGCCTGTAATGCCAGCACCTTGAGAGGCCGAGGCGGGCGGATCACAAGGTCAGCAGATCAAGACCATCCTGGCTAACACGGTGAAACCCCGTCTCTACTAAAAATACAAAAAATTACCCGGGAGTGGTGACGGGTGCCTGTAGTCCCAGCTACTCGGGAGGCTGAGGCAAGAGAATGGTGTGAACCCAGGAGGCAGAGCTCGCAGTGAGCCGAGATGGTGCCACTTCACTCCAGCCTCGGCGACAGAGCAAGACTCCGTCTCAAAAAAAAAAAAAAAAAAAAAAAAAAGAATAATTATCAATGCACTTACTGAATTTACCTTTAAATGTTTTAGGGATACCAGTATTTTGTCTCCATCTCTTTCAGTTAATAAATAACAAGCAAATATCTGAGGAACAAGAGAAGGGGAACTAGTAATTATTGAGTAGTTACTATATATCAGTTCTTGTATTAAGCAACATATAGGCTATGTCTGAGAAGAGAAGAAAGTGTGAAAAAGTGTTTTTTAACACACTAGAAAAAGTTATGTTTTCAGAATTTAGTAGATCTCATGGTGACAAGAAGCTACAGAAAGCACTAGTGTTCAGAAGAGCAAGTGAGGGTGCAGGACAGTTTATTGAATCCAATATTTGCCAACAGTAGCTAGCCTAACTCAGCGGTTCTCAAACCTGAGGGTGCATCATCAGAATCATCAGAGGAACTTATTAAAACATGATTATGTCCCCCCTCCCCCACTTTTGGAGTTCCTGGTTCTGTAGCTCTGAAGTGGGGCTAGAGAATTTGCACTACTAAGTTCCCAGGTAAAGCTGATGTTACTGTCTGGGGACCACAATCTGGGAACCACCGGTCTAACTAGTTTCATTTCCTCAGTTGTGGCTGATCCTGTAGCCTTGTCTCCATGTTGCAAGAGGTATACATGCAGATAATACCAGATGCATCCTGGAACTAACAGCCTAAAGTATTTCTAGTTCTGCAATTGTTTTTGATAAGGAGAAATAATTCTTATTTAAAGGTATATTTTGGTCAAGTTAGAAAATTTGGAGAAGAAAAGTGTCCTTACAACAACTGAAAATACTTTTCCAACATGAAATCTTAAATTTGCACCATGTTTTATAATCAAATGAAGTAGGGATCTTGCTTTGTTTATATTTCTCCTCTTGAAACTTGGCTACAGAAGATAAGCACAAGCATAAAATGGCTGACAACAAAATTTGCCTGTTTCTTAAATTTTTTACTTTTTTGTACACTTTTGAACAAAAGATCCACCTTAAATCTCAAATATATTTTTCCCTTGGCAGCCTTCAGCTGCAGTGCCTCTTGAAGAAATTGGCTTTATAACCCACAGAGAATTCTGCAGTATGCATCACCATCAAGCATCACAGTTCTCACACTTCTGAATACTTAAAGACCCCCAATTACTAATTTTCTTAAAGAGCTTTGTTGTTTTCTAGCAAAAAGCATATTATCATTTTACAAACGTAGAATTCAATCCAAACTTCATCTCAGCGTATTCCTTGCATCTTTTCTTTCTTGGAGCTGAATACTATTAAGACGACTGTTTATCCTTTGTAACTTTTGTTCTCTTCTCCCTATATTTTCAAGAATATTTCATAATATCAATAAACAAATCTGAGCCACAGAATGCACATCTTAATGGTGTCATTGGATAGTTGCCTCTCATGGTTTCTCTTATGGCCTTGATTTTATTTACACCATTAATCAACAAATATAGCAGATCTTAATACTCAGAGGATGTTATAAGAAAACGGGCAGCTATTTACACAAAGAAAAAAAATGCTGTGCCACGTTTTGAGAGTTGCAAACAGTAACAGAGAATAAAGGCTTGATTAAAAAACAAACACACACCTGCTTTGAGGTGTCATAACACAAGCATGGTAGTGCAGTGATATGGCTCAGATCGGATTGTGGAAGCATGGGGAAGTTGAAGAAGGAACTCACTGTTAGGAGAGAGGGTACTGTGTGGGAGGGGTAAAAGCAGCCCAACTAGGGATGTCTGAGCCAGCGTAGGGTAAGGAGGGTATCCTATGGGAGATTGCAGTGGCAAAGGGAGATTCATCACATCCAGGACTATTGATTAAGTGTTATATAAGGCTAATGAGAGTCAGGTTTCTCACTGTTGGAGAAGGCAGTTACAAATATGGAAAAGAAAAAACTAGAATAAACTTTGTGGCATTGGATTGAAATTTGAGGTATAGGTGCAAACTCATTGTTTTCAATATAGATAGTTATGGAAATATAGATGTTAATGTGAGGATAGATAGGCAGACAGACAGACAAATATTCCCTACCTCTGTCCACCAAAAGGGCTTCAGAGTAGTAAGACTGCAATGAGAACAACTGGTCTCCAAAACTTGGCTTCTAAATACCTTTCTCTGCTGGTCAGGACTCTCGGCATAATGAGTGATTTCAGGATTGGGCAAGGAGAAAGTACGTGAGCCTGGTGAATCTTATTGCTCCAGGAAGTAAGGAAGTGCTCAAAGAATTATAGAACTGTGTCAAAAGTACACATAATCCATCCTAAAGGGACTTCCACTGGATAAATTAGGGATAATTTGAACATTAAAATAATTACGGTAATACATATAATCTTGAATTTAAAAAGAAACCATGAGTCCATGCTGAATATATAAACAGATAAATAAAAGCTGTGATGAGGAACGGGAAATTTACATGGTTTTAAAGTACCTCTTCACAAAAACATTTATTAATTACAAAAAGGAAAATTATAACTTTACAGAAGTCCTGGCAGATACTTAATTCAAATGATCAAAATGAACACAACTAATAGGGCAAATTGAAATCATGAGCCACTAGATATGATGTGATGATAATAATACATTATTTCTGTGATATTCTCGGAAAAGATTCTTGATTATAATCATGAGGAAACAACAGACAAACCCAAATTTTAAAACATTCCACAAAAACACAAACTGAGGAACATTCCACAAAGGAATAATCTTCAAAAGTGACAAATAGTGGAAGACAGACCGCTAGGAACACCAATAATCCCTTCCCTTGAGTTGGGCAGAACCTACCCACTTTTAACAAACTTTTAATTACTTACTTTTAACCAGTAAGATATGGGAAAGTTGATGGGATATCAGTCCCATCATTACATTATGGCATATTTAAGATTCCCTCTTGCTAGCAGAGATGCTAGAAAGACTCCACCTCACTGTCCTTGAAGATGTGAATTGCCATAAAGTTAAGTGGTCATGTTGGAGAGGTTCACATGACAATGAACTGCAGGCAGCCTCTAGTAGCTGAGAGTGATCTCCAGCAAGAAACTGAAGCCTCAGTCTTACAGCATCAAAGAAATACATTTTGCCAACAACCTAAGGGAGCTTAGAAGCAGATCTTTCCCGTCAAGCCTCTGGTGAAACCGCAGCACTGGCTGACCTGTGGATTGCCTCCTGATGAAACTCTGAAGCAGAGAAGCAAGCTAAGACATGCCTAGACTCCTGATCCCCGGAAACAGATAATAAATATGTGTGTGTGTGTCCGTGTGTGTGTGTGTGTGTGTGTGTGTGTGTGTGTGTGTGTTTCAAGTTGGTAAGTTTGTGGTAATTTGTTATGCAGCATAGAAAGCTAACGTAGTCATGAAAGTCCATGAAAGGTTGAGAAATTACTCCACAGTAGAGGAGGCCAAAGAAACGTTACACGCCAGACTGGGTGGCTCACGCCTGTAATCCCAGCACTTTGGGAGGCCAAGGCGGGCAGATCACCTGAGGTCAGGAGTTCGAGACTAGCCTGGCCAACGTGGTGAAACCTCGTCTGTACTAAAAAAAAGTACAAAAATTAGCCCGGCATGGTGGTGGGTGCCTGTAATTCCAGCTACTCGGGAGGCTGAGGCAGGAGAATCGCTTGAACCTCGGACTTGCAGTGAGCTGAGATCGCGCCACTGCACTCCATCCTGGGCGACAGACCAAGACTCCATCTCAACCAAAAAAAAAAAGAAAGAAATGTTATAAACCAATGCAATATATGATTCTGAATGGGATCTTTGTGGATGTTATTGGGATAATAAGAAAAACTTGAACGTGGTCTGAGAATTAGAAGGTAGAAACAAATTAGTGTTATTTTCCTGATTTTGATGGCTGCTTTGCAATTATGTAGGAGACTATCCTTGTTATTAGGACATAAACACTAAAGTGTACAAAGGTGATGGGGCATCAGGTTGGCAACTTTCAAATGGTTCTGAAAAAAGTTATTTGTATTAGACTCACAATTATTTTGTAAATTTGAGAGATTGCTTCAAAAAAATATTCAAAAAACACATTAAAAAGCTGTTTCCACCCTTGTCTTCTCCATCTCAGTAAATGGCACCTTATTCATCCAGTTGCTCAGGTAAAAATCCTAGACCTCATCCTTGATTCCTCTATAACCTGATACTGTTCACTCAATTATAGAGTGATCCAAGTATCTATCACCACCTCCACCATTACCTCCTTTGTTCAAGCCAACATCTTCTTTGGCCCTCTTGTTTCAATAGACTCCTGGCTGCTCTCCCTGCTTCATGCTTGCCCTACAAGTGTCAATGCTCTACACAGTAGCCAGAGTGATGTTTGAAATTTTTAATTTAGAACCTGTGATTCTCCTGCTCAAAATCTCCAGCAGCCGCCACAACACACCTTTCATGGGCTACCAGCCCCTATATCATCTAGCCCTGTTCTCTGACTCATGTACCACCCTCCTTCATCATCCCCTTCAGACTCTACAGCCTTCTTGCTGTTTCTTGAGCTCCAGCATTTGCATTTTGTGTTCCCCCAGACAGCAATATGGCTTCCTCCTTCTCTTCAGTGCAGTCTCTGTCAAGTGCAACCTCCTTAGAAAACTCTTTTTTAACCAATATTTTTAAATGTTTAAAATGCACCCTCCCTCTCATCATAATATACATGTATCCCTCACTCCACCTTATTTTTCTTCATGGTACTTATTACCAACTGAGAGTAAAAATTTCCTCTTATTTTCTTGTTTTATTATCTGTCTCCTCTATGAGGGCAAGGCATGTTCACCTCCGAATCTCCAGGACTAGAATGCTGCATGTTGAATGAGTGAGCACGACAGTACTGACATTCAGTTTCTGAGGAGTCAGCTTACTCACTAACTATATATACTTGTCTACAAGCCTGGCAGTACTCATGACCAAAGTTCCTGCCTCAGAAAAATAAATTACTGCTGTGCAGAAATGCTAGGGTCATATAAAATAGGTAAAACCATAACCTTCTAATTATAAGAGTCCATTAAAATTAACCTTTGTCTATCCTAGAGAATGCCTTCTGCTTAACACTGATAACAGTGCTGTCTCATTGGCTTCGTGTGCCCATCAACTGTGTTTGTTGAGTACAGAGCCCTGAACAAAAAGAAAATCTCTGAAACAGGAGGAAGACAAAAAGACATGAAATTAAGTCAATAAATTAAGGGGACACAGAACTTGAGGAAAATTCATAGCTGATGGAGACAGAGAGAAAACAAACAAGGAAAGGAAACAGGACTAGAGAATGCTTTACATTCTTTTGACTACTAGAGTAAGAAGGGCCAAGAACACAAGAGGAATCTTAAGAAGTAGAATTAAAAATCTCTGTCAAACCTCTTCTTTACGTATCCATTCCTGGTTAGCCTTGCAATGTTTCCCACATTATGGAAATGGCATTGCGATGAAGGAGAAAAAACTCATGCAAATAGAGTAAATAGAGGTAAGACCATTTTAGGTCTGGTTAGCTTTACATGGTCGTGGGATGGGGCTACCTGGATAGACATTTTCAAGAGGTTTGAGCTGTGTAGAATTGTATCATGAAGAGAAAGGATATGAAGGTCATATAATGATCACCTTAAGCATGAGATTTGAAAGAGACTGTGGATGAGCATTTGAGAGCCTCTGGGTACCGCTCACTTGTTACTTCTTCTGTCAACTGTTAAGTCAGTAGTAGGTAATAAATAAGATAGTTGCAGTTCCATGTTATCCTATTGTGTCTACTCCTCATTTCTGCCTGGAAGGAAATCCCAGATTAGGATTGCCAGATAAAAGATAAGATGCCCCATAAAATTTGAGTTTTCAATAAACAATTTTTTTGTTATTTTTCAATAAACAATAATTTTTAATAAAAGTATGTCCCGTGCAATGTTAGGGATATACTTTTAAGGAAGTATTTATGGATTATCTGAAATGTAAATGTGACTAGGTGCGCTATATTTGTCTTTGCTAAATCTGGCAATGCTACTCCACATCAAGGACTAAAAACTAAGAAAGGATGGACTTTGGTCTTTCTGGTCTTTCTGGTGCCCTTGGTAGGGATTCAGAGAGCTTGGATGGCTCTCTCTGCTGTATTTACCATACAAAGGGCTGGATCCTTCCAGGAAGTGATGCTACACTAAAGTTTATGTGTCTTCTTATATTTGTCTTTGCCCATCTATCTCTAACCCTGCAAGGAGCTGCTATTCATTCATTCTTCCCTTTCCCAGGTATTCTTTTATTCTAACTTATTCTGACTTATTCTGTTTCTAATACAGTCTGCTCAAAGAAGGTCTCCTGGGGCAATGGCATGCTTGAGTTACTCATTTATAAATTTCCTGCTACTGTGCATTTTCATTATTGACAGTTGCGTTGCAGAAGAATTTTTCTTGCAAATGCAGGCCAATTTTTCCATGAATTTTAGGTGATCTCCTATTTATAAATAAAGCCACTGAAAGGGTTTTTGTCATTATTTAATAATGTAACCACAGAAAGAAAAACAAAACACCAGAATCAATATCCAGGACTGAGAATCCTGTGAGAAGAAAAACATTTGGCAACAGCAAGTCTGAGAAGTAGTGACATTTGTGTCCCAACAGATAGGCAGAGTTCAGTAATGGGCAACTTAGAGCAATAATTCTTCAGCTACCCACACAGCAAGAAGACAGTCAGCCTTTTGGAGATATTTCATTCTTCTAATAAATGTTAAGGTTTAGGGATAAGTAGTGGTAGGAATTGAAGCTCATACAGAATCCAAATATCACAGCTGGAATATGCTATTAGAATCTCAATAGCATTAGTTTAGGAGTAACTACATAAACAGTCTTTCAAAAATTATTTTATTTTTAATTTTTGTAGGTACATATGAGGTGTATATACTTATAAGGTACATGAGATATTTTGATACAGGCATACAATGCATAATAATCATATCAGGGTTAAATGGGGTATCCATCACCTCAAGCATTTATCCTTTGTGTTATGAACAATCCTATTATACTTTTCTAGTTACTTTAAAATCTATAAATAATGGTACTGTAGTCACTCTGTTGTGCTATCAAATACTAGATCTTATGGGTCACTCCATCTAAATATATTTTTATACTCATCAATCAACCCCACTTCCCACATCAACAAACGCCACTTCCCCCATCAACCAACCCCACTCCATCCCCTCCACCCAGGAGTGGGTGGAAGTGGTGAAAGAGGGGAAGTGGGGATGGTTGAAGGGTACAGATATTTTACTTTTTAGCTCCCACAAATGACAAGAACATGCAAAGTCTGTTTTTCTGACACAGAGTCTTGATGTTAGGCTATATCAGAATTTTTAAACCTTGGCATTGTTGACATTTTGGGCCAGATAATTCTGTTGTGGGGGACTGTGCTATGCATTGTGTGATGCTTAGCGCAGCCCTGGCCTCTATTTACTAAACGCCCGTAGCAAACTCCCAGTTCTCACAACCAAAATTGTCTCCAGACATTAACCAATGTCCCCTGGAGGACAAAAACTGCTCCTAGTTAGGGGTAGCCACTGGTCTGGAATAGAAGAGTGATGTTTAATCCTGATGGAATGTTAGAAGAACCTGGAGGGTCTTTTAAAAATAATGATTTTCTGACACCACCCCAAAGATTCTGACTTAATTGTTCTGGGAGAGAGCTCACTTTGTTGTTTTTCATATGCAAGTAGAGTTTAAAACTATTGAGCTAGATCTAAAAAAGAAAAAAAAAAATCCTGTTTGCAAAAGTATCATTCTTGGAAACTTAGAGATGAGTCCATAAATGTGAATTAAATAGCCTCAGCTATTGAAAAAGAAACAGACAACGGGGGGCAAGTCTTGGTAAAGCCTGGGCCTAGAAGAGCACATTCACTTTCCCCAGTTTAACTTGTTTTTAATGGCATTTTTAAAAAAACAACATTCATATTTTAGGCTGTAGTTGTATTAGTAAGGTTGCAAGACAGAAAAAGGGGCAGTAAAAATAAAAAAAGAATTAAGAAATTGCAAACTGGCTTTTTTTTTTTTTTTTTTTTTTAAAGGAAGTTTTTACTTACTGGTTTCTTTTTTTTTTATTTTTAAAAAAACCTTCCACTAAGTTTATCTCATGAGATTGCCAATTTGGGAACATCAGCAATCATGGGTGCACCTTTGTGGTGATCAAATATTTGAAGGCACGTTACCTTGCATGCCTTTCCTAAATCTTACACAGAGTGAGTAGTAAAGAGTTAAAGGACAGCCTGCTGCTCAGCTCTGGGATTCTAATGCAGGCAAGCTTTTACTTCTGCGGAGCTCTTCTAAGAGAGACAGCCTAGAGGTATTCCAGAGAACGGGTTTTTGTTGCTTACTGTTAACAGTCTTCCTAGGAACGTTTGGCAAGGCATAAAACATTCCTTTCACAGTACAGAGGGGACTTGTGGCCAGAGTGTGCAGATTTAGAGACAGGTGGCACCAAGAGGGTCTTTAAAATTAGTGACGCTTGCTGATGGCTGCACTGCAGTCACAATCTATCCCTACCATATTTCAAAAGTCCTCATATTTTATATGTATAATGTCACTAAGTCTGGTTTAAATAAATGCTAGATGATAAAGTATGACTTTATAATGAGTACTTGTTAACGTGCTCTCTGTACTTTGAGGAGCAGGGCTAGTGATTACTCCTCACTTGATCATTTACACTAAGACTCTCAGATGGACTTGATGCTTCTTGCCTATGGACAACGCTTTGCTTTCGCTTCTGCTGTAGAAACCTTGAACACCCACTTTAGTTCTACCACTGGGTAAGAACTTTATGATGAAAACTTAGATCTTGAGGATCTTCTTGAGACTCTCCTGCTTCTCAACTCAATGAAAATCAGTGCAACACAAAAAGTAAGTAAATCCTGTGAGGTGTGAGCCAGCTGACAAGGAGTGAGGTCAGTCCAGAGCACAGAGAGAAGTGTTGGGGACAGCTTGGGCAGCAGAGACGCAGCTGCGGCAGTAGCAGAACAGTAGCAAATGTCCAATGTGCAACTGCAGGAGAGAGAATGTAGGCACGAGAGAAAAGTCCGAGACAGAATGAGTGAATTATGAAAGGCCGGGGTGGGAGGGGAGAAAGTTTAATCATAAGAGTTTAGAGCCAACAGGACAAGTAATGATGTGTAATAAGCCCAGGTAATACAGTACCCAAGGAATACAGTAAAAACAAACATATCAGTAAAATGAGTCATACTTACTGATATGAGGACATGGGTTTTCTTAAATGCTTCCTTGCACCCTTTTTGGAGCTGGCATTTCTAGTACTAAGGTAATTCAGTGGACCCTTTGTAATATAACTCTACTCTGAAGAAAACAAACATTCTTTTAGCAGCATAAGCTAGTCATTAATCTTCACAACCTGGAAGATCTGATGGTGTGTTACAAAGTTCAGTACTGCCCTGAAACCCAGGCAAGAGGTGCCACTTTCCTGTTCTTTAGAGTGTCTGGCATATGAGAGACTCACACAAATCAATGTATTAGGAACACACAGGTGTCTCTGTTTTTTTGGGATGCAGTACTCAGCACTGGCACCACTTGACCCACGACCATAAATATCTGCTTCCATAACTCACACCAGTCAGGCCCCGGAGAAATGATCCCCACATCTCCTGCCCTGTGATTCTAAAACAAAAGGCAAGTGCATCTGAATTCTGTGAGGGTTTGTGTGTTGCACCACCACTGAAATCAGAGAAAGGCACTGCCTTAGATGGTGGGGAGTGTTTGAGTGACAGAGGCACTTAGGTAAGAGAAAAGACAAATTAATTAACTTGTCAGATCCTTCCTCTCAGATAACATGAATGCAATAGATTCTTGCATAATAAGTATAGTTTCCCAGGAGTACCATGCATCTATTTTTTTGCTTCTCTTCATGTTCCAATTCATAGTTCCAGAGGTACTTCTGAATTAAATCAGTTTTGTAAAATCAAACATGGTTGTTTAAGGACATTTTACAACATCAAGCATTCCATGTTAGTCTTAAATTTGTATATATGTATATCTATAGGCTTAGTTGTAATGATTTATGTATTGGCAATTAGATGAAAACAAAACACAGGAATTGCTAAAACTTTTATTACCATGAAAATATTTACTAAGATTGAATTACTTTTCCAAAAAATTAAGTTTAGCTTTAATTAAATAACTTTAAATTTTCATATTTATATGTTGTAATTTGTAAATTTGCTTTTAATGTTGGATAAGCTTGATAATATTATCAAATATTAGATAAATGTAATATTAGAAGAAAACTTTTTTGAAAAGATATATAAAAATAATTTCATTCAAAATTTTTATATTTAATTTAAATTTTTAATGAAAATATATCTAAGTTTTGTACGCTTTAAATGTAATTATGTTTGATAATTTAATCATTTACTATTCGTTCTCTATTGCTGCCCTAACAAATTACCATAGTTCAGTGGCTTACAAAACACAAATTTATTATCTTACCATTCTGTGAGTCAAAATTCCAAAATAGGTGTCACTAGGCTAAAATGAAGGACTGCATTTCTTCCTGCAGGCTCCAGGAGAGATCTATGTCTTACTCTTTTCGGCTTCTAAAGGCTGCCCACATTCCTCGACTAGTGGCGTCCCTCCTTCATCTCTAAACCCAGCAACAACAGGTTGAGTCCTCATGTCACATCTTTCTTACCTTTCTGTCATCTCATCTCGCTGACTGCTGCTGGGAAAAATTCTCCACTTTTAAGGGCTATCATGATTAGACTATGCCCACTAGATAATACAAGATCTCAGATCCTTAACTTCCATCACATCTGCAAAGTCGCTTTTGCCTCATAAAAGAGTCTGAGGTTTAGACGGGAGATCTTAAGGGGGCTATTAATATGCCTACCATAATCACTGAGAATAAGTACAAGTTAAGATTATAATAGCAATAGAATATACAAACGTGAAGCTCCAAAAGAACAACAACAACAAAAAAGGTGAACAGGAAAAAGAAACTGAAAATCTTTAAAAAGGCAGTCTGTTTAAATCTATAAAAACTGGAAAAAAATGAGAGTGGACAAATATCTGGTAAGCATGATGGACTTAAAATTTGTGACTAGGGCATTACATTTTTTATATTAATATAATGAAGATTGAATTACTGATCAAAACAATTAAAAAGCAAGAGAACTATTCTCATCAAATCTGCAACACGAAAAGTTCAGACAAAATTCCAACAACTTCACATTCTGAACTAAATGAGGACTAATTACCAGTTCGAGCAATGAGAATATATGAGGTCCTCCGTTTGCACTTTGCCAGGGATCTGAAAACGTTGGGAGTAGGTCGGCTTCACCCTGAAGCCAGACCATCGACAGCCACTTTTCCCTCCCTTCTCCACCCACAGGTCTTAGGCCCTCATCCTTCCCAGCCTCAGAACTAGTCTCCAAAGAAGAGGAAAGTTAGAGGAGAGAGTAAATCGTTGAATAGGATGAAGGAGATGTGGGAAAAAGAAAAAGAGAGGCTGCAAGAGAGAGGGTCCCAGGGATAACTCTGCTCTTGGAAGGGTGGCCACAGTCATGTGGTCCCAAGAGGCAACAACAAGCTTAGGAAGCCAGAGAAACCAGTTACAATCACTGCTACTCTTTTCGATTCTGTGTTGTTTAAGAAATATCACCCGCCAGGAGTTCTCCAGAAACATTTTCCCTGATTCCATGTAAGTGCTCAACCAGTGAATGGTAATCCCATTTTGGTTTAGTCTGTACCATCCCCTATTCCAAAATAAAGGGAAAAATGGTGGGTTTATATCTTAAATTTTCTACTTTACTAAACTCAAGGGAAATAGCCAAGCAAAAACGAAAGCTGAGACTCTTGCTAATTATCCTTTCCATAGAATGTTTGCTAAAATTCCTTGTCAAGGAAGGAATAACAAAGCTAGTCCACGCTCTGTATAGGGTGTTTCCAATTAGTTATACTTTAAAGTATAAGTATTTAACAAAATCTATAAATTTTGTTAATTATTTACTTGTAGTGAAAAATGAGCCATTCTCAAGCAAATCACTTTTTATTACACATTCCAGAGAATAACCATAAAAGGACATTTATTATAGCAAAAATAACCACATCTGGATGGAACTTCAATCACCAGTATTTACTAAATAAATGCTCAGAAAAAAAATAGTTCATCTTTAATTTCAGTCATCATTAATAAAAGCTGAAGTGCCTCTTCAGATCTTTTGATCATTTTCTGTTGGATTGTTTTCTTTTTACTGAGTTGCAAATGCTCTTTATATATTTTGGATACAAAGCTTTATCACATAGGCATTTTGCAAGTATTTTTTCCAAGTTTTTTTATCTTTTCATTTATTTAATAATATCTTTCAAAGAACAGGAATTTTATAATTTTTATGAAGTCCATTTATAATTTTTTCTTTTATGGGTTGGTGGGGGTTGGGGGTTGTGTTGTCCTAAGAAATCTTGGCTCAACACAAAAAGATTAGTTTCTATATTTTCTTCTAGAAGTTTTATAGTACGATCTCAGATCCATTTCAGATGATGAATAAGCACATAAAAAAAGGATACTCATCGTTAGTCATTAGAGAAATGCATATTAAAACCATAAGGAAATACTACTATATACATATATTAGATAGGATGAAGAGCAACTGGAATCTCATACAGTGCTGATTGAAATGCAAAATGGCAAAACAACTTTAGAAACCAATTTGGAAGCAGCTGTACTGACATGGAATTTTGAGCTGGAAGAATCTTAGAAAAAGAATACTTTACCACCTCCCCCATTCTCTTCACCCTGGGGAACTGTTAAATGAGGAAATTGTGGTTCAAGGAGGAACTTGTCTATATGCTTTCTCAGCTTTCCCGTGGTAATTACCATCTTGATAATATAACGTAATGTATGTATATGTTATCAAATAATATAATATCTTCATCATATATTTATCATCTTCATAATGTTAGCTGTCTAGTGGTAACTTTTTTTTGCTCTTTATTGCCTCCCTCTTTTTTCCCTCTTTGTTGTTTTTTGTCATACAATTATGATATATGTGTATATATTCTCACTGTAAAGATGTAAACAACACAAAGATTATTGAACAAATCACGAAAGTAACCCTTCCTTCATTCTTACCCTATCCAACCCTCATCTCCTCAGAAGAATACACCATTTTAGTTGTAAATGTTTTTCTAGCTCTTTTTCAATGTTTCTACCTATATGCATGTATGTATAATGTATATACATACATATATACATACATATTGATATATACATATATAGAGGTATGGTTTTTTAACTTAAATGGAATTGCATTGTGGATATTGTCCTATGACTTGCTTTCAACCAAATTATATGTCTTGGAAATACATACATATATTTAAAAAATATGTTATGTATATGTAACATACTATATGTGCATAATATATATTACATAGATATAATAAGGCCTAGGAAGAAATTGTGTGCAACCTCTAGTACATCTTCCTCTATATCTACTGTACATACATACAACCCATTCTTTTTTTAATTTTTTTATTTTTTTAGACAGAATCTTGCTCTGTCGCCCAGGCTGGAGTGCAGTGGCACAATCTCGGCTCACTGCAAGCTCCACCTCCTGGGTTCACGCCATTCTCCTGCCTCAGCCTCCCAAGTAGCTGGGAATACAGGCACCTGCCATCAGGCCCAGCTAATTTTTTTTTGTATTTTTAGTACAGATGGGGTTTCACCGTGTTAGCCAGGATGGTCTCCATCTCCTGACCTCGTGATCCGCCCACCTCGTCCTCCTAAAGTGCTGGGATTTACAGGCGTGAGCCACCGCGCCCAGCCACAACTCATTGCAGAGTAGTCCAAAATATGGATGGACTGTAGCTTAATTACTTATTCTCCCATTGATAGACACTTAGGACTTTTCTAATTTTTATAATTTAAAAATATGCTGCAATTAACAAACATTCTTGTGTATCTTTTTGCTGTATGTATGCATATTTCTTTAGTATGGGTTTTGGAAGAGGAATCACAAAGGAGGCATAGAATATAAATATTTTTATTTTGAAAAATACAGTTGTAATTTAATAACCCACCAAAAGACTCTAACAGTTTAGATTCACATCAACAGTGTAAGAGCATGTCTGTTTTACTGCATCCTTATCCCCACTGGTTATAATACTTTTAATTAACAATCTTATGGATGAAGAATACTATCGCAATGTTGTTTTAATGCATTTTTGCAATTACTAGTGAGATTGAACATTAATTCTTTTATTTTATGGATCACTGGCTTTTCTCCTTCTGTGAACTACCTGTTCACATCCTCTGCTTTTCAGCTCTTGAGCTGTTATCTTTTTCTTATTGATTTATATGAGCTCTTTATATATTCAAGATATTAATCATTTGTATTTTATGTATATGGCAATGATTTTCTTCCAAACCAATGCTTGTCTTTTATTTATTTATTTATTTATTTATTTGAGACCGAGTCTCGCTCTGTCGCCCAGGCTGGAGTGCAGTGGCGCGATCTCGGCTCACTGCAAGCTCCGCCTCCCGGGTTCACGCCATTCTCCTGCCTCAGCCTCCTGAGTAGGTGGGACTACAGGCGCCCGCTGCCACACCCGGCTAATTTTTTGTATTTTTAGTAGAGACAGGGTTTCACCGTGTTAGCCAGGATGCTCTCTATCTCCTGACCTCGTGATCCGCCCGCCTCGGCCTTCCAAAGTGGTCGGATTACAGGCATGAGCCACCACGCCTGGCCAATGCTTGTCTTTTTATCTCTGTTTATGGCATCTTTCATACTATGGACATTTTTATTTTTATTTTTTATGTTGATTTATTCTTGAATTGTATACATGTTAATTATACCTAAGTTATTGTAATACCGTTAAAGCCAAGTTCTACACATATATTTAATTTGCTTTCCCAATAGGTCTCTGAGGGAACACATTTTTTCAAATCACTTTGTTTCATCTTTTTTAGGTGTTGATCAATTATTAAGGAGTTTGAAATAATCATTTAAACGGAATTCTTCAGATGAAAACATAAAGACATTTATCGGGTCAGAGCATTGGTCGTTTCACATACTCAGGATCAGTGGCCTGGGTGGGCAGGCACTGGGTGAATGGAGAGCTGCAGGTATTGGAAGAGAGCCCAGTTGGATATGTAGTTTCCAAAGATCATCAAGGCAGACAACCAAAGGGAAACCGTGGGAAACACCTGCTTTGGGCCATCTAAGATGAGATGATAAAGTAAGGAAAGAGTTGAGCCCAACACAGTGATAGCCAATCTGAAAGCGGGCAGAACTGACAAGACCAAACAAGTAGGTGAACTGGCTGCAGGCAGCCAGCCACCACAGGGACAGCGTGTACTCCAGGGACAAGCTCAAGGCTATAGGTAGTTAGTTCAAGGCTACTAGGGTGAGAAGAGCAGGAACTGAGTTCTATACCAGTGCTTCTCAAAACTAATGTGCATCCTAATCACCTGGAAATCTTGTAAAAATGTAGATTCTGATTCAGTGAGTCTGAAGCAGAGCTTAAGATACTACATGCTTAACAAGAGCCTAGTTGATGCTGACACTGCTGGTCCCTGGAGCTCTCTTTGAGTAGCAGGCTTCTGGAAGGCTTGTGTCACTAAGCACAGAGAAGCCTCACTTATCAAATCTGCACCAAAACAGGAAAACTAATGTGAAGAATAATGTGATGCACACGTCAGAGCATGAGGCAGTTGCTTTGTCCCTGAGGTTGCGCTCCAGATGGCTTCCTAAGATGCGACAGGCTGATCTTGTGCGTGGGGGTCCCGGAGGCTTGGGCCACGGGAGAGACAGGACCTCAGAGGCTGGGAGACAGGCAGAGACAGAAGAGTGACATCCTGCTGCTTTTGAATTTGCACATTCTGTAGAATAATAACAGCAGTAAACTGTTACACAATATCTATTCTCAGCATCTTGAAGCCCTTTCACATATTGTTACTTCCATTAATGGGGCCCTTTGCTGCTATTTCTACTTTTCTCTTCAGCTATCAACAATATGGCTTTCCACACCTCCATCAGACAGTAGCCAGATGAAATAAAATGTGCCAGAATGAAAACTTGTTCATTTGTCTACTTTTTGCCAAGACTAGACAGGCAGGAAATTGAATGTATTTTTACAGAAAAGGTTTTCAAAACTTTTTCCCCTCTGTGGCTCATTTAGGTAAACTAAAAGGCATAAGACCCACCTAAAACATGGGTTCCCGCTTTTTATTGGAGAAAGAACATAGTACTTTAAAAAAATACATAAAATAATAAAAAGGAAAGACAAAGATAATGAAGGTTGTACATGGTACCAAATTTTTGTATCCCATAATAACACATGAGTAGATCACTACTAAGTAGGTTTTAGTGACATATAGGAAACATTAAAATCTACAGAAATTTGCATTATTTTCTTTCAAAAAGGATCATTTCACAGCCTTTCAGGGGGAACCCATTGCCCACAGGAACTCATGCATTCCATGCTTTGAGGATCACTAGATCTAAGAAGCCTTCCTTGGAGGTTCTAGCCTCCAACCCTTATTTTAGTAAAAGAAGCTCCAGTTTTATCTGTTTCTAAGTCAGACTACCACACAACATTGGGCTTAAAGAAAGGTTTCCAGGGCTAAAGCAGACTTTGAGGATTACTAATTCCGAGTTAAATTTCTGTGTATTATCTCTGGATTTGACTTATTCACACTGGACTATCACTCATAAATATACATAATACAGAGTTAACTATTTAAATTTATAAAGAGAGTATTTTCCTTTTTTATGAGCAAAACATGCTGCCAACTACTTGGACCACATACTGATCCATAAATACTGACAGCTTTGTAATTGGAAATAATAAATACACACTAATGAAGCATCTCAAAAGGGAAGAGCCACAGGTAATCTGAGTGATTAGGCATTCATGTTAGGTTAGGCTTTGATCATTGTTTTTAATCACAATTTCATTGCAGTGCATCTATAAATCCATGTCCAGAAGTATGAAGTGGTTCTATAGTAAGAATAAGATGCTACAGATAATGCGACTAAATAAGACACTATAGGTAATGACACAGATTCAAGTCTTATTGTTGATGGGAAGAGGTCAATAATGGATGATATAATATACTACAGCAATGAGAATTATTGAATGTTTTCCAGACTCACTTGTATAATTGGCCATAACAGCAAACAAAAAACAGGTTCTGATAGCAAAATGATATACAGTACTAACAAAGGTGAATCTTGAGGTGAACCTTCTCTTTATAAGTTTAAATAGTTTACCCCCGACCTTTTCCCATAGTAGAACAGCCTAAAAAGTATCTTTCAGTAGAATGCTAGTGCTTATGAGGTTTTCTTAAGATATCATTTTTCAATTAAAATTTATTTCACAAAAGACTCACATCCTTGCCAGCCTTCAGGGTGAGTGTTGATTCAGGCTGTGTCCAACGGCAACGATGAGTGAACTTCTCACCCTCAGAATCACATGAGCATTCCTGAGATGTTTTATCAGAGTGATACCAACTTCATTATTAGAATATTGAGTCCCTATTTCCTATATTCAATGTCCTTTCAAGCCCTAACTTTGTCCGGGTTGAAGGCAAAGATCCAAATAATCACATTTGTCTTTGATAACTGAAACTGGGAGAACTGGGACTGTCTCAAGAGTTCTACGTGACTGTAGGTTGCAAGTACTGTGGTTGCATCTCCAAATATTAACCAATCCCAGTGACAATTCAATGGGGTCTCCTGAACCATGATCCTCATGTCTCCAGTGAAGGAAATGGGCAAAGGGGATTCAAAAATCCCTTTTGGAGGAATAGGAAACTTCTGCTTTCCTTCATTTCATAACATTTGCGATGGAACAAAGGCTTTTTTAGAATGGAGCAACCAGATCCTTTTTTGGGGGAATCAGCTTAAATGTCCATTCTTCTCATACTACTTTTATCTATGTGATCCTATTCTTTTCTGTTGTGGATTGAATCATGTCCCTCAAAAAGATTGAATTTAGAGTGTGCTCTAAATTCAATGTGGAGAAATTTGGACACAGAGGCAGACACACAGGGAGAACCCCGTGTGACAATGGAGGAAGAGGATGCATTTATGCTGCCACAAGCCAAGGAACACCAAAGATTGTCAGCAGCCACCAGAAGCTAGGATAAAGGCATGGCACATCACTCCCTCTGAGCCCCCAAAAGGAGCCAAGACTGCTAATACTCTGATCTCGGACTTCTGGCCTGAAACAGTGAGAGAATAAGGTTCTGTTGTTTCAAGCTACCCAGCTTGCGGTATTTTGTCACAGAAGCACAAGGAATCAAGTACATTTTCTTTCTCAGCACTTGTGATAATTTTATTTTTTCTTTACTCAGTGGTTGTTTCACACCTATGTCCCCATCAGACTGTAAGCTTAAAGAGACCTGGATCTGGTCTGTCTTCACCACTGTTGATTCATTACCAGCACAGTGCCTGGCCCATGGTCACTGAATAAACGTTTGTTGAGAGAATGAATGTGCTTAACCAGAAGTACTATTGACCTATTAGGCCAAGTTCAAGGTGCCTAACAGCTCAGCTGTGAAGGATACCTCTCCTTTCAGTCCTCTGTTACATATGTCCCTGATAGATGTGTTATTTGTATCTCCTCCTGGCCCTCAAGTTTGTTTGAGGGCAGGACCCTTTTTTGTATATCTGTAGAGCTTCGTAGTACCTAAATACTACTTTGCATATATAATAAAGTTTCGATAAATATTCATTAAATAAAGAAATAAATGAAATGACTAAGTTTTCTAAGATGTTACAACTAGATTGAAGATATTTAGCTCATTATTTAACAAGAAAACTATGGTTAATTATGGTGTCCTGTGTGAAAATGGTTATAGTTTGTTTTTTAATTAATATAAGCATGTATGTGCATTATCAGTATACACAATTTGTGGTATGAGTGTTTTGTGTCCCTGCACACAGACCACGGAAATCCTGAGAAACAAACTGCCACCCCAGAGCAGGTGCCTAACACAGAGACTTTTAATCCTTAAAGTTTTTCTATAACTAAGCAATGTTTTTTCAAATGCAATAACACTGATATGCAGACATATTGATTGTCCACTCACAAAGCCATTCCTCAATATCATTACAACATGCCTCTTTGAATGTCATTAAAAATAGATGTCTCATTTTTCTAGGACAAGTTGGCTGAAGTTCTGCTTGAAAACTGGTAATAGAAAATACAATTTCTCAACCCGCTTTGGCCTTTTAATTCTGTTCTACAACCTTGCCAGTTCACTTTCAAAGTCAAGGGATGCATCTTGCAAAACCATGACATCTTTTGAGTAACTCCTTCTGTTCTTAACACATATTCCCAGGAGCTTAATAAATATTGTTTTTGCAACTTGTTTAGTGGCAAAATAATGAGTCCTTGGTGTATGCTTATCCTCTGCTTTGCTATTAGAGAAGATATATTCAGACTGTTTTAAACAAATTAATTCAAGGGCAGGGAACAGTCCTAAAACCTGTTAAAATTCAAATACTTGGTCACTGTATGTGCAGCATGTGTGTTCTAGAAAGTCCTATTATTTTAAAATATAAATTGAATCTTGTTGAGAAATTAATGTCATATGAATATATTAATAACTGAAATGCTGCCAAGTTTACAAAAAGCCCTCAATGAAACTGTGACCTTGTATAGACAAGGGCCTGTGGAGGGACATTTTTAAACCATCTCTTTTTTTATTTCCTCATGAGATCTACAATGTAAGTGCATTAAAGTTGATGAATGAATTGCAGTGCAACTTTTCCTGCCTCTTTTGCCTTTCATTTGTCTATATTTCAAGCTTCACTGAAGTGATAGATTTTGGGCTTTGCCACATTGTCCTCTGATTGCTTCCCTCTGCTCCTCCTTTTCCTAGTGAATCTTTGTTTTACTGGTAGAAAAATCTACATCTTTGTATCTTGGCATTTTACTTTCACATTATCTCATAGATTTTATTTCAAGTTGCTATAAAGTTATCAACTTTTATTTTTAACTAATATTATTTTTAACAATTAGAAAATTGTTGACCAGGTAATTCCAGCACTTTGGGAAGCTGAAGCGGGAGGATCACGTGAGCCCAGGAGCTCGAGACCAGCCTGGGCAATGCAAGGAGACTGTCTCTACAAAATATAAAAATACATTAGCCAGGTTTGGCGGTGCATGCCTGGGGTCCAGCTATTCAGGAAGCTGAGGTGGGAGGATCACTTGAGCTGGAGAGGTTGAGGCTGCAGTGAGCAGTGATCGCACCACTGCACTCCAGTCTGGGTGACAGAGGGAGACCCTATCTCGAAAAAAAGGAAAAGAAGAGGATTTTGCTGGCAAGATGGCTGAATAGGAATAGCTCCGTTCTGCAGCTCCCAGTGAGATCAATGCAGAAGGCAGGTGATTTCTGCATTTCCAACAGAGGTACCTGGTTCATCTCACTGGGACTGGTTGGACGGTGGGTGCAGCCCATGGAGGGTGAGCAGAAGTAGGGTGGGGCGTTGCCTCACTCAGGAAGTGCAAGGGGTCCCTCTTCTAGCCAAGTGAAGCCGTCAGGGACTGTGCCATAAGAACAGTGCACTCTGGTCCAGGCTTTTCCCACAGTCTTTGCAACCCACAGACCAGGAGATAACAAGCGGTGCCTATGCCACCAGGGCCCGGGGTTTCAAGCACAAAACTGGGTGGCCATTTGGGCAGACATCAAGCTAGCTGCAGGAGATTTTATTTTCATACCCCAGTGGTGCCTGGAACGCCAGTGAGACAGAACCGTTCACTCCCCTGGATAAGGGGCAGAATCCAGGGAGCCAAGTGGTCTGGCTTGGCGGGTCCCACACCCACGGCGCCCAGCAAGCTAAGATCCACTGGCTTGAAACTCTCGCTTCCAGCACAGCAGTCTGAGGTCCACCTGAGACGCCCGGGCTTGGTGTGGGGAGGGGCATCCACCATTGCTGAGGCTTGAGTAGGCGGTTTTACCCTCACGGTGTAAACAAAGCTGCCTGGAAGGTCCAGCTGGGCACAGCCCACCACAGCTCACCAAGGCCGCTGTGGCCAGAGTGCCCCTCTGGATTCCTCCTCTCTGGGCAAGGCATCTCTGAAAAAAAGGCAGCAGCGCCAGTCAGAGACTTATAGATAAAACCCCCATCACCCTGGGACAGAGCACCTCAGGGAAGGAGTGGCTGTGGGTGCAGTTTCAGCAGATTTAAACGTTCCTGCCTGACAGCTCTGAGAGAGCAACAGATCTCCCAGCACAGCGTTCAAGCTCTGTTAAAGATCAGACTGCCTCCTCAAGTGGGTCCCTGACTCCCATGTCTCCTGATTGAGAGACACCTCCCAGTAGGGGCTGACAAACACCTCATAAAGGAGAGCTCCAGCTGGCATCTGGCAGGTGCCCCTCTGGGACGAAGCTTCCAGAGGAAGGAACAGGCAGCAATCTTTGCTGTTCTGCAGTCTCAGCTGATGATACCCAGTCAAACAGGTCCTGGAGTGGACCTCCAGCAAACTCCAGCAGACCTGCAGCAGAGGGGCCTGACCGTTAGAAGGAAAATTAACAAATAGAAAGGAATAGTATCAACATCAACAAAAAGGACGTCCACTCAGAGACCCCATCCAAAAGTCACCAACATCAAAGACCAAAGGTAGATAAATCCACAAAGATGGGGAGAAACCAGTGCAAAAAAGTCTGAAAATTCCAAAAACCAGAACGCCTCTTCTCCTCCAAAGAATCACCACTCCTCACTAGCAAGGTAACAAAACTGGACAGAGAATGAGTTTGACAAATTCACAGAATTAGTGTTCAGAAGGTGGGCAATAACAAACTCCTCCAAGCTAACGGAGCATGCAAGGAAGCTAAGAACCTTGAAAAAAGTTAGAGCAATTGCTAACTAGAATAACCAGTTTAGAGAAGAACATAAATGACCTGATGGAGCTGAAAAACACAGCACGAGAACTTTGTGAAGCATACACAAGTATCAATAGCCAAATCGATCACGTGGAAGAAAGGATATCAGAGATTAAAGATCAACTTAATGAAATAAATTGAGAAGACAAGATTAGAGAAAAAAGAATGAAAAGGAATGAACAAAGCCTCCAAGCAATATAGGACTATGTGAAAAGACCAAATCTATGTTTGACTGGTGTACCAGAAAGTGACGGGGAGCATGGAACCAAGCTGGAAAACACTCTTCAGGATATTATCCAGGAGAACGTCCCCAACCTAGCAAAACAGGCCAACATTTAAATTCAAGAAATACAGACAACACCACAAAGATACTCCTCGAGAAGACCAACCCCAAGACACATAATCGTCAGATTCACCAAGGTTGAAATGAAGAAAAAAATGTTAAGGGCAGCCAGAGAGAAAGGTCAGGTTACCCACAAAGGAAGCCCATCAGACTAACAGCAGATCTCTCTGCAGAAACCCTACAAGCCAGAAGAGAGTGGGGGCCAATATTCAACATTTTTAAAGAAAAGAATTTTCAACCCAGAATTTCATGTCCAGCCAAACTAAGCTTCATAAGTGAAGGAGAAATAAAATCCTTTACAGACAACCAAATGCTGAGAGATTTTGTCAACAGCAAGCCTGCCTTACAAGAGCTCCTGAAGGAAGCACTAAACGTGGAAAGGAACAATCGGTACCAGCCACTGCAAAAGCACACCAAATTTTAAAGTCCATTGACACTATGAAAAAACTGCATCAACTAACAGGCAAAATAACCAGCTAGCATCATAATGACAGGATCAAATTAACCTTAATTAAGTTAGCCTTAAATGTAAACGGGCTAAATGCCCCAATTAAAAGACACAGACTGGCCACCTGTATAAAGAGTAAAGACCCATCAGTGTGCTATATTCAGGAGACCCATCTCACATGAAAAGACACACATAGGCTCAAAATAAAGGGATGGAGGAATATTTACTAAGCAAATGGGAAGCAAAGAAAACAAAAAGCAGGGGTTGCAATCCTAGTCTCTGATAAAACAGACTTTAAACCAACAAAGATCAAAATAGACAAACAAGGGCATTACATAATGGTAAAGGGATCAATGCAACAAGAACAGCTAACTATCCTAAATATATATGCACCCAATACAGGAGCACCCAGATTCATAAAGCAAGTTCTTAGAGACCTACAAAGAGACTTAGACTCCCACACAATAATAATGGGAGACTTTAACACTCCACTGTCAATATTAGACAGATCAATGAGATAGGAAATTAACAAGGATACTCAGGACTTGAACTCAGTTCTGGATCAAGTGGTCCTAATAGATACCTACAGAACTCTCCACCACAAATCAACAGAATTTACATTCTTCTCAGCACCACATCGCACTTATTCTAAAATTCACCACATAGTTGGAAGTAAAACACTCCTCAGCAAATGCAAAAGAACGGAAATCATAACAGTCTCTTAGACCACAGTGCAGTCAAATTAGAACTCAGGATTAAGAAACTCACTCAAAACCGCACAACTACATGGAAACTGAACCTGTTCCTGAATGACTACTGGGTAAATAATGAAATGAAGGGCAAAATAAAGAAGTTCTTTGAAACCAATGACAACAAACACACAATGTACCAGAATCTCTGGGACACATTTAAAGCAGTGTTAAGAGGGAAATTTATAGCACTAGATGCCCAAAAAAGAAAGCAGAAAAGATCTAAAATCGACACCCTAGCATCACAATTAAAAGAACTAGAGAAGCAAGAGCAAACAAATTCAAAAGCTAGCAGAAGACAATAAATAAGATCAGAGCAGAACTGAAGAGGAGAGAGACATGAAAAACCCTTCAAAAAAATCAATGAATCCAGGAGCTGGTTTTTTGAAGAGATTGACAAAACAGATAGACCACTAGCCAGACAATAAAGAAGGAGAGAAGAATCAAATAGATGCAATAAAAAAAGATAAAGGGGGTATCACCACTGATCCCACAGAAATACAAACTACCATCAGAGAGAATACTATAAACAACTACACAAATAAACTAGAAAATCTAGAAGAAATGGATAAATTCCTGGACACATACACCCTCCCAAGTCTAAACCAGGAAGAAGTTGAATCCCTGAATAGACCAATAACAAGTTCTGAAATTCAGGTAGTAATTAATAGCCTACCAACCAAAAAAAGTCCAGGACCAGACAGATTCACAGCCGAATTCTATCAGAGGTACAAACAGGAGCTGGTACCATTCCTTCTGAAACTATTCCAATAGAAAAAGAGGGAATCCTCCCTAACTGATTGTATGAAGCCAGCATCATCCTGATACCAAAACCTGGCAGAGACACAACAAAAAAAAGAAATTTTCAGGCCAATATCCCTGATGAACATTGATGCGAAAATCCTCAATAAAATACTGGCAAGCGGAATCCAGCAGCGCATCAAAAAGCTTATCCGCCAGGATCAAGTCGGCTTCATCTCTGGGATGCAAGGCTGGTTCAACATACGCAAATCAATAAACCATCATTCTCAGCAAATTATCACAAGAACAGAAAACCAAACACCGCATGTTCTCACTCATAAGAGGGAGTTGAACAATGAGAACACGTGGACCCAAGGAGGGGAACATCACATACTGCGGCCTGTCGAGGGATTTGGGGTTGAGGGAGTGATAGCATTAGGAGAAATACCTAATGTAGGTAACAGGTTGATGGGTGCAGCAAACCACAATGCGATGTGTATACCTACCTAACAAACCTGCACGTTCTGCACATGCACTCCAGAACTTAAAGTATAATAATAAAAGGCGCTGCCTCAGGATGTAAAGTGTAACAAGGGGGCTGGGGTGGGCAGCGTGGGCCTCTGAGACCTTTGGTTGCCCGTGTCCGCAGCTCGCCCCGCAGCCGGCTCCACAATGGTCCGCTCCGTTTGCCACGTGCGGATTCGGGTTCCAGACTGAAGGCTGCGTGTTCTCTGCCGCCCACAGCCCAAGTTTATTGTGGCAACCGCCGGAGCAGCCTTCCCCGCTGTGGAGGAGCCTGGGGCTACCCCTCAGCGGTATTTGGGGCTGGTCCTGGGGGAGCTAAGCAGGGTTGTGGCAGCACTGCCTGAAAGCGTGAGACCAGACTCTAATCCTTATGGTTTTCCATGGGAGTTGGTGATATGTGCAGCTGTACATGGATTTTTTGCTGTTCTCTTTTTTTGTGTGGAGAAGTTTTAGATCGGTTGGGAGTCGGCTTTATGTGGGAAGAGAAAAAAAGCTTGCTGTAATGCTTTCTGGACTAATTGAAGAAAAGCATAAACTACTTGAAAAATTTAGCCATGTTCAAAAAGAGTATGAAGGCTATGAAGTAGAGTCATCTTTAAAGAATGCCAGCTTTGAGAAGGAGGCAACCTGTGAAAAGCTAAACAGGTCCAATTCTGAACTTGAGGATGAAATACTCTGTCTAGAAAAAGAGTTAAAATAAGAGAAATCTAAACATTCTGAACAAGGTGAATTGATGGTGGATATTTGCAAAAGGATACAGTCTCTAGAAGATGAGTCAAAATCCCTCAAATGACAAGTAGCTGAAGCCAAAATGAACTTGACGATATTTCAAATGAATGAAGAACGACTGAAGATAGCAATAAAAGATGCTTTGAATGAAAATTCTCAACTCCAGGAAAACGAGAGACAGCTTTTGCAAGAAGCTGAGGTATGGAAAGAACAAGTGAGTGAACTTAATAAACAGAAAATAACATTTGAAGACTCCAAAGTACATGCAGAACAAGTTCTAAATGATAAAGAAAATCACATCAAGACTCTGAACGCTTGCTAAAAATGAAAGATCAGGCTGCTATGCTTGGAGAAGACATAACGGATGATGGTAACTTGGAATTAGAAATGAACAGTGAATCGGAAAATGGTGCTTACTTAGATAATCCTCCGAAAGGAGCTCTGAAGAAACTGATTTATGCTGCTAAGTTAAATGCTTCTTTAAAAACCTTACAAGGAGAAAGAAACCAAATTTATAGTCAGTTATCTGAAGTTGATAAAGGAAGAGCTTACAGAGCATATTAAAAATCTTCAGACTGAACAAGCATCTTTGCAGTCAGAAAACACACATTTTGAAAGTGAGAATCAGAAGCTTCAACAAAAACTTAAAGTAATGATTGAATTTTATCAAGAAAATGAAATGAAACTCCAGAGGAAATTAACAGTAGATGAAATTACCGGTTAGAAAAGGAAGAAAAACTTTCTAAAGTACACGAAAAGATCAGCCATGCCACTGAAGAGTTGGAGACCTATAGAAAGTGAGCCAAAGATCTTGAAGAAGAGTTGGCGAGAACTATTCATTCTTATCAAGGATGGATTATTTCCCACGAGAAAAAAGCACATAATAATTGGTTGGCAGCTTGGACTGCTGAAAGAAACCTCAATGGTTTAAGGAAAGAAAGTGCTCACAACAGACAAAAATTAACTGAAGCAGAGTTTAAATTTGAACTTTTAGAAGAAGATCCTTATGCACTTCATGTTCCAAATACAGCATTTGGCAGAGAGCATTCCCCATATGGTCCCTCACCATTGGGTCGGCCTTCATCCTAAACAAGAGCTTTTCTCTGAGGGCCCACTGAGACTCTCATCTTTGCTAACAGGAGGAGGAGGAAGAGGCTCAAGAGGTCCAGGGAATCCTCTGGACCATCAGATTACCAATGAAAGAGGAGAATCAAGATGTGACAGGTTAACCAATCCTCACAGGGCTTCTCTGACACTGGGTCCCTGTCACCTCCATGGGAACAGGACCGTAGGATGATGTTTCTTCCACCAGGACAATCATATCCTGATTCAGCTCTTCCTCCACAAAGGCAAGACAGATTTTATTCTAATTCTGGCACACTGTCTGGACCAGCAGAACTCAGAAGGTTTAATATGACTTCTTTGGATAAAGTGGATGGGTCAATGCTTTCAGAAATGGAATCCAGCAGAAATGATACCAAAGATGACCTTGGTAATTTAAATGTGCCTGATTCATCTCTCCCTGCTGAAAATGAAGCAACTGGCCCTTACTTTTCTCCTCCACCTCTTGCTCCAATCAGAGGTCCATTGTTTCCGGGGGATACAAGGAGCCTGTTCATGAGAAGAGGACCTCCTTTCCCCCCACCTCCTCCAGGAACCATGTTTGGAGCTTCTCAAGATTATTTTCCACCAAGGGATTTCCCAGATCCACCACATGCTCCATTTGCAATGAGAAATGTCTATCCAGCGAGGCGTTTCCTCCTTACCTTCCCCCAAAACCTGGATTTTTCCCCATAAACCCCACATTCTGAAGGTAGAAGTGAGTTCCCTGCAGGGCTGATTCTGCCTTCAAATGAGCCTACTACTGAACATCCAGAACCACAGCAAGAAACCTGACAATATTTTTGCTCTCTTCAAAAGTAATTTTGACTGATCTCATTTTCAGTTTAAGTAACTGCTGTTACTTAAGTGATTACACTTTTGCTCCCACTGAAGCTTAATGGAATTATAATTCTCAGGATAGTGTTTTCTAAATAAAGATGATTTAAATATGAAACTTATGAGTAAATTATTTCCATTTTATGTTATTCTGGATAGTATAACTATTTTAATTTGATAAACTAATCCACGATTATATAAACAATAATGGGAGTTTTATATATGTAATCTTGCAGGTAGGGAGGCTTTAAATTATAAAGGTTGTGTCTTTATGCCAAGAACTGTATTAACTGTGGTTGTAGACAAATGTGAAAGTAATTTTATGCTTCATTAAATAAATTTTAGTTGATTTTTTTTTAAAAAAAGAAAATGGTTAATCTATCATTTAGGTGCATCATCAGTTGTTTAACCATTCTCTCTTACTGAACTTTGGGTTGTTTAAAAAGTGTTGTTATTTTTGAATCATGGTTCAGTGAACAATTTTGGACACATAACTTTTTATCTGATGAGTTATTTCCTAAGGATCCAGCTCAGAAACTCAGCACATAAACCTAATAAGAAAAAAACAATTTGAAGTGGCTAACCTCTTATCCCAATAAAAATGTTGTATTTATGTTTGGATTTAGATGCCTTTCAGTGGTCATACCTTCACCTAACTTTTATGGATTCTACTTTTAACATGTAGAGTGACTGTTTAAATCACCTAAACTCACTGAGTTTTAAGTTCCTTTTTATTCAACAAGACTGGATTGTATGTTCCAGCTCCTCAAACTTAGTTACCAACCACCATCCTAGAGAAGTGAATTCACATGAGGCCTGTCCAGAAGAACAATCTCCCTTTCAGTGTCCTCATGCATGCAGTGACCAGAGACCAACCTTGATAAATTATGGAAAAAGTACAGCACATTCTGGAAGAGCCATGAAAGATCCAGATCATCTGGTGCTGGATAAGAATATTAATGGACAGGCTGGGCGCGGTGGCTCACGCCTGTAATCCTAGCACTTTGGGAGGCCGAGGCGGGCGGAACATGAGGTCAGGAGATCGAGACCATCCTGGCTAACACGGTGAAACCCCGTCTCTACTGAAAATACAAAAAATTAGCCGGGCATGGTGGCGGGCGCCTGTAGTCCCAGCTACACGAGAGGCTGAGGCAGGAGAATGGCGTGAACCCGGGAGGCAGAGCTTGTAGTGAGCCCAGATGGCGCCATTGCACTTCAGCCTGGGCGACAGAGTGAGACTCCGTTTCAAAAAAAAAAAAAAAGAATATTAATGGACAAAAAGATTAATGAAAGAACATATTGAAGCATCCAATTACCTGGTGTCTGCTCAAATGAGGAATCGGTGAGATAGGTCAGTTAGCAGTCAAGATTTATAAAAGAGACGATGGCCTTGGGAGGGGCTGCCCTACTCGACTTTTTAATGGCTAGAAGCTATTAAGGGCTAAGCCAGAACCCTTCAGTATGGTTCAGTGAGGATCCCAATTTGGGGTCCAAAAGTAAATGACAACTCCCAGGAACCATTAAGAATAAAAATCATGGAGCATTACTGAGAATTTGTGTTATCTAAGTCTGAGGAAAATTAATGTTAAGGAAGCTTTCAAAAGTCTAATATTTACACCGAATTCCAGGGCACCATGCTCTAAGACAAAGCACTCTGGTCCTGCCCCTCTCCTTTCCTCATGTTTTTTGGTTCTTGGGATCCTTAAGGGTCAATGTTATTCTTAAAATACAGAGCATCCTGGAAACTAAAAAAGTGGAAGATATTCAAATTCTAATGAATGTACTGGCAGTATTGTAGATCATGGAGTATAACATAAAGACAAGAATCCCTAGCCTCTTCCACCATACTTTGTAATGGTAAGGAGAAAGGATAGAATTTTGAGAAGTCTGGGAAGACAATGTATGATAACATCTGGAGAAGCTCTGCATAAGTTACTTTTGTTCAGGCTTAAGAAAAATTCTAGCTTGCCCCTGCACTGTCATCAGGTATCATGAAAGTAAATAAAACCTTTAAAGATTCTTCAAGCCAGCAGACTTCTATCTTCTCTATACTATCCTGTGATCCTAAACTCTTAACAGTTACTACGTATAATTTCCCTACATTTGCTACTAGTATTTTATCATACACAATATTACACTCAATATTTCAAAAGTGGATGATTCATCTCCCGAAGAGACTGCAAAATTCATGAGTTAAGATTTGAGAATACTATTTTAGACAAGATTTAGTCAGATTTTAGAGAGTTAGAAACCTGTAACAATTCTCTAACAATACTGCTTCTCCTTTTGTGTATTAAGGAATTTTTGTCTATCAAAGATAGTACGAGGTAGACCAGAAGATAACTTGCCTTCAAAATATCTGGAATGTAAAATGGCAACAGTAGTATTTGGGGACTTCGTAGGGGATGGCCAATATACACCCATTCTTAGAGGTACTGATGATATAATGTATAAGACAAAATCAAGTGGTCTCCATCACCATATAATGTTTAAAATGGCAAAGAGGGAGCAGAACAAACACCCTTTGCAAATCTCTTCATAGAATCTACCGTAATAAACTTGTACTTGCTTAAAGTGTGTCTCTTCAGTGGTCTTATTACCACTACTTTGGGGAAAATGAGGCTGCTTAAAAGATTAACAGACATTACATTTTACATATCTGTGGCAGAGAAAACACTATGTATTCACCAAACCACTTCTTTTCCTTCCCAGTCACTCGGGAAGAGGTCATTTCTTTGTCCCCTTTCATCTAATTGAGGTGCCGTGACTACTTCTAGACAGGCAATGTGAGCAGAAGGTATGCACACCACGTATAGGCCTGGTCTTCAAAAATCCCTCAGATATGATCTTCTCTCGTCTCTTTCATGGACAAACTACAGGCCATGTAATAAGGATGGTGGGGTTCCAAACTGAAAGAGCCTGGATTTCTGATTTACTGTTTTGAGAAGAGTTCACCAGGGAAACAGCCTGGAAATACGCACAGGAAAATATGCACAGGACCCTGTGTGAGCAAGATATAAAGATCTATTACATGGTGCCATTAAGGTGAGAGTATTGTGCTTATGGTATCCAGCATTAATTATCCTCACTACTACAACTTCTTTGTATCCATCATGTGGAAAAGTAGAGTATTTAATAAATGATTATTGAGTTTATTACCTTTTTTATATTCCAATCATTGCTAATTGTACGTTACCTCATTTCAAGGTAAAGGTGACCAAGGGCTAAAGCAGTGCTATCCAAACCAAGCCAGACATCAAAATCACACAAAACCTTTTGAAAATACAACTTTGAAGATGCCATTCACATAGATATTTATTCAGTGGGTTTTCAAATGGAACCCTGGAATCTACAGTCTTTAACAAGGCTTCCCAAGTTATTCTGATATACAGCAGGCAAATCTGAGAACCACTGGACAAGAAGAAAATAAAGGCTATATCTTTCGACAACAAAGACAATGCCTTAAACATAGAATGTATTCAATTAAAGCTTGTAGAAAGATAGGTTTGTGAACAGGCACAGGGACTAGCCTCGAGCAAATTAATAAGGGCAGCAATGTTTTTCACTGAAACCATTATTCCCCCTATTTTATTTCTTCCGGGGCTCTGTGTTTCCTTTCTCCTATCAAAATCCATTCTAAGGTTGGAGGTTGGGGGTATCTCTTGCCTACTCCATACAGCAAGGAATAAAATTAGTATTTCTCGAACTATCTGTGACAGCAGACCCATTGTAGGCCAGTACTTTTGTAAAATGCAATAAAAATTAACTTCTAGAAAATGAAATTTTCAAATCACAGACATTCAAAATACAAATTCCAATTTTTTTATTATTAACTGTAAGAAATTTAAAATTAAATCTCAATAAATAAAATTAAAGCAAACATAAGATAGAAAAAAATAAGCATTATGGATTGGCCCAGTCTGCAAACTGTATACACTTTGCCAAACATGGGCATAAATTACTGAGAAGCAAAATCTTCCATCTGTAAACATTTCCATTTCCATTGACAATATGTGTGAGGGAAAGGAGGGATGCTTCTGTTTTAGAATGCCAGGCGTCAGCTAACAAGTGACAAATACGTATTGAGACTGAGATCTCCCCAGCCTCTCAGTAGTCAGCAAGAACATGTTGAGGCCTCTGTTTTTGACTAAAAAATTGGCCAGTGCATGGGCAACATGCATAGGTCCTGAATGAAAAAAATAGCAGCAGCAGAAATTTAAAAGAATTTTCACAGCTAGGCCACAGTAAATTCTCAAGCCCTTCATCAGAAGCCACTGTGGGGCCTCATTTATGCCTTTGTTTTTATTAAATTGGATGTGATCTTAAGATTCTTCTGTCAAAATTCCACTAGCATGTGAAGGCACCAAAAGTTTAAAATGTAAAATTAACCCAAGTTAAGCTATTCCATTATTAAGCAATAGCAGATATATTTGTTATTATATGAGAAGAAAGTTAACAGGGAGCTAAGATTGATGTTACTGATAAGAAACAGAAACAAGACTTTAAAATTAAATAAATGAATTATTTATTTAATAAGAACCAATTGACAGATTCTCGATAAAGACTGTAAGATGTCTTAAAACATTAGGTGTATGGAGATAACATTTGTAACTTTGACAATTTATATGATGAGAAAAATCAAGGAATGTTATTGTTTATTGGCAGAGTTCTAGAATTACAATTCCATCATTCTGTTTTGGGGAAGTTTCCCTTGAAGTAAATGATAACAGGGCTTGAAATAGTACACCTCAGCATTTTGTTTATAAAACTGTGGAATAGGTAAGGTTTGTATTGTAACTGAACCCAGGTTCAGCTGCTTGCTGCTCTAAAGCTAGACATAAGAGAGGAAGGTTGGTGGGAGGAAAAGCGATTTTAATCGGAGAAGCAGCAAACCAAGAAGATGGTGAACAATAGTCACAGAACCATCTTAAATTTTAAAATTTACCATAGAGTGTTCAAAGGAAAACTTGGTATGGGAGGCATGCAGGAGGGGTGCAGGGGGCGGGGTCTGTGTGTCTTGTTCCAATGGCTATCTCAGATAGTCACCCATCTGGAGGTCTAGTTGGTATTATTTTGAATTCAGCCCAGTGGTGGTGGACTGTCAGTGACTCCTCGCTAAGCAGGAGGATTCTGCACTCAGGGCTCCATGCATGGTTTGTTTCAAGATTGGCCTCTGGAATTTCTCAAGCAAGAACATAATTAAATAAGCAGGCATTGCCAGAGGGGAGTGTCTGGAAAGGAAAGGAATGAAGAGATGAAAGGAAAGTGGGTGGTTAAACTATATTTTTAAAACTGAGGTTCCCAGTTATAGTATGTTTCGCACGCTCCCCCCATTTTAGCACCCCTGACAGAATTTAGTAATCTCCTCATCTTGTCCTCTACTTCAGGTCCCCTATCTGTCCTTGTACTCTCTAGGGTTTCCTTTTCTTCTTCACGACCTTCCTTCCCTGCAATTTTATAAGCTATTCCTATCCCAGTGATTTAGTTTCAGCTTATAAAACTGTGTCTTTGCCATTGTAATCAAATTGAAGGGCCTCTGCTTCATGGTTGGATTCTGTGACCAGGAGACTCTTACGAGGAGTTGGCCAGGTCTCTGTTAGGAAAGCAAAAAAGAACAATGGAGGCAATTATCCCATTGATTTCAGCTATAAATCCTATTTTGCCTGAATTGTCTGAACGATGAGTATTCTGTGAAAATGCTGCTCTCTAGTGCAATAGAACTGCAAATAATGCACATCTATTTCTTATAATCTCATCCAACATACCCACAGAGATTCAGATCTAACAAAACAGAGGTGATTTGGTTATTGAATCATAATATAAATATGGGGAAGAGGAGGGAAATTTCAAGCCTGAGGAAACTGTAGTAGGAGTAAGTATGCTGTGTTTAAGAGGTCACAGATAAAATTAATATTACCAATCCATCAATAGGCAATTACTAATAGCTTACTACACACACAGGAATAAAATGTGAAGACAGAGGAAGTGTAAAATGGAGCCGCCAACTCTACGGAGTTGTTTGCAATTTGGTCTGGTAGAAAGCTATGAAATAAGGAAGTACATGATTGAGAGCTAGAGAATGTGGCACAGGCTCTGAACCCGGACCGTTCAATGTAGTAAGCTCTAGCCACACTGGACACTTGCAATGTGGCTTGTCCAAACTGACATGTGCTTTAAGTATAAAATATAATCCAGATTTCTAAGACTTCAAAAAAAATGGAAATATCTCATTAATAATCTTAAGTTTATTACAGGTAGAAATGATAGATTAAATAAACTATATTGTCAAAATTCATTTGATCTGTTTCTACAGTATAACAAACTTACTTGTGTGGTTTGCATTTTATTTCTACTGGATAACATGGCTTTAAAAATGGTATTTTAGAGGAAGGAAAGCTTGGTAGAGAATGGACTAATCCGGATCCCTGGAAGAAATGGACCTTGAATGGGTCTTGATGACTTGGAGAGGCAGAGAGAGAAAAAGAAAAGTCAAACATAGGGAATTGGTTGATAAAATGAAGGTGAGGGGAGAAGGAACAGAGGGAGGAGAAGATCCAGTTTGAGGGATATTACAGCGAGCAGCCTGAGAAAGAAGGATAAGAAAGGAGAGAAAAAATGCAAGGGAAGTAACCCTTCAAAGCCAGTCAGAAGTTTCTGGGTTCCTCAGCAGCCAGAAAAGAAGCCGTTGAAAAGATCTGAGTAACGGAGATTCTGGATGAAAACTGAAGTTATGGAAGGGAAGTTTAGACATGGGTTATTAAACTCTTTAGCGCATTAGAAGTTTCTTATGTAATCACTAAATTCAGATCCTGAAATAATGCCACAAGAACTATACAGCTCAGCCACCCAATTCAATAAGAAGTTACAGCACAGTCTCACACATATCCAATTAACCTTGGCCTTTAGTCAACATCTGGGTTCTTTTTGTCATTTTCAAATACTATCACCCAGAGGTGCTATGATTTATATTGGGGAGGGGATTAAAAGAAAATAAGTAAGTTAGTGATAAGAAAAAGCTTTCAGATGATTCCATCTGAATTAACAGCCCTCTTTAGTTGTCTAGGAAAGAGGATGCTTTTTCTTGAAAGTGCTTTGAAATGATGATGTGCTTGTTAGTAAACATCAATTATTTTCAAATCGTAATGTTTGCAAGTTTGTCTTCCTGTAGCTCACCCTTTATGTAGGTCCAGAATATGATTGTCACAAATATCTGGGTGAGCAAGACTATGAAATGTGGTCATAAAGTAAGTGATTATTTCTAAACTCATCTTTGTCACTCGTAGTGCTTCACAAAGCACCTTTTCCTGGACTACAATTCATTTTAATTGATCCCATCAGCACTATATCTGTATCCTGAGTGACTTCACAATACCCTCTATTTCAAGAGAAACCAATCAGGTTATGGGTTTGTTAGTAATAAAAATTACCAAGGAGCAGTTTGTGGATGGTAAAAGCAATGCAAATTCTAAAGAGAAGTCATAAGAGCAATAATAAGCATCCTCCTCACTTCTTGGAAGTGAACAATTCCAAGCTCCCTGAAGCAACACTTAACCTATCATATTAAACAGTAATGGACAAATATTAGAAATGTTGATGTCAGCTTTCAGAATCTGTGGGCATCAAAACATCACTTAAGTTCTCCGAAGTATTCTCTGTCAAGTTTCCTTCTACAGTATTCTTTTCCTACTAGGACAGAGCCTTAAGCCCTAGAAGAATAATTTTGCTTGTGTGTTAATTATTTGTTTACTGGTTCATTCCAGAGTGTGAGCTGGAAAAAGGGGGAAGTGTCATAAATAGTTTTTTATGGCCCATGGTTTTTCAACTACGTCACTATTGGTAGCAGTTTCCACTGCAGGATCTATTTGCAAAGCCTAGGAAATTAGCATTAAGCAAGCTGCTAGGAAGACTTCAACAGTAACTAGGCCACAGGCCTCACACATTTTTCCTCCACCCCAGCCTCCTCTGGAGAGTACTTGCTAAACCTCTGTGACACATAATGAAGCAAAGAAAGTGATAGAACAACAGAATTACACGGGCAGATCCTTGTTTCTTCTTCTCTCTCTAAAGAATTCCTTGGACTGAAAAGCAGTTTATTTTGGAGGAGTGAGAAAGTGGTGACAGAATTAGAAGGGCCTGGGAGGGCTTCATTTTAGGAGACAGTTTTAGGCTGAAAAGAGATTTCATGAGTGTGATTTACCTGAGGTGACTTTTGGGGGCTCTTATAAAAAGGAAGTTCATGCTGAATGGGAGGTGGCTTCTGAGATGCAGATTCTGGTGAGCTAAGAGGGCTCGGTAAAGAGGAGGCAGGAGTTAAGTAGCGTGAACTATGCAGTAGCAGCCTTCTTCCCCCCTTGCTTGGGGCAGGTCATCACAACCCTTCTCAATAAAGGGGTCCAGGAACCACTAGGAATAAATGGGCATTTGCACTTCAGGTGAAACCCATTTGTCATAACTGCTTGGACTTTAAGCTTACAAATAAAAAGAACCACATATTTCCCTTTGCAGCTTGATTTAGTTAATGTCATTTTGAGAAAGAAAGAAGACATTGTTATCCCGTCCCTTTTTTTTTTTTTTTTTTTTTTTTTATGAAGAGACTGGGACTCAGAGAAGTCAAGTGATTTTCCCAGAACCAGAAAACACAGAAGTAGCAGAGCTGAGATGACTACTCCGGTCTTCTGATTCCAAATTCCAAATTCATTCTTCTAAGCGATTTCCCAAAACGGGAAATGGGTTTATCTTCTATTTATGGGAAGTGATAGTGGTATTCTATTTAGAGAACTTATATAAAATCTTACTTTAAAATAAATAATATTTCAAAAAGTAAGCTTAATTTAAAGAAAATAATCAAGAAAGTCTGGTATATTTTTACAAATATACCAAATGACCTTGCTCTAAAATACATCTACTTTCCAGCAAGCCAAAGTGAAACAATTTGAAATAAGTGGCATTTACTGACCACTCCCTAAAGTTCACACAAAAGAGGTAGTACTCTAACTTAAATATACAAGGTGAAGAAATAGCTTACTCAGCCTGTTGGGCTTCCTCTTCTACACTCTTGGGAAATGCCCTCCGTGTTAACCAAGAATTCTCAGGCCTTGGAGGGAGTTTTCCATTCTCAGTAAACTGAGATTGCAGTTGCGGAAATTAAGAGGTATCTGTCCAGCACTTCATTCCCTTAAGGTCAGGATCTGTGCTTTTAATAATGACAATTAGCTAACATATACAATTAAGCCATGCAAATGAAGTAAGAGAAAGCTAGAGGAGAAATTCAGGAGCCAGTTGCCTTTTCCAGACATCTTGTACAAATAGTGTTCAAAGGACTAATTCAAAAGATGGGATTCTTCGCTTGAACCCAGGAGGTGGAGTTTGCAGTGAGCGGAGATCGCTCCACTGCACTCCAGCCTGGGTGACAAAGTGAGACCCCATCCAAAAAAAAAAAAAAAAAAAAAAAGATGGGATTCTTTTTTAAAAAATAAATTTTACTGCGTATTTTTAAGGTATACAACGTGATGTTATAAGATGGATATAGATAGTGAAAAGGTAACTGTAGTGAAGCAAATTAACATATTCATCATCTCACATAGTTATCTTTTATTTGTTTTGTTTTGATGGGATTTTTAAGATAGTAGAAAGGAATGGTAGACAATAAACATTTGAGGGAAAGTGGGGCTTTGTAGAACTCCTAAAATGACAGCACGCACAAATGTCCCCATTATGTCTAAAGGGTAACTCGTTCCTACTTCTAGGGACAGCTGAGGGACATCAATGTAAATTTCTAAATGACTTCCTGAACTTTTTATTTTTATTTTTTGTATTTTTAGAGGAAATTATAATAACATCAAGCCACCTCTGGACCATATCGCTGCTGATATCATCAGCAAATGGCACTATTCCTAAATCCTAAGATGCACTTTTCCCTTCACATTTCAACATTTGTGAAACTCGATTGTACCTACACCTGATTTTATATACAATGCAGCCTTTCCTTTTCTTTTGTCATTGCATCTTACGCCTGATTTCTCCTTGGAATTGAGTAAATATAATGCTTACATGTGTTAATAAGAATTGAGGTCACTCATAATTTTTGAAATATGCCACCAAATATAAGCCTTTCTACATATTGTTGACTTTGAAGTCATTTCTTTTTTTAACTACTAAACAATAACACTTTTTGTTGAGAAAAATTGCATATGAACAAGAGACCAAGCAGGTAGAGAGAAAAAAACTTTTAATAATCAAGAGAATGTTACTGTGTCCCAAAGGCTAAAGTCACCTTACTATCAAGAGAGAAGGACAGGAACAGAGAGAACCAGGTAAATTACGAATTGAAAATTCCATGGTTCATTTATCTTTATTTTTAATAATTCCATTTGTGTGATTGTGTTGACCACAAGGTCATAATGTTACTCTTCATACTGACTTCTCATGTAAATTATAAGTAAGTTTTTATGCTAATGATTTATGGAGTAAGCTATTCATCTTTCCGACAGAGAGTTACCTACAAAGAAATAATTATTCTACCTCTGAGATGAAATATCATGAAAGGAGTGGTTTCCAGATATTTTGACTTTTAAAAGCTTAAAGAATATATGTAGTATAAAATTCTAAAGCAGGCAAAATTAATCCTTTTAGCAATCAAGATAGCGGCTACTTTTGGTGAGAAGGACAAGGTAGTGATAGAGAAGGGGCTCAGGGGTCTTTCCTGAAGACAGTGAGGTGGGCAATGGTATTTTCCTTGACCTGGATGGTGATTAAACAGATGTGTTTACTTTGTGATAATTGACTAGGCTGTGCACCTATGAACTGCATACTTTTCCATATATGTACTGTATTCTTATACTTAAAAAGAAGTTTAAAAATAAATGCAACAGATATAGGACTTCCTATATTACTCGTTGACCAAAAAAATGGATTCATTTTTCTTTCAGGTAAAACGTACTAGTGGTTTTAATATTATATTGACCAGGGAGTAAATGTTTACCTTAGGAACCTTAATCTTGATGTTCTCCAAAGTCATTATCTGTTCTTTCTGATTATCAGAATAGAGTATATCTCTATATAAATGAAAATTTCTGGTCATTCTCAAAAAATAACACTAAGCATGAAAATCAGAAATATTGATCTTGTTTTGTAATGATGTTTCTATTGATGTGAAGTAGTTTCTAGTAGAGTTGCTGTCCTAACACACAAATGAAATTGCACTGTTTGGAAGACACAACTGTGAATGACTTGCTTCAGTAAGGAATTTCCAACATGATGGTTTAGGGATAGAGGTGCTCGATTCCTCTGTCTCCGGTTACCCAGGTTATTGAGGACAGGGAGGTCAATAAGTAATGCCCTCCTCCCACCCATAGCACAAAACAGAGCGGGGTTCAGAGAATAGGTAAGGCTTTGGCCAGGGTGTTGAGGAGACTTACATCCCTGGGAACCAGTCAGAATGGGGGCGCTGAAAACAATGTTTTAAATTCTAGCACCCAGCAACATATGTGTGAAGATTAAATGTACTCGTGCTAAATTCACTTGCTCCATTACTGAATTTGGGTGGTGTCTGTTAAAGATGGGAACAAAGGCATTCAGGTCCTGGTATCTTCTACCACTCCCAGCATGAACAGACTCATGTCAGTGGGTAAGGGATGGTATTTCCCGAGAAGGCTTTGAACTCTTGTAGTGGGTCAAATAATGGCCCCCCACTTAAAAATGTTCATGTCCAAATCCCTGGAAGCTGTGAAAAGGGGTTTTTGCACATGTAATTAAGGCAAAGATATTGAAATTAGATCATCCTGGATTACATAGGTGGGCCCTACATTTAATGACAAGTATCCTCATAACAGAAGAGGAGAAGGTGATGTGAGATTTGGAGCAGCAGAGATTGGAGTGATGTGGCCACCAATCAAGGAAACCAAGGACTTCCAGCAGCCACCAGAAGCTGGAAGAGGCAAGGAAGGACTCTTCCCTAAAGCCTTTAAAGGAGCACAGCCCTACTAACACCTTGCTTTTGGGCTCTGGCCCGCAAAACTGTGAAAGGATACATTGCTGTTATTTGAAGCCACAGTTCGTAGTAAATTTATTACAGCAGCCCTAGAAACTGATACAACTCCTAAATACACCCTTAGCAACACTGCTCAACAAGAAGTAGGCAATTTCCTCCTGACTGAAAAATACTGATACTGTTATGGGATCCTTGGGGGTGTTGCTTTTCTGTCCAGAAACCTCTGTGGCGGTGGCACCTTTGCATGAGTTTTGCTCGGGTCCACTGGGCCCACTCATCCTGGCAGGCTGCGCTCAGCTGACACTACTGGCGTGGATCCCATGCCTCCAAAGAGACTGGAGCGAAGCGGTGAGGGATGTGTGAGGAAGTGAGCGTGGGGTCTGGCACACAGTCAGGCTCAATGGCTGCTACAGCGGGATGGGCAGCTTCAGGTGCTGGCACGGGTGCTGGCTCACTGCAAGGCTGTGGCTGCACCAAGCAGCGCAGCAACGGAACGCATTGGTGCCTGGAAACTTGGAGACTCCAGGAACCTCAGGGCTCCAAAAGGCAAATCACAGCCCTAGCTTCGGGAGCTCCCAGGTCTGGGCTGCCAAAGGGCTGCAGCTCTTCTCTCCTCTCTCTCTCTTCGCTCCTCTCCCTTTCTCTCTTCACTCCTCCCTCTTTCTCTCTTCACTCCTCCCTCTTTCTCTCTTCACTCCTCCTGTCGCCTATGAACAGCGAATTCAACCTTCCAGTTTTCAGACTAGGAATGCTGGAGTTGTCCTTGATTACTCTGAATTGTTCACTCCGCATATGGGCACTGAGGATACATTGATGAACTACACAGACAAAAAGGATAGAAATTCCTGTCAAGACTACATTCAATAGGGATGAAGCAGGCAATAATGAATAAACATACTAAGTTGAATATGACTATTTAAATATATATAACACATATGACTTGTATAATGTTAAATATTTTAAGTTTTTTAAATTCTTCCCTTCATAGATTTTACATTATAGTAGAAGAGGCATTTTTGTTGTTGTTCTTTTTGTTTTGGATTCAGAGGGTAAATGTGCGGGGTTGTTACATGGGTATATTGCATAATGCTGATGATGGTCCCATCACCCAGGTGGTAAACATAGTACGTAATAGGTGAATTTTTAGCCCGTGCTTCCCTCTCCCATCTAGTCGTCCTGAGTGTTTATCGTTGCTACGTTTATGTCAATGTGTATTCAATATTTAGCTCCCACTTATAATTGAGAATATGCAGTATTTCGTTTTTTGTTCTCGTGTTAATTTGTTTAGGATAATGGCCTACAAAGAACATGATTTCATTATTTTTATGGACATGTAGTATTTCATGGTGTATATGTACCACGGTTTCTTTATACAATCCCACTGTTGATGGGCACCTAGGTTGATTCTATTGCTGTTGTGAATAGGGCTGCAATGAACATACAAGTGCATGTATCTTTTTGGTAACAAAAATTTTATATTTGGATTACCCAGTAGAATTGCTGGGTTGAATAATAGTTTTGGTTTAAGTTCTCTGAGAAATCTCCAAACTGCTTTCCACAGTAGCTGAACTAATTTACATTTCCACTAGCAGTGTATAAGCGTTCTCTTTTCTCCACAATCTTTTCACCAGCATCTGTTATGTTTTGGCTTTTTAATAGCCTTTTGATGACTGTGAAATGGTATCTCACTGTGGTTTGGATTTCCATTTCTCTAATGATTAGTGAATGTTGAGCATTTTTTTCATATGTTTATTGGCCGTTTGTATGTCTTCTTTTGATAAGCGTCTGTTCATGTCCTTTACCCATTTTCAATTAAAATATTTGTTTTTTGCTTGCTGATTTAAGTTCTTTGTATATTCTGGAAATTAGATCTTTGTCAGATGCATAGTTTGCAAATATTTTCTCCCATTCTGTAGCCTGTTTACTCTGTTGGTAATTTCTTTTGCTGTACAGAAACTCTTTAATTAGGTCCCACTTGCCTATTTTTAGTTTTGTTGCAATTATTCTCTGGAACTTAGCCATAAATTGTTTGCCAAAGCCAACGTGGAGAAGGATATTTTCTAGGTTTTCTTCTAGGATTTTATAGTTTAAGTTTTACATTTAAATCTTTAATCCATCTTGAGTTAATTTTTGTATATGGTGAGAAGCAGGAGTCTAATTTCATTCTTCTGCATAGGGCTAGCCATTATCTTGGCACCATTTATTGAATAGAGAGTCCTTTCCTTATTGCTTATTTCTGTCAATTTTGTTGAATATCAGATCGTCGTAGGTGTATGGGTCCATTTCTGGGTTTTCTATTCTGTTCTATTTGTCTCTGTGTCTGTTTTTGTACCAGAACCATGCTGCTTGGTTACTGTAGCCTTTTAGTATAGTTTGAAATTGGGTAATGTGATGTCTCTGGCTTCGTTCTTTTTGCTTAGGATTGCTTTGGCTATTCAGGCTCCTTTTTGGTTCCATATGAATTTTAGAATATTTTTCTGATTCTGTGAAAAATGACTTGATATTTTGCTAGGGATAGCATTGGAATGGTAACTTGCTTTGGACAGTGTGGCCATTTTAATGATATTGATTATTCCAATCCATGAGCATGGAGTATTTTTATATTTATTCAGTCATCTTGATTTCTTTCAGCAGTGTTTTGTAGTTCACCCTGTAGAACATTTCACTTCCATGGTTAGATGTATTCCTATTTTGTGGCTATTGTAAATGGCATTGTATTTTTTTTATTTGGCCCTAAACTAGAATGTTATTGGTGTATAAAATTGCTACTGATTTTTGTACATTGATTTTGTATCCTTAAACTTTACTGAAGTTATTTATCAGTTCTAGGAGACTTTTGGAGAAGTCTTTAGGGTTTTCTATGTATGAAATCATATCATCAGCAAAGAGAGACAGTTTGACTTCTTCTTCTTTTTGGATGCCATTTATTTCTTTCTCTTGCCTAGTTGCTCTGACTAGGACTTCCAGGGCAATGCTGAATAGGAGTGGTGAGAGTGGGCATCCTTGTCTTGTTCCAGTACTCAAGAGAAATGCTTCCAGCATTTATCTGTTTAGTATGATGTTGGCTGTGGTTTGTCATAGGTGGATCTTATTATTCTAAGGTATATTCCTTTGATGCCTAGCCTGTCGAGGGTTTTTAATCATGAATGGATATTGAATTTTATTGAAGGTTTTTTCTGAAACTATAGAGATGATCATATGGTTTTTGTTTTTTCATTCTGTTTATGTGGTGAATCACACTTATTGATTTGTTATGTTGAACCAGCCTTGCATCCCAGGAATAAAGCCTACTTGATTGTTGTGAATTAACTTTTTGATGTGCTTCTTGATTTAGTTTGCTCATATTTTGTTGAGGATTTTCGTGTTTATGTTAATCAGAGATATTGTCCTGAAGTTTTCTTTTTTCATTGTGTCTCTGGCAGATTTTGATATCAGGATGATGCTGGCATTGTAGAATGAGTTAGGGAGGAGCCCCTCTCCTTAATATTATGGAATAGTTTCAGTAAGATTACTATCAGTTCTTCTTTGTATGCTTGGTAGAATTCAGTTGTGAATCCATCTGGTCCAGGGCTAAATTTGGTTGGTAGGTTTTTTATTACTGATTCAATTTTGGAACTTGTTATAGGTCTGTTCAAGTTTTCACTTCCGTCCTGGTTCAATCTTGGGAGGTTGTATGTTTCCAGGAATTTATCCATTTCCTCTAGATTTCCTACTTTGTGTGCATAGAGGTGTTCATAACGGTCTCTGAAAATCTTTGGCATTTCTGTGGGATTGGTCGTAATGTCATTTTTGTCATTTCTTGTGCTTTTTGGAACTTCTGTCTGTTTTTCCTCGTTTTTCTAGCTAGCAGTCTATTAGTCTTGTTTATTCTTATGAAAAACCAACTCTTTGTTTCACTAACATTTTATGGACTTTTGCATCTCAATTTTATTTAGTCATTATCTGATTTTAGTTATGTCTTTTCCTCTGCTAGCTGTGAGATTGAATTGTGCTCTTTTTTTCTAGTTCCTCTAGTGTTATGTTAGATTGTTTAGTTGAGATCTTTCTAACCTCTTGATGAAGGCATTTTAGCACTATAAACTTTCCTCTTAACACTGCTTTTGCTACATCCCAAAGATTTTGGAAAGTTGTGTCTCTATTTTCATTAATTTCAAATAATTTTTTGATTTCTGCCTTAATTTCATTGTTCACCCAACAGTTATTCGGGAGCATGTGGCTTAATTTCCATGCTTTTGTGTAGTTTTGAGAGATCTTCTTGGTATTGATTTCTATTGTTATTTCACTATGATTTGAGAGTGGCCTTTGTATGATTTTAATTTTTTTTAATTTATTGAGACTTGCTTTATGACTGAGCATGTGGGGCAATCTTAGAATACGTTCCATGTGCATATGAGAAGAATGTGTGTTCTGTCATTGTTGGCTTGAGTATCCTAGAGAGGTCTATTAGGTCCAACTGGTCAAGTGTCAAGTTTAATTCCAGAATTCCTTCGTCAGTTTTCTGCCTCAGTGATCTGTCTAATGCTATCAGTGGAGTGATAAAGCCCCCACTAATATTGTGTTGCCATCTACGTTTTATTGTAGGCCAATAATTTGTTTTATGAATCTGAGTGCTCCAGTGTTGGGTGCATATATGTTTAGAATAGTTAAGTCTTTTTGTTCAATTGAACCTTTTATCATTTTATAATGCCCTTCTTTGTCCTTCCTGATTGTTGTTGGTTTAAAGTATGTTTTAATCTGATTTAAGGGTAGCAACTCCTGCTCTTTTTTGTTTTTCATTTGCATGGTAGATCTTTCTTCATTCTTTCACTTTGAGCCTGTGAGTGTCATTCATGTAGGATGCATCTTCTGAAAACAGCAGACAGTTGTGTCTTGTCTTTTTATCCAGCTTACCACTTTATGCATTTTAAAGGGAGAGTGTAGACTGTTTACATTTAGGGTTAGCATTGACATGTGAGATTTTGCTCCTGTCATTGTGTTGTTTAGCTGGTTGTTTTGTAGACTTCATTGTGTAATAAGTGTATTTTTATTGGTAGCAGGTTTCGTCTTTCATTTCCATGTTTAGCAATCACTTACGGATTTCCTGTAAGAATCATCTGGTGGTAATGAATCTCCTTGGTGCTTGCTTGTCTGAGAAGGATTGTATTTCTCCTTCACTTATGAAACTCAGTTTGGTGGGATATGAGTTCTTGGTTGAAATTTATTTTCTTTAATAATGCTGAAAATATAGGCCCCCCCATATCTTCTGGCTTGTAAGGTTTCTGCTGACAGAACTGTTGCTGGCCTGATGAGGTTCTTTTTGTAGGTGACCTGACCTTTCTCACTAGCTGCCTTAACAATTTTTTCTTTTGCATTGACCTTGGTGAATCTGATGACTATGTGACTTGGCAATGGTTGTCTTGTATAGTGTCTCACAGGAGTTCTCTGTATTTCTTGAATTTGTATGCCCACCTCTCTGGTGAGATAGGGGAAATTTTCATGGACTGCATCCTCAGATGTATGTTCTAAGTTGCTTACTCTCTTTCTCAGGAATGACTGTGAGTCATAGACTTGGTCTCTTTACATAACCTCATAAATCTTGAAGGTTTTGTTCATGTTTTAAATTCTTTTTTCTTTATTTTTGTCCAACCAAGTTGATTCAAATAACTGGTCTTCAAACTCTGAGATTCTTTCCTCAGCTTGGTCTGTTCTGCTGTTAATGCCTCTGACTATATTATGAAATTTTTGAAGTTGATCCCTCAATTTCTGAAGTTCAGTTTTGTTCTTTCTTAAAATAGCTATTTCATCTTTAAGCTCTTTGATCATTTTTCTGGATTCCTTGAGTTCCTTGTATTGGGTTTCAATGATCTCCTGGATCTTGATGTACTTCCTTGCCATCCAGATTCTGAATTCTATGTATGTCATTTGAGTCATTTTAATCTGGTTAAAATCCTTTGCTGGAGGACTTGTGTGTTTGTCTGGAGGTAAGGAGACACCAGCTTTTTTGAATTGCTAGAGTTCTTGAGATGACTCTTTAACATATGAGGGCTGGTGTTCCATTAACAATAGTGTACATTGAGTATAGTCAGTTGGCTTCATTCTGAGTGCTTTCAAAGGGCCAAAGCTCTGTACAGCATCTTTATTTGTGGCTAGATTTTTGCTTTAGGTTTCACAGGTGCTGTATATTGGAAAAATGTTTTTGGTGTTGTCATTTGGGGTGCAATCCAGTAGGTGATGCTTAAGAGTGGTAGCTGGCAGATAGGCTCTTACTCAGTCCACAGCTCTTTTGTATTTTGGTGCAGTCCTCAGTAGTGCTCTGTGGTGGTAGGGAGAGATGACCCCCTCACCAGATACATTCCTGGGCCTTGGGGGAGCCCTCTCTTATTACTGGCACTGCACCTGCATTTCATTTATTAGGTGTCCTGGGCTGCAGGGTGCCCTCAGGCAGAGGCTGCGGCTGGAAAATAGACCATACCCTTCCCTGGCTGGCCCTGCACAAGGAGGCACACCCTGTTCCTGAGCCAGTCCATGAACCCAGCTGTCTCACCCCTCTCAGTGTTCTGAGAGTAGGGGATCCCCCACTGCTTGAGCACCATGAGCCCCTCCTGGCTACAGGCAGTGGGGGTAGGTATAGTCTCTCAACCCACTGTCCAACTGATTTCCAGGGTAACAGAGAGCTGTGCCTGCCCACAGAGTTCAGGCAGAGGCCAGGCCATTGTGCTGGAAGCCGATGCTAAGCCTTGTCTGATGATGGGGAGTGAAGCAATGTAACGGCTCCCTAACTGTGGCTTCTCTCAGGGCTATGGCAGCTGGCATGAGACTGCTCCAGGTCCAAGGCCTGTGGGACTTCCTGTGGACTTGAGTTTTGCCTCTGCAAACACTCCAGCAACTCTCTATGTCAGTCTAGAGGCCCAGGGACACGGATCAGGTATTGGGATGAGGGGGTTCTCCAGTTCCCAGGATTTCACAGGTCCCTGTGGAAAGTGAGGATCCCCCAGGGGCTCTCACTCACTCACCCTTTCTCTATGTTGGGGAGCTTCCCCTGGCTCCATGCCCATCTTGGGTGGCCAGCTGCCCAGCTTCACTCTTCCCTGTTCTCTGTGTCCCCTCACTCCCTTAATTGTCCTGATATCGTTCCTTAGGTGATCTACTTGCAGAGGCAGTGTTTACTCGCCACTTTGTTTTCTCTCTGTGAGAGTAGCACACACTAGCTGCTACTCATCTAGCATCTTGAATTCTTCCCATCTGAAAAAGTTTCAACTGCAATCACAGTTAAAGAAATACAAAAACAATAGCACTCTAAGTTACAACTTCTCACCTATAGAATTCAAAAACATCCAAATGATTAACTAAACATTTGTTTGGTAGATCTGTGGGAAAACATGAATTCCTTGTGAATTACTGGAGAAAATGAAAATGATGCAACACTTATGGAAGAAAATTTGGGGATTTTTGGGGGGGAGGGGAACAATATATTTAAAACTATAAATGCATTTATCCTAGCAATTCTATGAATGGGGATTTATCTTAGGGTACACCTGCACACTTAGGAAATAATGTATGCAGTCATTCATTACAGAATTGTTTGTAATAGCAACAACCTGAAAAGCAACTCATATATCCATCCATCACACAGGGACTGGTTTCATGACTACTGTTCATGAATACTCTGCAGCCCTTAGAAAGAATGAGGAAGTGGCCGGGCACGGTGGCTCATGCCTGTAATCCCAGCACTTTGGGAGGCCGAGGCGGGTGGATCACGAGGTCAGGAGATCAAGACCATCCTGGCTAACACGGTGAAACCCCGTCTCTACTAAAAACAATACAAAAAAATTAGCCAGGCAGGCGCCTATAGTCCCAGCTATTCGGGAGGCTGAGGCCGGAGAATGGCATGAACCCGGGAGGCAGAGCTTGCAGTGAGCCGAGATAACGCCACTGCACTCCATCCAGCCTGGGCGACAGAGCGAGACTCCGTCAAAAAAAAAAAAAAAGAGGAAGTTCTCTATGCGCTGACATGGAAGGAAGACAGATGGTTGAATGAAAAAAGTACATAATTAGCCATAAAGTGTAAGACTTTTTGTCTAAAAAAGAAGGGTGATATAATTGCATATTTATATTTTCTTCCATTTATATTAAGAGATAATAAAGGTACACAAATTGGCTAGAATAAAGTGGTTTCCTATAAAGGGTAAGAGTAATTGAGTGGATGAAGACTAGGGTTAGGGATAGATTTCTCAGTGTATTCATTTTAATATATGTATTCATTTTATATATGTACTAATTTTTATATATGTATTTATTTTATATTTTGATTTTCTTAACATAAATATATTATTCCTTCATAAAATTAAACTTGATACATTTTTGATTACTAGATATGTAGAAAGCATTATGTTCAGTACCACAGTAATACTTTCAAACCAGCTACAATTAGTATTTATGAGCATCTATGTGCCAGACATTGTGTTCTGCTTTGGTTGGTGGGGGTAGAGGAGGAAAGGAAACCATGGCTTACATAGGAGTGGAAGTCTTGTCTTTCACTTTGCACCTCTCTCCTTCAGACCTAGCATAAATATGACCTTAGGGGAGGCAGAACACATATGATAAAGAGATAACTAGCAAGAGACATAATAGTAGCTAAATAAATACTGAAGGAAAAATTCAGGAAGAGGTAGGAAGGATATGCCTCATCACTTCCACCTGTTAAGAAAAACTTTAGACATTCTTGCCAATATTCCTTATTGCCTGTCTTTTGAACAAATGCCATTATCACTAGAGTGAAATGATATTTCATTGTAGTTTTGATTTGCATTTCTCTCATGATCGGTGATGTTGAGCACCTTTTTATATACCTGTTTGCCATTTGTATGTCTTCTCTTGAAAAATGTCTATTCAGATCTTTGCCCATTTTTAAATGGCGTAATACATTTTTTTCCTATTGAGTTGTTTGAGTTCTTTATATATTCTGGTTATTAATCCCTTGTCAGATGAATAATTTGCAAATATTTTCTCCCATTCTGAGGATTACCAGAGGCTCAGAGGGGTAATGGTGGTGGGGGAGAATAAAAATGGTTAATGAGTACAAAAATATAGATAGGAGTAATAAGATCTAGTATCTGATAGCACAACAGGGTAATTACAGCCAACAAAAATTTATTGTGCATTTCAAAATAACTAAGAGTATAATTGGAATGTCTGTAACACAAAGAAGCAATAAATGCTTGAGGTGATGTGAGGGGATGGATATCTAATTTACCTTGATGTGATTATTACATATTGTATGCCTGCATCAAAATAGCTCATGTATCTTATAAGTATATACACCTATTATGTACCCATTAAATTTTTTAAGAACTTTAAACAAATCAAATTTAACAGAGTTTAATTGGGCAAAGAATGATTTGAGGATCAGGCAACCCCCAGAAACAGAAGAGGTTCAAAGCAACTCAGTGCTGTCACATGGTTGGAGAGGATTTATGGGCAGAAAAGGGAAAGAGAGATACAGAAAATGGAAGTGAGGTACACAAACAGCTGGATTGGTTACAGCTTGCCATTTGCGTTATTTGAACATAATCTGAACAGTTGGCTGTCTTTGCTTGACCAAAACTTGGTGTTTGGTACAAGAGCAGATTACAGTCTATTTACACATCCAGTTAGTTTACAGTTCACTATACACGAAGAAGAAACCTTTAAGCAGAACTTAAAATATGCAAAGAGGAAGCTTTAAGTTAAACTTAATTTAACACACCCAATTATCAAAAAATGAGTAGCTTTGCAAAAGTGGATTTTCCTGGTCATCTTTGGTACTTCCTTAAAAAAGAGAAAAGTAGTACTCACGATAAAAAAAAAAAAGTCCTCAAGTCTTTATTTTATTCCTTTCCAATTTAAAATGTTACATCATCTGAGGAAGGTTTTTCCCTTTGACCGCTTTCACAGACATTTTTTCTGCATGGGTTGGCCAGAATCAGAAGAGTAATTGTAACTTTCTGTTCTTGTCCTACAGTTACAAAGCGGTTTCACTTTGTAAATGCTCTTTGGATGGCAGGAACCAAGCAGCCATGAAAAGAGGAGTTACACCTTTAAAGGAGTCATTCCATCATGACTCTCAGGACTGGAACATGGAATACCTGAATGGCCTCTTTGGCACAGATAGGCCACCCTTGAAAGGTGTTCCAAGCTAGGAACTCACTACCACTGTTACATCGATGCAACTCTGTGAGAAGTTCTTATCTGGTGATGGAAAATCTCATCTCTTCAACACACTGACTACTACCAGTCTCAGAACCCTGTAAACAAGATTCATTCATCTCAAATTGGGTTAAAGCAGTCACCCTGCCTTACATTAGTTTGGAATAAGGATGTGGGGATGGTGGTAGAGGAGGGGAGTGGATGATGATTTTTTATTGTTATTTGATTCTAAAGAAACTTCTATACATTTTGCATTTAAAATAATTATGTTTTTAACAATGTTTGGATTAATTCAAAATAGGATATTATATCCTATTATATTAAATATACTATTTAATCATCTTGTTGACCAAATGCAACTTAAACATGTAAAATGGTAAATAGCATAATAATTGTCTTCTAAGCCTGCACTATAAAGTATTTCAGTGGCCTCATTATTAAAGGACCAAGGTGCCCAAAGAAACAAAATTTAGTAATGATAAACAAGAGACAAACCTACTTCTTTTCCCCCAGAGTTCTGGCCACATTGAAATAAGGTGTTTGAATGCTTAATAAGAATTATTTTGGCCCACACAGTGGCTCATGCCTGTAATCTCAGCACTTTGGGATGCCAAGGTGAGCAGATCACTTGAGGCCAGGAGTTCAAGACCAGCGTGGCCAACGTGGTGAAACCCCATCTCTACTAAAAATACAAAAATTAGCCCGGTGTGGTGGTACACGCCTATAGTCCCAGCTACTCGGGAGACTGAGGTGGGAGAATCACTTGAACCCGGGAGGCCAAGGCTGCAATATCGAGATCACACCACTGCACTCTAGCCTGGGCAACAGAGTGAGAGTGAGACTCTTTCTCGGAAAAAAAAAAAAAAGAATTATTTTGAACAAAGTGCTGTCACCTAAGTTAGCAAAACTCCAAGCAAGGTTTTTGGCTCTGTAAGGAAAGAATTAGCCTACTCATTTGGAAATTTAGTGGTGTTTGTAATGCAGAAAGTGACAGTGAGACTGGAAAGGGATTGGCTTTGGGGCTTGTTCTGCTTTATAAATAATAATGAATCTTCTCCAACATGAAGTAATGTGAATTAAAAAAAAAAATCTGTCCTTAGAGTACAAAATTACTTCATAACCCAATCTGCATTTCTCCACTCCAAGCATATTTTCTGGGAGTTCTACTTAGAGAGTGAAAGCTGCTGTGTGTGTGATAATTAATTTTAACAAACACTTGGCAAACTGAGCTGGACTATGTATAAGCTACCCTAGACTAAGCATGAATTTGAACTGCACTTTTTATGGTGTTTTTTCCACAATGACATTATTTAGGCATTTAAAGTTATCTGAACTGCAATTTTTTGTTCTTTTTTTTTTAATTTGACTTTTTAAAAAAAATTATTCCTGAATAAAGAGGCAGTTTGTAAAAACTCGAGAACTGTGAGAGATAATTGGATCTTTGTGTAGCAAAACTAGAAGGGTGTTGGGTATCTGCTCTTTATCAAATGGACCACTTACTTTTCTTTTCTTTTTTGCCCTGTGTTCAGAAAACAAATGTGCATGTCTCCTGATTTATAATGTATAGTTCATTAATGGAGAAAGTGCTTGAGAATTAGATCCTAATGTCATTTCCCATGCAGCATCTTCATTCTTTTCTAAAGCACTATTTGGTAAAAACAACTGATAATCGTCAGAGGTGATCAGCAATGTTTGAGCACTATTTCCTTTTTATATCCTGCACATGGAATATGGACAGGCAAACAAATCATTTCCAAGTAAGAAAATAAATTTTGAGGGAGTTAATACTATAATTTGAAAGTAATAACCTCCTATTTATCCATCTAGTTTGTTGTTCTGTACTAAATTATTTGTGCATGTCTCTGTGTCTATAATTTATGTGAAACTTTGCACAATCTTAAATAGGACAAAATAGACATTCTGTAATTTCCCAGGCAAGCTATTTAAGGTGACTATCTCTCTACATATTTGAGATGAAAAACAATAACATGACAATCCATCCCTTCTTAGGTTTTTGTAAGCAGACTTACTACCTGTGACTCAGTTTTGTTCTCACAGGGTACTAATTAATCCTTCACGATAATAACTTGTCAAATTCCATTACTTCTGTAAAGGCAATACTTTATATTTGTTTGTATTCAAATTTTAAACTGATGTTAAATGCCGTGGGTGCAACTGCAGGTTAAAAATATGTGTTTGAATCTCTTATTCTTTTTGCTTGGCAATGTATGAAATAACTGCTCTTTCTAGAAATCTTGATGATGAAGTGGCCTGTTGTTTTGTCACCTAAAAATGCAATAATGTTCAAATTAAGCTTTTCTTTATTAACATCACTTGATTGTGTGCCATATTTAGAGCTTAGTGAAATTTTAATCTACACATTGATTAAATACATTTTATTTATTCTTGTTTCTAATGGGAACTTTCTTTGTTTCTAATGGGAAATTTCTTAAATTAAATTACATCCAACATTTATTAAAGACCTAAAACATAGGCAATTACTGTGCTTAGAGGAAAAGCGCAGACGAAAGTGAATCAGACAAGTTCCCTGCCCTCCGGAAGCTTTCAGTCTAGTGATGAGAAAGACATATACACACCTTATGTTGATTTAAAAAAAAAAAAAAAGCTCTTACCTGGTTGCTGGCATATGAAAGTGTTAGTTACAGATCTGCCCCAAACTAAAGGTGTCACCTCGAGTAAATCTCTTTCCCTTTCCCTTTCAATCTCTTCATCTATAAACTAGGGGTTGGGAATACATTTATTAACAAACACAAATTGAGCGTCTACCATGTGATAATAGTAGCTAAACTTACTGAGCAATTACCATGGGGCAGGTATCAAGATAAACCCTTTATGATGGTAACCTCATTTAATCCTCAAAGCAATTCCATTTTCAAGAGGAGGAATTTGAGGCTCAAAAATGTTAAGTAACTCCCCCAAGGATGCAAAGTGATTGAGCCAGAATTCAAGACTAGGTTGGTTTGACTCCAAAACTCATGCCATTAAACCCTATTGTGTCACTGCAAACAACTCTAATAGTTTCAAATTATTAGTTCTATTAATATTATATTAGCATTATTTGCCCCCAAAATGTAAAATGTAAATACAAAGAGTTTGGTTTTTGTATTACTAGTGGAGGTTAAAGGTGCACAATGGAATTATTCAAACTGGGAAAATCCAGGAAGACTTCATGGAGGAGGCAGCATATGGCTGCAGTTAATAAGGTTTGCTCACACAAAATGGAGAGGTGAGGACATTTCAGGCAGAGAGAATTATATGAGAGGTTACAGAGCAGTAAACAGTCATGCGTCTGCAAGATCAAAGGGAAAGGGCGGTAAGAGAGAAGCTTGAAAGTCAAGTGGAGCCAGATTGTGGAAAAACTAGAGAGTCATGCCAAGGACCTTGACATATAGAAAATGGGAAGCCCCTGAAAGGTGAAGAACATGAGAGTGAAATGATTAGTAACTTTTTGGTTTAGGACTTGTTTCTTTTGTGTTTTGGTTGCTTTCTTGTTTTGTTTTGTTTGTGGTTTTTAAATTTACAACCAATAAGAATATTTAGTAAGGTTTCCAAATACATCATGAATATATAAAACTAGCCTGACTCAAGGATAATAATTCTGGGTAGTTGGAGTGAAGTTTCAATCAGCTACGTGGCATTTGCTAATCATCTGATATGAGCTAACAATAAAGGAGTTAACAAATAAACTGTCAGCCTACAGTCCAGGGTCTCAAATAGCATGTGACATAGTTGAGAAGCAGTTTTCCATATCATACATGAAATAACTAAAGAAACTACTTACAAAGCACTATACCAGTAACTACAATAAAATACAACTATACATGCAAAATAATGCTGAAAGCTGCAAGTAGAGGGGTAAAGCTAGGCCGGTTGCTCAGGGAACCATTCTGAAGTGGATTTGGGAAGTATGTCTAGAAGGGGAGCCATTGCTGTGAGAGTGCTGAGGCTCATCTGCTACTAGTCCCCCACTACTCAGGCATATGGTAGGTCAGTAACAAAACCATCATTGTGCACTGTTCTTTCCATCTAAATTCCATCAAATTATGACCAACCTATCAAGGTACTAGTTCAAATTCTCTCTTCCTCTATAAGCTAGTGGTCTTCTCTAAAATTTAAGAAGATCGTGCTCATCTTCCTACTTCTTGTTCTCTTTCTTCTGTGTTTTCTGAGGCTGCAATGAACTAGGAACTTCCTCTCCCCAGAACTCTGTATTCCAGGCCTTAGATCACTCAAAACTGTTGCTTATAAAGTGCAGAGAATCAACAGAGAAGGAATAGAGGTTAATGTCTGGTCAAAGATGTGATTCTCTTGTTGAAAAGTTCATTAGCTTATTATTTATAGAATCATAAGTCCCAGGAAAAACCAAAAGGAAATATATATTGGATCCTAATGATATTCTCTTTTTTTCTTTTTTCTTTTCCCCCACTCCATTGCCCAGGCTGGAGTGCAGTGGCATAATCTCAGCTCACTGCAACCTCCACCTCCCGGGTTCAAGGGACTCTCCTGCCTCAGCCTTCCAAGTAGATGGGATTACAGGCATGTGCCACCACATCTGGCTAATTTTTTTTTGTATTTTTAGTAGAGATGGGGTTTCACCATGTTAGTCAGGCTGGTGTTGAACTCCTGACCTCAAATGATCCACCAGCCTCGGCCTCCCAGTGTGCTGGGATTGCAGGCGTGAGCCACCACACCCGGCCTGATATTCTCTTGCAAGGGCATTGTTTACATTGTCTATCATCAGAACTGTAGAGTGTTGGCTCCAGGCACAGAACCCCTAGAGTTTTGTAAACCATTTATATCACACTGGCAACCAGAAGTAACTTTATATACTCAAGAATCAAGATTTCACCTAGAAGTACCTCAGGTAGGTGTTGGTTCATTCACATTCCAACCAAAAGATAATGTACCATAAAGTGCATACCGCCTAGTCTGTAATGATTGAGGCAACCACATAAAATCTCATTATTTAAAAGAAATTAAGTCCAGGCACGGTGGCTCACACCTGTAATCTCAGCACTTCGGGAGGCCAAGGAGGGCAGATCACCTGAGGTTGGGAGTTTGAGACCAGCCTGATCAACATGGAGAAATCCCATCTCTACTAAAAATACAAAATTAGCGGGGCATGGTGGTGCATGCCTATAATCCCAGCTACTCAGGAGGCTGAGGCAGGAGAATCACTTGAACCCAGGAGGTGGAGGTTGAGATCGTGCCATTGCACTCCAGCCTGGACAACAAGAGTGAAACTCTGTCTCAAAAAAGAAAAAAAGAAAAAGAAATTAAATGCACTATGGTTTATGGAGCGGTATTCCTCCTCCATGTCCTACATAAGATCTTTCACATGCCAGTCACAGTTAAATCTAATTTGCTGTAATCTGGATAAATGGGAGCTAATCAACAAGCTCTCAGCTCTAGCTCTGAATCAGCAGCAGATATTGCATTTTTGAAATACACTAATAGCGAGAATGCCTTCCTGACAACAACTGGCATTTTTGACACAGCAGGAAGGTTATCTGGATTCTGATATAATAGTTATTGGAATCATACATAGGTACATAGTTTAAAAGGCTAATAAGTCATTTGTTATTGCTTTTATTATCTCTGCATAGTTAGTAAAATTGAGATTAGAACCACTTCTCGAATGTACTGTTCTAAATCCTTAGCTTGCTTGATCACACATGACCCTCACAATGATCCTAGGAGAAATTATTCTGCATGCCATTTTGTAGCTGGGGAAACTGAGGCACAGAGAAATACAGTACTGCCCAAAATGTCATAACTAATCAAAGGCAAAGACAATACTCACACCAGCTCTGATTCCAGAGCCCACTCTCTTAACCATATGCTTTTCTGCTTCCCTAGTTGTAGAGTCTTTTTGTATGACTGCATTAATTATATGTGAAGAGTTCAAAAATTTCTATATAAGGTCTTTTAAGGGTGTCATTCTGGTTGAAAATGGAGGACTAGGCTTCTCACTTGAAGACATATTTCTGTAGAAAAACCTATTTTCATTTAGATGCTACAGTTACTTGATGTGGTTAATAAACCAGTTAACAGAGTATGAAAAGGATAAGGGTTAAAGCCCTCCCAAGCCATCTTTCATGCTGCTAATATGAATCACATTACTAGATACTTAAATATCATTTTCTCTTTGGTTCCCAGAAGACTGCATATATGCTAGAATATTTGTCCTCCTCTTTTACCCTTTCAGGCAATAAAGTATTTTGGACCACTGTACTATGTTATAATTATTGTTTCTCTCCTGATTTTTTTGCTCCAATCTAATGAAAGACATACAAGCTACTATACTGCTACACAATGACTAAATACCTGTTGGATTAGGTGGGGGGAAGATACACAGTCACTGGCTAGAAAGCATCATGCATACAGAGCCATTTTCACCATATATTTTATTTCTCATGATCATGTAGAATTTAGGCTTTGGTGTTGATTATTTCTCTCTTAGGAAACATAGTTGTTTCAGGGTTGATATCACAAAAAAACAGAAAAACCTATTCGAGAAAAGGAAAATTATTTGTCTGTAGGCCAAATTTTGAAGTAGGAAAACCTGCTTTTGGAGTTGTATTCCCCTCCCAGGCACTTAATCCAAGTTCCAGTCTTACTCTAAACTGGGGATGCTAGTATTAACCACCATAGGAGTTATCTGAGATGAGTTATCATCAACTTGGTACCAGGTTGTTGTCCTCTGGACTCAGTGAGCTCTAGAATTGCATGAAACTGGCCTAATTTATCAAAGTATGTAGCCTTGGGTAAATAATTCAAGCTCTCAGAGGTCCAGTTATCTCCTCTGTAAAACATATCTACATCCTAGGGATGACAATATCTACATCCTAGAGATGTCAGGAGGATTAAGTGTAATTTTTTTTAATTGTATGTATTTAAAATGGGCAACATAATGTTTTGATATACACGTGTATAGTGATTACTACAGTCAAGCAAATTAACATATCCATCATTTCATAGCTACCTTTTATGTATGTGATAAGATTATCTAAAATCTATTCTCTTACCAAATTTCCAGTATACAATATTGATATGGTTTGATCCATATCCCCATCCAAATCTCATGTTCAGTTGCAATCCCCAACGTTGGAGATGGAGCCTGGTTGGAGGTGATTGGATCACAGGGGTGGCTTCTAATGGTTCAGCACCATCCTTTCTTGGTACTGTATAGTGAGTAAGTTCTCACGAGATCTGGTTGTTTAAAAGTGTGTAACACCTCCCCCCCTTTCCCTCTCTCTGTTCCTCCTGCTCCCGCTATGTGAAGTGCCAGCTCCCTCTTTGCCTTCCGCCATGATTGTAAGTTCTCTGAGGCATCCCCAGAAGCTGATGCTGCCATGCTTCCTATACAGCCTGCAGAACCATGAGTCAATTAAACCTCTTTTCTTTGTAAATTACCCAGTCTCAAGTATTTCTTTATAGCAATGCAAGAATGGACTAATACAGAAAATTGTTACTGAGAAGAAGGGCATTGCTATAAAGATACCTGAAAATGTAGAAGTGACTTTGGAACCTGCTAACAGGCAGAAGTTGAAACATTTTAGAGGGCTCAGAAGAAGACAGAAAGATGAGAGAAAGTTTGGAACTCGCTAGGAACTTGTTGAGTGGTTGTAACCAAAATACTGATAGTGATATAGACAGTGAAGTCCAGGCTGAGGAGGTCTCAGATGGAAATGAGAAATTTATTGGGAATGAGTAAAGGTCAGGTTTGCTATGCTTTAGCAAAGAGCTTAGCTGCATTGTTCCTCTGTTCTAGGGATCTGTGAAATCTTAGACTTAAGAATGATGATTTAGGGTATCTGGCAGAAGAAATTTCTAAGCAGCAGAGTGTTCAAGAAGTAACCTAGCTGCTTCTAATAGCCTATGCTCATAGGCATGAGCACAGAAATGACCTGAAATTGGAACTTACACTTAAAAGGGAAGCAGAGCATAAAAGTTTGTAAATTTTGCAGCCTGGCCATGTGGTAGTAAAGAAAAGCTCGTTCTCAGGAGAGGAAGTCAAGCAGGCTGCATAAATTTGCATAACTAAAAGGAAGGCAAGGGCTGATAACCAAAACAATGGGGAGAAAGACTCATAGGACTAATAGGCATTTTATTTTATTTTATTTTTATTTTATTATTATTATACTTTAAGTTTTAGGGTACATGTGCACAATGTGCAGGTTAGTTGCATATGTATACATGTGCCATGCTGGTGTGCTGCACCCATTAACTCGTCATTTAGCATTAGGTATATCTCCTAATGCTATCCCTCCCCCCTCCCCCACCCCACAACAGTCCCCAGAGTGTGATGTTCCCCTTCCTGTGTCCATGTGTTCTCATTGTTCAATTCCCACCTATGAGTGAGAACATGTGGTGTTTGGTTTTTTGACCTTGCAATAGTTTACTGAGAATGACGATTTCCAATTTCATCCATGTCCCTACAAAGGACATGAACTCATCATTTTTTATGGCTGCATAGTATTCCATGGTGTATATGTGCCACATTTTCTTAATCCAGTCTATCACTGTTGGACATTTGGGTTGGTTCCAAGTCTTTGCTATTGTGAACAGTGCCACAATAAACATAGTGTGCATGTGTCTTTATAGCAGCAGGATTTATAGTCCTTTGGGTATATACCCAGTGATGGGATGGCTGGGTCAAATGGTATTTCTAGTTCTAGATCCCTGAGGAATCGCCACACTGACTTCCACAATGGTTGAACTAGTTTACAGTCCCACCAACAGTGTAAAAGTGTTCCTAATAGGCATTTTAGGCTTTCATGGTGGTCCCTCTCATCACAGGCCCCGAGGCCTAGGAGGACTGAATCATTTCCTGGGCCAGGCCTAGGGCCCCTGCTCCCTCTTACAGCCTTGGGACTCTGCTCCCTGAATCCCAGCTGCTCAAAGGGGCCCAGGTACTGTTACAGTAGGTAGCTAATCAGGCATGAGTGGGGTAAGAGAGAAGTCCCCACCACCCACCAGGAATGTCAGGCAACCATCAGATGATGGTCAGGCAGTTGTCATACTGCCTCTCTAAAATAGTAATTGGTTGCAGCCAGCACCAGGGAGAGGCAACTTCTCAATAGATAGAAACACCTGAAATTGGTAACTGGGCGCTTCCAATAAGATCTCAGGAACTGAGAGAGTGGGCTTAACATGCACATTAAGAGGCAAAATGGTGAAGTATGACCTTTGGGGGCATTCCACCGGAAAAGGGAAGAAAGCCTCAGGTAAGCATGTATACAACTCCAGTAAACACACTGCACACGCTCACCTTCCAAGTGCAAGCAGGGCACCATGCATGCGGCAAGCTCACCCTTAGGGAAGGACCAAGGGAAAGGGGCACAAGATGTCAGAAGTAGGCCAGTGTATAAGATCCTAGGTTCAAGGTCAAACAGGGCACTTGACCTCCAAGGTGCCCACTTGGGCCTCTTCCAAATGTACTTTCCTTTCATTCCTGTTCTAAAGCTTTTTAATAAACTTTTACTCCTGCTCTGAAACTTGTCGCAGTCTCTTTTTCTGCCTTATGCCTCTTGGTCAAATTCTTTCTTCTGAGGAGGCAAGAATTGAGGTTGCTGCAGACCCACATGGATTTGCAGCTGGTAACTCAGATAACTTTCACCAGTAAGAATACAGTTCAGGCTGCTGCTTCACAGGGTGCCAGGCATAAGCCTTGGTGGCTTCCATAAGCTGTGAAGCCGGCGGGCGCACATAATGCAAGAGTTGAGGCTTAAGAAGCTCTGCCTAGATTTTAGAGGATGTATGAAAAAGCCTGGATGTCCAGACAGAAGCCTGTTACTGGGGTGGAATCCTCATGGAGAACATCTACTAGGGAAGCAAGGAGAAGAAATGTGGGGTTGCAGCCCCCACAGAGAGTCCCCTGGGGCACTGCCTAGCAGAGCTATGACAAGAGAGCCACCGTCCTCCAGACCCCAGAATGGTAGATCCACCAACAACTTGCACCCTGCAGCCTGGAAAAGCTGCAAGCACTCAATGCTAGCCCATGAGAGCAGCTGTGGGAGATGAACCCTGGAAAACCACAGGGGTGGTTCTGCCCAAGGTTTTGGGAGCCCACTCATTGCATCAGTGTTCCCTGGGTGTGAGTCAAAGGAGATTATTTCAGAGCTTTAACATTTAATGACTGCCCGGCTGGCTTTCAGACTTGCAATGGGGCCCTATAGCCTCTTTCTTTTGGCAGATTTCTCCCTTTCGGAATGGCAGTATCTGCCCAATGCCTATACCCCCATTGTATCTTTGAAGCAATTACCTTGTTTTTGATTTTACAGGTTCATAGGTAGAAGGGACTAGCTTCGTCTCAGGTGAGACTTGGGACTTTGGACTTTTGAATGAATGCTGGATCGAGTTAAGACTTTGGGGAACTGTTGGTAAGGCACGACAGTATTTTGCAATATGAGAAGGACATTAGATTTGGGAGGGGCCAGAGTTGGAATAACATGGTTTGGATCTCTGTCCCCACCCAAATCTCATGTTCAACTGTAATCCCCAGTGTTGGAGGTTGGGCCTGGTGGGAGGTGAGTGGATTATGGGGTGGCTTCTAATGGTTTTGTACAGTCCCCTCTTGGTACTATATAGTGAGTTCTGACAAGATCTAGTTGTTTAAACGTATGTAGCACCTCCCATTTCTCTCTTCCCCCAGTTCCTGCCATGTGAAGTCTGGGGTCTCCCTATGCCTTCCATCATGATTTTAAGTTCCCTGTGGCCTGCCCAGAAGCTGATCCAGCCATGCTTCTTGTACAGCCTGCAGAACTGTGAGCCATTAAACTTTTCTTTATAAATTACCCAGTTTCAGTTATTTCTTTATAGCAGTGTAAGAATGGACTAACACAATTATTAACGCTAGTCCTCATGTTGTACATTAAATCTCTAGATGTATTAGACGTAACTGCAACTTTGTACCCTACCCTACAATTTTCTTTCCCCCCAAGCCCCCCAACCAAGGGTCTACTCTGTTTCTATAAATTCAGTTGTTTTTTAATTCCACGTATAAGTGAAGTACAACTCAGTGTAGAAACTTGGTAAATGCTAGCTACTTGTTATAAGCTGTCAGTCAAAATAAAAATACAGAGATGAATCTCTAAATTAAGTGATTTATTTGGGAAGAAAGAATTGCAATTAGGGCATACATGTAGATCAGATGGTCTTCGGTATATCCACACAACAAAGAAAAGGGGGAGGTTTTGCTAAAAAAGAGAAATGTTACATAGTGCTCTTTGAGAAAATTCATTGGCACTATTAAGGATCTGAGGAGCTGGTGAGTTTCAACTGGTGAGTGATGGTGGTAGATAAAATTAGAGCTGCAGCAGGTCATTTTAGCAACTATTAGATAAAACTGGTCTCAGGTCACAACGGGCAGTTGCAGCAGCTGGACTTGGAGAGAATTACACTGTGGGAGCAGTGTCATTTGTCCTAAGTGCTTTTCTACCCCCTACCCCCACTATTTTAGTTGGGTATAAAAAGAATGACCCAATTTGTATGATCAACTTTCACAAAGCATAGAACAGTAGGAAAAGGGTCTGTTTCTGCAGAAGGTGTAGACGTTGAGAGCCATTTTGTGTATTTATTCCTCCCTTTCTTCCTCGGTGAATGATTAAAACGTTCTGTGTGATTTTTAGTGATGAAAAAGATTAAATGCTACTCACTGTAATAAGTGCCATCTCACACTTGCAGATCAAAAGGCACACAGTTTAAAAAACCTTTGTTTTTTTACACATCTGAGTGGTGTAAATGCTACTCATCTGTAGTAAGTGGAATCTATACACCTGCAGACCAAAAGACGCAAGGTTTCAAAAATCTTTGTGTTTTTTACACATCAAACAGAATGGTACGTTTTTCAAAAGTTAAAAAAAAACAACTCATCCACATATTGCAACTAGCAAAAATGACATTCCCCAGTGTGAAAATCATGCTTGAGAGAATTCTTACATGTAAAGGCAAAATTGCGATGACTTTGCAGGGGACCGTGGGATTCCCGCCCGCAGTGCCGGAGCTGTCCCCTACCAGGGTTTGCAGTGGAGTTTTGAATGCACTTAACAGTGTCTTACGGTAAAAACAAAATTTCATCCACCAATTATTTGTTGAGCGCCCACTGCCTACCAAGCACAAACAAAACCATTCAAAACCACGAAATCGTCTTCACTTTCTCCGGATCCAGCAGCCTCCCCTATTAAGGTTCGCACACGCTATTGCGCCAACGCTCCTCCAGAGCGGGTCTTAAGATAAAAGAACAGGACAAGTTGCCCCGCCCCATTTCGCTAGCCTCGTGAGAAAACGTCATCGCACATAGAAAACAGACAGACGTAACCTACGGTGTCCCGCTAGGAAAGAGAGGTGCGTCAAACAGCGACAAGTTCCGCCCACGTAAAAGATGACGCTTGGTGTGTCAGCCGTCCCTGCTGCCCGGTTGCTTCTCTTTTGGGGGCGGGGTCTAGCAAGAGCAGGTGTGGGTTTAGGAGGTGTGTGTTTTTGTTTTTCCCACCCTCTCTCCCCACTACTTGCTCTCACAGTACTCGCTGAGGGTGAACAAGAAAAGACCTGATAAAGATTAACCAGAAGAAAACAAGGAGGGAAACAACCGCAGCCTGTAGCAAGCTCTGGAACTCAGGAGTCGCGCGCTAGGGGCCGGGGCCGGGGCCGGGCCGGGGCCGGGGCCGGGGCCGGGGCCGGGGCCGGGCCGGGGCCGGGGCCGGGGCCGGGGCCGGGGCCGGGGCCGGGGCCGGGGCCGGGGCCGGGGCCGGGGCCGGGGCCGGGGCCGGGGCCGGGGCCGGGGCCGGGCCGGGGCCGGGGCC**C**GGGGCCGGGGCCGGGGCCGGGGCCGGGGCCGGGGCCGGGGCCGGGGCCGGGCCGGGGCCGGGGCCGGGGCCGGGGCCGGGGCCGGGGCCGGGCCGGGCCGGGGCCGGGGCCGGGGCCGGGGCCGGGGCCGGGGCCGGGGCCGGGGCCGGGGCCGGGGCCGGGGCCGGGGCCGGGGCCGGGCCGGGGCCGGGGCCGGGGCCGGGGCCGGGGCCGGGGCCGGGGCCGGGGCCGGGGCCGGGGCCGGGGCCGGGGCCGGGGCCGGGGCCGGGGCCGGGGCCGGGGCCGGGGCCGGGGCCGGGGCCGGGGCCGGGGCCGGGGCCGGGGCCGGGGCCGGGGCCGGGGCCGGGGCCGGGGCCGGGGCCGGGGCCGGGGCCGGGGCCGGGGCCGGGGGCCGGGGCCGGGGCCGGGGCCGGGGCCGGGGCCGGGGCCGGGGCCGGGGCCGGGGCCGGGGCCGGGGCCGGGGCCGGGGCCGGGGCCGGGGCCGGGGCCGGGGCCGGGGCCGGGGCCGGGGCCGGGGCCGGGGCCGGGCCGGGGCCGGGGCCGGGGCCGGGGCCGGGGCCGGGGCCGGGGCCGGGGCCGGGCCGGGGCCGGGGCCGGGGCCGGGGCCGGGGCCGGGGCCGGGGGCCGGGGCCGGGCCGGGGCCGGGGCCGGGGCCGGGGCCGGGGCCGGGGCCGGGGGCCGGGGCCGGGGCCGGGGCCGGGGCCGGGGCCGGGGCCGGGGCCGGGGCCGGGCCGGGGCCGGGGCCGGGGCCGGGGCCGGGGCCGGGGCCGGGCCGGGGCCGGGGCCGGGGCCGGGGCCGGGGCCGGGGCCGGGGCCGGGGCCGGGGCCGGGGCCGGGGCCGGGGCCGGGGCCGGGGCCGGGGCCGGGGCCGGGGCCGGGGCCGGGGCCGGGGCCGGGGCCGGGGCCGGGGCCGGGGCCGGGGCCGGGGCCGGGGCCGGGGCCGGGGGCCGGGGCCGGGGCCGGGGCCGGGGCCGGGGCCGGGGCCGGGGCCGGGGCCGGGCCGGGGCCGGGGCCGGGGCCGGGGCCGGGGCCGGGGCCGGGGCCGGGGCCGGGGCCGGGGCCGGGGCCGGGGCCGGGGCCGGGGCCGGGGCCGGGGCCGGGGCCGGGGCCGGGGCCGGGGCCGGGGCCGGGGCCGGGGCCGGGGCCGGGGCCGGGGCCGGGGCCGGGGCCGGGGCCGGGGCCGGGGCCGGGGCCGGGGCCGGGGCCGGGGCCGGGGCCGGGGCCGGGGCCGGGGCCGGGGCCGGGGCCGGGGCCGGGGCCGGGGCCGGGGCCGGGGCCGGGGCCGGGGCCGGGGCCGGGGCCGGGGCCGGGGCCGGGGCCGGGGCCGGGGCCGGGGCCGGGGCCGGGGCCGGGGCCGGGGCCGGGGCCGGGGCCGGGGCCGGGGCCGGGGCCGGGGCCGGGGCCGGGGCCGGGGCCGGGGCCGGGGCCGGGGCCGGGGCCGGGGCCGGGGCCGGGGCCGGGGCCGGGGCCGGGGCCGGGGCCGGGGCCGGGGCCGGGGCCGGGGCCGGGGCCGGGGCCGGGGCCGGGGCCGGGGCCGGGGGCCGGGGCCGGGGCCGGGGCCGGGGCCGGGGCCGGGGCCGGGGCCGGGGCCGGGGCCGGGGCCGGGGCCGGGGCCGGGGCCGGGGCCGGGGCCGGGGCC**C**GGGGCCGGGGCCGGGGCCGGGGCCGGGGCCGGGGCCGGGGCCGGGCCGGGGCCGGGGCCGGGGCCGGGGCCGGGGCCGGGGCCGGGGCCGGGGCCGGGGCCGGGGCCGGGGCCGGGGCCGGGGCCGGGGCCGGGGCGGGGCCGGGGCCGGGGCCGGGGCCGGGGCCGGGGCCGGGGCCGGGGCCGGGCCGGGGCCGGGGCCGGGGCCGGGGCCGGGGCCGGGGCCGGGGCCGGGGCCGGGGCCGGGGCCGGGGCCGGGGCCGGGGCCGGGGCCGGGGCCGGGGCCGGGGCCGGGGCCGGGGCCGGGGCCGGGGCCGGGGCCGGGGCCGGGGCCGGGGCCGGGGCCGGGGCCGGGGCCGGGCCGGGGCCGGGGCCGGGGCCGGGGCCGGGGCCGGGGCCGGGGCCGGGGCCGGGGCCGGGGCCGGGGCCGGGGCCGGGGCCGGGGCCGGGGCCGGGGCCGGGGCCGGGGCCGGGGCCGGGGCCGGGGCCGGGGCCGGGGCCGGGGCCGGGGCCGGGGCCGGGGCCGGGCCGGGGCCGGGGCCGGGGCCGGGGCCGGGGGCCGGGGCCGGGGCCGGGGCCGGGGCCGGGGCCGGGGCCGGGCCGGGGCCGGGGCCGGGGCCGGGCCGGGGCCGGGGCCGGGCCGGGGCCGGGGCCGGGGCCGGGGCCGGGGCCGGGGCCGGGGCCGGGGCCGGGGCCGGGGCCGGGGCCGGGGCCGGGGCCGGGGCCGGGGCCGGGGCCGGGGCCGGGGCCGGGCCGGGGCCGGGGCCGGGGGCCGGGGCCGGGGCCGGGGCCGGGGCCGGGGCGGGGCCGGGCCGGGGCCGGGGCCGGGGCCGGGGCCGGGGCCGGGGCCGGGGCCGGGGCCGGGGCCGGGGCCGGGGCCGGGGCCGGGGCCGGGGCCGGGGCCGGGGCCGGGGCCGGGGCCGGGGCCGGGGCCGGGGCCGGGGCCGGGGCCGGGGCCGGGGCCGGGGCCGGGGCCGGGGCCGGGGCCGGGGCCGGGGCCGGGGCCGGGGCCGGGGCCGGGGCCGGGGCCGGGGCCGGGGCCGGGGCCGGGGCCGGGGCCGGGGCCGGGGCCGGGGCCGGGGCCGGGGCCGGGGCCGGGGCCGGGGCCGGGGCCGGGGCCGGGGCCGGGGCCGGGGCCGGGGCCGGGGCCGGGGCCGGGGCCGGGGCCGGGGCCGGGCCGGGGCCGGGCCGGGGCCGGGGCCGGGCCGGGGCCGGGGCCGGGGCCGGGGCCGGGCCGGGGCCGGGGCCGGGGCCGGGGCCGGGGCCGGGGCCGGGCCGGGGCCGGGGCCGGGGCCGGGCCGGGGCCGGGGCCGGGGCCGGGGCCGGGGCCGGGGCCGGGGCCGGGGCCGGGGCCGGGGCCGGGGCCGGGGCCGGGGCCGGGGCCGGGGCCGGGGCCGGGGCCGGGGCCGGGGCCGGGGCCGGGGCCGGGGCCGGGGCCGGGGCCGGGGCCGGGGCCGGGGGCCGGGGCCGGGGCCGGGGCCGGGGCCGGGGCCGGGGCCGGGGCCGGGCCGGGGCCGGGGCCGGGGCCGGGGCCGGGGCCGGGGCCGGGGCCGGGGCCGGGGCCGGGGCCGGGGCCGGGGCCGGGGCCGGGGCCGGGGCCGGGGGCCGGGGCCGGGGCCGGGGCCGGGGCCGGGGCCGGGGCCGGGGCCGGGGCCGGGGCGGGGCCGGGGCCGGGGCCGGGGCCGGGGCCGGGGCCGGGGCCGGGGCCGGGGCCGGGGCCGGGGCCGGGGCCGGGGCCGGGGCCGGGGCCGGGGCCGGGGCCGGGGCCGGGGCCGGGGCCGGGGCCGGGGCCGGGGCCGGGGCCGGGGCCGGGGCCGGGGCCGGGGCCGGGGCCGGGGCCGGGGCCGGGGCCGGGGCCGGGGCCGGGGGCCGGGGCCGGGGCCGGGGCCGGGGCCGGGGCCGGGGCCGGGGCCGGGGCCGGGGCCGGGGCCGGGGCCGGGGCCGGGGCCGGGGCCGGGGCCGGGGCCGGGGCCGGGGCCGGGGCCGGGGCCGGGGCCGGGGCCGGGGCCGGGGCCGGGGCCGGGGCCGGGGCCGGGGCCGGGGCCGGGGCCGGGGCCGGGGCCGGGGCCGGGGCCGGGGCCGGGGCCGGGGCCGGGGCCGGGGCCGGGGCCGGGGCCGGGGCCGGGGCCGGGGCCGGGGCCGGGGCCGGGGCCGGGGCCGGGGCCGGGGCCGGGGCCGGGGCCGGGGCCGGGGCCGGGGCCGGGGCCGGGCCGGGGCCGGGGCCGGGGCCGGGGCCGGGGCCGGGGCCGGGGCCGGGCCGGGGCCGGGGCCGGGGCCGGGGCCGGGGCCGGGGCCGGGGCCGGGGCCGGGCCGGGGCCGGGCCGGGGCCGGGGCCGGGGCCGGGGCCGGGGCCGGGGCCGGGGCCGGGGCCGGGGCCGGGCCGGGGCCGGGGCCGGGGCCGGGGCCGGGGCCGGGGCCGGGGCCGGGGCCGGGGCCGGGGCCGGGGCCGGGGCCGGGGCCGGGGCCGGGGCCGGGGCCGGGGCCGGGGCCGGGGCCGGGGCCGGGGCCGGGGCCGGGGCCGGGGCCGGGGCCGGGGCCGGGGCCGGGGCGGGCCCGGGGGCGGGCCCGGGGGCGGGCCCGGGGCGGGGCTGCGGTTGCGGTGCCTGCGCCCGCGGCGGCGGAGGCGCAGGCGGTGGCGAGTGGGTGAGTGAGGAGGCGGCATCCTGGCGGGTGGCTGTTTGGGGTTCGGCTGCCGGGAAGAGGCGCGGGTAGAAGCGGGGGCTCTCCTCAGAGCTCGACGCATTTTTACTTTCCCTCTCATTTCTCTGACCGAAGCTGGGTGTCGGGCTTTCGCCTCTAGCGACTGGTGGAATTGCCTGCATCCGGGCCCCGGGCTTCCCGGCGGCGGCGGCGGCGGCGGCGGCGCAGGGACAAGGGATGGGGATCTGGCCTCTTCCTTGCTTTCCCGCCCTCAGTACCCGAGCTGTCTCCTTCCCGGGGACCCGCTGGGAGCGCTGCCGCTGCGGGCTCGAGAAAAGGGAGCCTCGGGTACTGAGAGGCCTCGCCTGGGGGAAGGCCGGAGGGTGGGCGGCGCGCGGCTTCTGCGGACCAAGTCGGGGTTCGCTAGGAACCCGAGACGGTCCCTGCCGGCGAGGAGATCATGCGGGATGAGATGGGGGTGTGGAGACGCCTGCACAATTTCAGCCCAAGCTTCTAGAGAGTGGTGATGACTTGCATATGAGGGCAGCAATGCAAGTCGGTGTGCTCCCCATTCTGTGGGACATGACCTGGTTGCTTCACAGCTCCGAGATGACACAGACTTGCTTAAAGGAAGTGACTATTGTGACTTGGGCATCACTTGACTGATGGTAATCAGTTGTCTAAAGAAGTGCACAGATTACATGTCCGTGTGCTCATTGGGTCTATCTGGCCGCGTTGAACACCACCAGGCTTTGTATTCAGAAACAGGAGGGAGGTCCTGCACTTTCCCAGGAGGGGTGGCCCTTTCAGATGCAATCGAGATTGTTAGGCTCTGGGAGAGTAGTTGCCTGGTTGTGGCAGTTGGTAAATTTCTATTCAAACAGTTGCCATGCACCAGTTGTTCACAACAAGGGTACGTAATCTGTCTGGCATTACTTCTACTTTTGTACAAAGGATCAAAAAAAAAAAAAAGATACTGTTAAGATATGATTTTTCTCAGACTTTGGGAAACTTTTAACATAATCTGTGAATATCACAGAAACAAGACTATCATATAGGGGATATTAATAACCTGGAGTCAGAATACTTGAAATACGGTGTCATTTGACACGGGCATTGTTGTCACCACCTCTGCCAAGGCCTGCCACTTTAGGAAAACCCTGAATCAGTTGGAAACTGCTACATGCTGATAGTACATCTGAAACAAGAACGAGAGTAATTACCACATTCCAGATTGTTCACTAAGCCAGCATTTACCTGCTCCAGGAAAAAATTACAAGCACCTTATGAAGTTGATAAAATATTTTGTTTGGCTATGTTGGCACTCCACAATTTGCTTTCAGAGAAACAAAGTAAACCAAGGAGGACTTCTGTTTTTCAAGTCTGCCCTCGGGTTCTATTCTACGTTAATTAGATAGTTCCCAGGAGGACTAGGTTAGCCTACCTATTGTCTGAGAAACTTGGAACTGTGAGAAATGGCCAGATAGTGATATGAACTTCACCTTCCAGTCTTCCCTGATGTTGAAGATTGAGAAAGTGTTGTGAACTTTCTGGTACTGTAAACAGTTCACTGTCCTTGAAGTGGTCCTGGGCAGCTCCTGTTGTGGAAAGTGGACGGTTTAGGATCCTGCTTCTCTTTGGGCTGGGAGAAAATAAACAGCATGGTTACAAGTATTGAGAGCCAGGTTGGAGAAGGTGGCTTACACCTGTAATGCCAGAGCTTTGGGAGGCGGAGGCAAGAGGATCACTTGAAGCCAGGAGTTCAAGCTCAACCTGGGCAACGTAGACCCTGTCTCTACAAAAAATTAAAAACTTAGCCGGGCGTGGTGATGTGCACCTGTAGTCCTAGCTACTTGGGAGGCTGAGGCAGGAGGGTCATTTGAGCCCAAGAGTTTGAAGTTACCGAGAGCTATGATCCTGCCAGTGCATTCCAGCCTGGATGACAAAACGAGACCCTGTCTCTAAAAAACAAGAAGTGAGGGCTTTATGATTGTAGAATTTTCACTACAATAGCAGTGGACCAACCACCTTTCTAAATACCAATCAGGGAAGAGATGGTTGATTTTTTAACAGACGTTTAAAGAAAAAGCAAAACCTCAAACTTAGCACTCTACTAACAGTTTTAGCAGATGTTAATTAATGTAATCATGTCTGCATGTATGGGATTATTTCCAGAAAGTGTATTGGGAAACCTCTCATGAACCCTGTGAGCAAGCCACCGTCTCACTCAATTTGAATCTTGGCTTCCCTCAAAAGACTGGCTAATGTTTGGTAACTCTCTGGAGTAGACAGCACTACATGTACGTAAGATAGGTACATAAACAACTATTGGTTTTGAGCTGATTTTTTTCAGCTGCATTTGCATGTATGGATTTTTCTCACCAAAGACGATGACTTCAAGTATTAGTAAAATAATTGTACAGCTCTCCTGATTATACTTCTCTGTGACATTTCATTTCCCAGGCTATTTCTTTTGGTAGGATTTAAAACTAAGCAATTCAGTATGATCTTTGTCCTTCATTTTCTTTCTTATTCTTTTTGTTTGTTTGTTTGTTTGTTTGTTTTTTTCTTGAGGCAGAGTCTCTCTCTGTCGCCCAGGCTGGAGTGCAGTGGCGCCATCTCAGCTCATTGCAACCTCTGCCACCTCCGGGTTCAAGAGATTCTCCTGCCTCAGCCTCCCGAGTAGCTGGGATTACAGGTGTCCACCACCACACCCGGCTAATTTTTTGTATTTTTAGTAGAGGTGGGGTTTCACCATGTTGGCCAGGCTGGTCTTGAGCTCCTGACCTCAGGTGATCCACCTGCCTCGGCCTACCAAAGAGCTGGGATAACAGGTGTGACCCACCATGCCCGGCCCATTTTTTTTTTCTTATTCTGTTAGGAGTGAGAGTGTAACTAGCAGTATAATAGTTCAATTTTCACAACGTGGTAAAAGTTTCCCTATAATTCAATCAGATTTTGCTCCAGGGTTCAGTTCTGTTTTAGGAAATACTTTTATTTTCAGTTTAATGATGAAATATTAGAGTTGTAATATTGCCTTTATGATTATCCACCTTTTTAACCTAAAAGAATGAAAGAAAAATATGTTTACAATATAATTTTATGGTTGTATGTTAACTTAATTCATTATGTTGGCCTCCAGTTTGCTGTTGTTAGTTATGACAGCAGTAGTGTCATTACCATTTCAATTCAGATTACATTCCTATATTTGATCATTGTAAACTGACTGCTTACATTGTATTAAAAACAGTGGATATTTTAAAGAAGCTGTACGGCTTATATCCAGTGCTGTCTCTTAAGACTATTAAATTGATACAACATATTTAAAAGTAAATATTACCTAAATGAATTTTTGAAATTACAAATACACGTGTTAAAACTGTCGTTGTGTTCAACCATTTCTGTACATACTTAGAGTTAACTGTTTTGCCAGGCTCTGTATGCCTACTCATAATATGATAAAAGCACTCATCTAATGCTCTGTAAATAGAAGTCAGTGCTTTCCATCAGACTGAACTCTCTTGACAAGATGTGGATGAAATTCTTTAAGTAAAATTGTTTACTTTGTCATACATTTACAGATCAAATGTTAGCTCCCAAAGCAATCATATGGCAAAGATAGGTATATCATAGTTTGCCTATTAGCTGCTTTGTATTGCTATTATTATAAATAGACTTCACAGTTTTAGACTTGCTTAGGTGAAATTGCAATTCTTTTTACTTTCAGTCTTAGATAACAAGTCTTCAATTATAGTACAATCACACATTGCTTAGGAATGCATCATTAGGCGATTTTGTCATTATGCAAACATCATAGAGTGTACTTACACAAACCTAGATAGTATAGCCTTTATGTACCTAGGCCGTATGGTATAGTCTGTTGCTCCTAGGCCACAAACCTGTACAACTGTTACTGTACTGAATACTATAGACAGTTGTAACACAGTGGTAAATATTTATCTAAATATATGCAAACAGAGAAAAGGTACAGTAAAAGTATGGTATAAAAGATAATGGTATACCTGTGTAGGCCACTTACCACGAATGGAGCTTGCAGGACTAGAAGTTGCTCTGGGTGAGTCAGTGAGTGAGTGGTGAATTAATGTGAAGGCCTAGAACACTGTACACCACTGTAGACTATAAACACAGTACGCTGAAGCTACACCAAATTTATCTTAACAGTTTTTCTTCAATAAAAAATTATAACTTTTTAACTTTGTAAACTTTTTAATTTTTTAACTTTTAAAATACTTAGCTTGAAACACAAATACATTGTATAGCTATACAAAAATATTTTTTCTTTGTATCCTTATTCTAGAAGCTTTTTTCTATTTTCTATTTTAAATTTTTTTTTTTACTTGTTAGTCGTTTTTGTTAAAAACTAAAACACACACACTTTCACCTAGGCATAGACAGGATTAGGATCATCAGTATCACTCCCTTCCACCTCACTGCCTTCCACCTCCACATCTTGTCCCACTGGAAGGTTTTTAGGGGCAATAACACACATGTAGCTGTCACCTATGATAACAGTGCTTTCTGTTGAATACCTCCTGAAGGACTTGCCTGAGGCTGTTTTACATTTAACTTAAAAAAAAAAAAAGTAGAAGGAGTGCACTCCAAAATAACAATAAAAGGCATAGTATAGTGAATACATAAACCAGCAACGTAGTAGTTTATTATCAAGTGTTGTACACTGTAATAATTGTATGTGCTATACTTTAAATAACTTGCAAAATAGTACTAAGACCTTATGATGGTTACAGTGTCACTAAGGCAATAGCATATTTTCAGGTCCATTGTAATCTAATGGGACTACCATCATATATGCAGTCTACCATTGACTGAAACGTTACATGGCACATAACTGTATTTGCAAGAATGATTTGTTTTACATTAATATCACATAGGATGTACCTTTTTAGAGTGGTATGTTTATGTGGATTAAGATGTACAAGTTGAGCAAGGGGACCAAGAGCCCTGGGTTCTGTCTTGGATGTGAGCGTTTATGTTCTTCTCCTCATGTCTGTTTTCTCATTAAATTCAAAGGCTTGAACGGGCCCTATTTAGCCCTTCTGTTTTCTACGTGTTCTAAATAACTAAAGCTTTTAAATTCTAGCCATTTAGTGTAGAACTCTCTTTGCAGTGATGAAATGCTGTATTGGTTTCTTGGCTAGCATATTAAATATTTTTATCTTTGTCTTGATACTTCAATGTCGTTTTAAACATCAGGATCGGGCTTCAGTATTCTCATAACCAGAGAGTTCACTGAGGATACAGGACTGTTTGCCCATTTTTTGTTATGGCTCCAGACTTGTGGTATTTCCATGTCTTTTTTTTTTTTTTTTTTTTTTTTTGACCTTTTAGCGGCTTTAAAGTATTTCTGTTGTTAGGTGTTGTATTACTTTTCTAAGATTACTTAACAAAGCACCACAAACTGAGTGGCTTTAAACAACAGCAATTTATTCTCTCACAATTCTAGAAGCTAGAAGTCCGAAATCAAAGTGTTGACAGGGGCATGATCTTCAAGAGAGAAGACTCTTTCCTTGCCTCTTCCTGGCTTCTGGTGGTTACCAGCAATCCTGAGTGTTCCTTTCTTGCCTTGTAGTTTCAACAATCCAGTATCTGCCTTTTGTCTTCACATGGCTGTCTACCATTTGTCTCTGTGTCTCCAAATCTCTCTCCTTATAAACACAGCAGTTATTGGATTAGGCCCCACTCTAATCCAGTATGACCCCATTTTAACATGATTACACTTATTTCTAGATAAGGTCACATTCACGTACACCAAGGGTTAGGAATTGAACATATCTTTTTGGGGGACACAATTCAACCCACAAGTGTCAGTCTCTAGCTGAGCCTTTCCCTTCCTGTTTTTCTCCTTTTTAGTTGCTATGGGTTAGGGGCCAAATCTCCAGTCATACTAGAATTGCACATGGACTGGATATTTGGGAATACTGCGGGTCTATTCTATGAGCTTTAGTATGTAACATTTAATATCAGTGTAAAGAAGCCCTTTTTTAAGTTATTTCTTTGAATTTCTAAATGTATGCCCTGAATATAAGTAACAAGTTACCATGTCTTGTAAAATGATCATATCAACAAACATTTAATGTGCACCTACTGTGCTAGTTGAATGTCTTTATCCTGATAGGAGATAACAGGATTCCACATCTTTGACTTAAGAGGACAAACCAAATATGTCTAAATCATTTGGGGTTTTGATGGATATCTTTAAATTGCTGAACCTAATCATTGGTTTCATATGTCATTGTTTAGATATCTCCGGAGCATTTGGATAATGTGACAGTTGGAATGCAGTGATGTCGACTCTTTGCCCACCGCCATCTCCAGCTGTTGCCAAGACAGAGATTGCTTTAAGTGGCAAATCACCTTTATTAGCAGCTACTTTTGCTTACTGGGACAATATTCTTGGTCCTAGAGTAAGGCACATTTGGGCTCCAAAGACAGAACAGGTACTTCTCAGTGATGGAGAAATAACTTTTCTTGCCAACCACACTCTAAATGGAGAAATCCTTCGAAATGCAGAGAGTGGTGCTATAGATGTAAAGTTTTTTGTCTTGTCTGAAAAGGGAGTGATTATTGTTTCATTAATCTTTGATGGAAACTGGAATGGGGATCGCAGCACATATGGACTATCAATTATACTTCCACAGACAGAACTTAGTTTCTACCTCCCACTTCATAGAGTGTGTGTTGATAGATTAACACATATAATCCGGAAAGGAAGAATATGGATGCATAAGGTAAGTGATTTTTCAGCTTATTAATCATGTTAACCTATCTGTTGAAAGCTTATTTTCTGGTACATATAAATCTTATTTTTTTAATTATATGCAGTGAACATCAAACAATAAATGTTATTTATTTTGCATTTACCCTATTAGATACAAATACATCTGGTCTGATACCTGTCATCTTCATATTAACTGTGGAAGGTACGAAATGGTAGCTCCACATTATAGATGAAAAGCTAAAGCTTAGACAAATAAAGAAACTTTTAGACCCTGGATTCTTCTTGGGAGCCTTTGACTCTAATACCTTTTGTTTCCCTTTCATTGCACAATTCTGTCTTTTGCTTACTACTATGTGTAAGTATAACAGTTCAAAGTAATAGTTTCATAAGCTGTTGGTCATGTAGCCTTTGGTCTCTTTAACCTCTTTGCCAAGTTCCCAGGTTCATAAAATGAGGAGGTTGAATGGAATGGTTCCCAAGAGAATTCCTTTTAATCTTACAGAAATTATTGTTTTCCTAAATCCTGTAGTTGAATATATAATGCTATTTACATTTCAGTATAGTTTTGATGTATCTAAAGAACACATTGAATTCTCCTTCCTGTGTTCCAGTTTGATACTAACCTGAAAGTCCATTAAGCATTACCAGTTTTAAAAGGCTTTTGCCCAATAGTAAGGAAAAATAATATCTTTTAAAAGAATAATTTTTTACTATGTTTGCAGGCTTACTTCCTTTTTTCTCACATTATGAAACTCTTAAAATCAGGAGAATCTTTTAAACAACATCATAATGTTTAATTTGAAAAGTGCAAGTCATTCTTTTCCTTTTTGAAACTATGCAGATGTTACATTGACTGTTTTCTGTGAAGTTATCTTTTTTTCACTGCAGAATAAAGGTTGTTTTGATTTTATTTTGTATTGTTTATGAGAACATGCATTTGTTGGGTTAATTTCCTACCCCTGCCCCCATTTTTTCCCTAAAGTAGAAAGTATTTTTCTTGTGAACTAAATTACTACACAAGAACATGTCTATTGAAAAATAAGCAAGTATCAAAATGTTGTGGGTTGTTTTTTTAAATAAATTTTCTCTTGCTCAGGAAAGACAAGAAAATGTCCAGAAGATTATCTTAGAAGGCACAGAGAGAATGGAAGATCAGGTATATGCAAATTGCATACTGTCAAATGTTTTTCTCACAGCATGTATCTGTATAAGGTTGATGGCTACATTTGTCAAGGCCTTGGAGACATACGAATAAGCCTTTAATGGAGCTTTTATGGAGGTGTACAGAATAAACTGGAGGAAGATTTCCATATCTTAAACCCAAAGAGTTAAATCAGTAAACAAAGGAAAATAGTAATTGCATCTACAAATTAATATTTGCTCCCTTTTTTTTTCTGTTTGCCCAGAATAAATTTTGGATAACTTGTTCATAGTAAAAATAAAAAAAATTGTCTCTGATATGTTTTAAGGTACTACTTCTCGAACCTTTCCCTAGAAGTAGCTGTAACAGAAGGAGAGCATATGTACCCCTAAGGTATCTGTCTGGGGTGTAGGCCCAGGTCCACACAATATTTCTTTTAAGTCTTATGTTGTATCGTTAAGACTCATGCAATTTACATTTTATTCCATAACTATTTTAGTATTAAAATTTGTCAGTGATATTTCTTACCCTCTCCTCTAGGAAAATGTGCCATGTTTATCCCTTGGCTTTGAATGCCCCTCAGGAACAGACACTAAGAGTTTGAGAAGCATGGTTACAAGGGTGTGGCTTCCCCTGCGGAAACTAAGTACAGACTATTTCACTGTAAAGCAGAGAAGTTCTTTTGAAGGAGAATCTCCAGTGAAGAAAGAGTTCTTCACTTTTACTTCCATTTCCTCTTGTGGGTGACCCTCAATGCTCCTTGTAAAACTCCAATATTTTAAACATGGCTGTTTTGCCTTTCTTTGCTTCTTTTTAGCATGAATGAGACAGATGATACTTTAAAAAAGTAATTAAAAAAAAAAACTTGTGAAAATACATGGCCATAATACAGAACCCAATACAATGATCTCCTTTACCAAATTGTTATGTTTGTACTTTTGTAGATAGCTTTCCAATTCAGAGACAGTTATTCTGTGTAAAGGTCTGACTTAACAAGAAAAGATTTCCCTTTACCCAAAGAATCCCAGTCCTTATTTGCTGGTCAATAAGCAGGGTCCCCAGGAATGGGGTAACTTTCAGCACCCTCTAACCCACTAGTTATTAGTAGACTAATTAAGTAGACTTATCGCAAGTTGAGGAAACTTAGAACCAACTAAAATTCTGCTTTTACTGGGATTTTGTTTTTTCAAACCAGAAACCTTTACTTAAGTTGACTACTATTAATGAATTTTGGTCTCTCTTTTAAGTGCTCTTCTTAAAAATGTTATCTTACTGCTGAGAAGTTCAAGTTTGGGAAGTACAAGGAGGAATAGAAACTTAAGAGATTTTCTTTTAGAGCCTCTTCTGTATTTAGCCCTGTAGGATTTTTTTTTTTTTTTTTTTTTTTTGGTGTTGTTGAGCTTCAGTGAGGCTATTCATTCACTTATACTGATAATGTCTGAGATACTGTGAATGAAATACTATGTATGCTTAAACCTAAGAGGAAATATTTTCCCAAAATTATTCTTCCCGAAAAGGAGGAGTTGCCTTTTGATTGAGTTCTTGCAAATCTCACAACGACTTTATTTTGAACAATACTGTTTGGGGATGATGCATTAGTTTGAAACAACTTCAGTTGTAGCTGTCATCTGATAAAATTGCTTCACAGGGAAGGAAATTTAACACGGATCTAGTTATTATTCTTGTTAGATTGAATGTGTGAATTGTAATTGTAAACAGGCATGATAATTATTACTTTAAAAACTAAAAACAGTGAATAGTTAGTTGTGGAGGTTACTAAAGGATGGGTTTTTTTTAAATAAAACTTTCAGCATTATGCAAATGGGCATATGGCTTAGGATAAAACTTCCAGAAGTAGCATCACATTTAAATTCTCAAGCAACTTAATAATATGGGGCTCTGAAAAACTGGTTAAGGTTACTCCAAAAATGGCCCTGGGTCTGACAAAGATTCTAACTTAAAGATGCTTATGAAGACTTTGAGTAAAATCATTTCATAAAATAAGTGAGGAAAAACAACTAGTATTAAATTCATCTTAAATAATGTATGATTTAAAAAATATGTTTAGCTAAAAATGCATAGTCATTTGACAATTTCATTTATATCTCAAAAAATTTACTTAACCAAGTTGGTCACAAAACTGATGAGACTGGTGGTGGTAGTGAATAAATGAGGGACCATCCATATTTGAGACACTTTACATTTGTGATGTGTTATACTGAATTTTCAGTTTGATTCTATAGACTACAAATTTCAAAATTACAATTTCAAGATGTAATAAGTAGTAATATCTTGAAATAGCTCTAAAGGGAATTTTTCTGTTTTATTGATTCTTAAAATATATGTGCTGATTTTGATTTGCATTTGGGTAGATTATACTTTTATGAGTATGGAGGTTAGGTATTGATTCAAGTTTTCCTTACCTATTTGGTAAGGATTTCAAAGTCTTTTTGTGCTTGGTTTTCCTCATTTTTAAATATGAAATATATTGATGACCTTTAATTAACAAATTTTTTTTATCTCAAATTTTAAAGGAGATCTTTTCTAAAAGAGGCATGATGACTTAATCATTGCATGTAACAGTAAACGATAAACCAATGATTCCATACTCTCTAAAGAATAAAAGTGAGCTTTAGGGCCGGGCATGGTCAGAAATTTGACACCAACCTGGCCAACATGGCGAAACCCCGTCTCTACTAAAAATACAAAAATCAGCCGGGCATGGTGGCGGCACCTATAGTCCCAGCTACTTGGGAGGATGAGACAGGAGAGTCACTTGAACCTGGGAGGAGAGGTTGCAGTGAGCTGAGATCACGCCATTGCACTCCAGCCTGAGCAATGAAAGCAAAACTCCATCTCAAAAAAAAAAAAAGAAAAGAAAGAATAAAAGTGAGCTTTGGATTGCATATAAATCCTTTAGACATGTAGTAGACTTGTTTGATACTGTGTTTGAACAAATTACGAAGTATTTTCATCAAAGAATGTTATTGTTTGATGTTATTTTTATTTTTTATTGCCCAGCTTCTCTCATATTACGTGATTTTCTTCACTTCATGTCACTTTATTGTGCAGGGTCAGAGTATTATTCCAATGCTTACTGGAGAAGTGATTCCTGTAATGGAACTGCTTTCATCTATGAAATCACACAGTGTTCCTGAAGAAATAGATGTAAGTTTAAATGAGAGCAATTATACGCTTTATGAGTTTTTTGGGGTTATAGTATTATTATGTATATTATTAATATTCTAATTTTAATAGTAAGGACTTTGTCATACATACTATTCACATACAGTATTAGCCACTTTAGCAAATAAGCACACACAAAATCCTGGATTTTATGGCAAAACAGAGGCATTTTTGATCAGTGATGACAAAATTAAATTCATTTTGTTTATTTCATTACTTTTATAATTCCTAAAAGTGGGAGGATCCCAGCTCTTATAGGAGCAATTAATATTTAATGTAGTGTCTTTTGAAACAAAACTGTGTGCCAAAGTAGTAACCATTAATGGAAGTTTACTTGTAGTCACAAATTTAGTTTCCTTAATCATTTGTTGAGGACGTTTTGAATCACACACTATGAGTGTTAAGAGATACCTTTAGGAAACTATTCTTGTTGTTTTCTGATTTTGTCATTTAGGTTAGTCTCCTGATTCTGACAGCTCAGAAGAGGAAGTTGTTCTTGTAAAAATTGTTTAACCTGCTTGACCAGCTTTCACATTTGTTCTTCTGAAGTTTATGGTAGTACACAGAGATTGTTTTTTGGGGAGTCTTGATTCTCGGAAATGAAGGCAGTGTGTTATATTGAATCCAGACTTCCGAAAACTTGTATATTAAAAGTGTTATTTCAACACTATGTTACAGCCAGACTAATTTTTTTATTTTTTGATGCATTTTAGATAGCTGATACAGTACTCAATGATGATGATATTGGTGACAGCTGTCATGAAGGCTTTCTTCTCAAGTAAGAATTTTTCTTTTCATAAAAGCTGGATGAAGCAGATACCATCTTATGCTCACCTATGACAAGATTTGGAAGAAAGAAAATAACAGACTGTCTACTTAGATTGTTCTAGGGACAACATTACGTATTTGAACTGTTGCTTAAATTTGTGTTATTTTTCACTCATTATATTTCTATATATATTTGGTGTTATTCCATTTGCTATTTAAAGAAACCGAGTTTCCATCCCAGACAAGAAATCATGGCCCCTTGCTTGATTCTGGTTTCTTGTTTTACTTCTCATTAAAGCTAACAGAATCCTTTCATATTAAGTTGTACTGTAGATGAACTTAAGTTATTTAGGCGTAGAACAAAATTATTCATATTTATACTGATCTTTTTCCATCCAGCAGTGGAGTTTAGTACTTAAGAGTTTGTGCCCTTAAACCAGACTCCCTGGATTAATGCTGTGTACCCGTGGGCAAGGTGCCTGAATTCTCTATACACCTATTTCCTCATCTGTAAAATGGCAATAATAGTAATAGTACCTAATGTGTGGGGTTGTTATAAGCATTGAGTAAGATAAATAATATAAAGCACTTAGAACAGTGCCTGGAACATAAAAACACTTAATAATAGCTCATAGCTAACATTTCCTATTTACATTTCTTCTAGAAATAGCCAGTATTTGTTGAGTGCCTACATGTTAGTTCCTTTACTAGTTGCTTTACATGTATTATCTTATATTCTGTTTTAAAGTTTCTTCACAGTTACAGATTTTCATGAAATTTTACTTTTAATAAAAGAGAAGTAAAAGTATAAAGTATTCACTTTTATGTTCACAGTCTTTTCCTTTAGGCTCATGATGGAGTATCAGAGGCATGAGTGTGTTTAACCTAAGAGCCTTAATGGCTTGAATCAGAAGCACTTTAGTCCTGTATCTGTTCAGTGTCAGCCTTTCATACATCATTTTAAATCCCATTTGACTTTAAGTAAGTCACTTAATCTCTCTACATGTCAATTTCTTCAGCTATAAAATGATGGTATTTCAATAAATAAATACATTAATTAAATGATATTATACTGACTAATTGGGCTGTTTTAAGGCTCAATAAGAAAATTTCTGTGAAAGGTCTCTAGAAAATGTAGGTTCCTATACAAATAAAAGATAACATTGTGCTTATAGCTTCGGTGTTTATCATATAAAGCTATTCTGAGTTATTTGAAGAGCTCACCTACTTTTTTTTGTTTTTAGTTTGTTAAATTGTTTTATAGGCAATGTTTTTAATCTGTTTTCTTTAACTTACAGTGCCATCAGCTCACACTTGCAAACCTGTGGCTGTTCCGTTGTAGTAGGTAGCAGTGCAGAGAAAGTAAATAAGGTAGTTTATTTTATAATCTAGCAAATGATTTGACTCTTTAAGACTGATGATATATCATGGATTGTCATTTAAATGGTAGGTTGCAATTAAAATGATCTAGTAGTATAAGGAGGCAATGTAATCTCATCAAATTGCTAAGACACCTTGTGGCAACAGTGAGTTTGAAATAAACTGAGTAAGAATCATTTATCAGTTTATTTTGATAGCTCGGAAATACCAGTGTCAGTTGTGTATAAATGGTTTTGAGAATATATTAAAATCAGATATATAAAAAAAATTACTCTTCTATTTCCCAATGTTATCTTTAACAAATCTGAAGATAGTCATGTACTTTTGGTAGTAGTTCCAAAGAAATGTTATTTGTTTATTCATCTTGATTTCATTGTCTTTGCTTTCCTTCTAAATCTGTCCCTTCTAGGGAGCTATTGGGATTAAGTGGTCATTGATTATTATACTTTATTCAGTAATGTTTCTGACCCTTTCCTTCAGTGCTACTTGAGTTAGTTAAGGATTAATGAACAGTTACATTTCCAAGCATTAGCTAATAAACTAAAGGATTTTGCACTTTTCTTCACTGACCATTAGTTAGAAAGAGTTCAGAGATAAGTATGTGTATCTTTCAATTTCAGCAAACCTAATTTTTTAAAAAAAGTTTTACATAGGAAATATGTTGGAAATGATACTTTACAAAGATATTCATAATTTTTTTTTGTAATCAGCTACTTTGTATATTTACATGAGCCTTAATTTATATTTCTCATATAACCATTTATGAGAGCTTAGTATACCTGTGTCATTATATTGCATCTACGAACTAGTGACCTTATTCCTTCTGTTACCTCAAACAGGTGGCTTTCCATCTGTGATCTCCAAAGCCTTAGGTTGCACAGAGTGACTGCCGAGCTGCTTTATGAAGGGAGAAAGGCTCCATAGTTGGAGTGTTTTTTTTTTTTTTTTTTAAACATTTTTCCCATCCTCCATCCTCTTGAGGGAGAATAGCTTACCTTTTATCTTGTTTTAATTTGAGAAAGAAGTTGCCACCACTCTAGGTTGAAAACCACTCCTTTAACATAATAACTGTGGATATGGTTTGAATTTCAAGATAGTTACATGCCTTTTTATTTTTCCTAATAGAGCTGTAGGTCAAATATTATTAGAATCAGATTTCTAAATCCCACCCAATGACCTGCTTATTTTAAATCAAATTCAATAATTAATTCTCTTCTTTTTGGAGGATCTGGACATTCTTTGATATTTCTTACAACGAATTTCATGTGTAGACCCACTAAACAGAAGCTATAAAAGTTGCATGGTCAAATAAGTCTGAGAAAGTCTGCAGATGATATAATTCACCTGAAGAGTCACAGTATGTAGCCAAATGTTAAAGGTTTTGAGATGCCATACAGTAAATTTACCAAGCATTTTCTAAATTTATTTGACCACAGAATCCCTATTTTAAGCAACAACTGTTACATCCCATGGATTCCAGGTGACTAAAGAATACTTATTTCTTAGGATATGTTTTATTGATAATAACAATTAAAATTTCAGATATCTTTCATAAGCAAATCAGTGGTCTTTTTACTTCATGTTTTAATGCTAAAATATTTTCTTTTATAGATAGTCAGAACATTATGCCTTTTTCTGACTCCAGCAGAGAGAAAATGCTCCAGGTTATGTGAAGCAGAATCATCATTTAAATATGAGTCAGGGCTCTTTGTACAAGGCCTGCTAAAGGTATAGTTTCTAGTTATCACAAGTGAAACCACTTTTCTAAAATCATTTTTGAGACTCTTTATAGACAAATCTTAAATATTAGCATTTAATGTATCTCATATTGACATGCCCAGAGACTGACTTCCTTTACACAGTTCTGCACATAGACTATATGTCTTATGGATTTATAGTTAGTATCATCAGTGAAACACCATAGAATACCCTTTGTGTTCCAGGTGGGTCCCTGTTCCTACATGTCTAGCCTCAGGACTTTTTTTTTTTTAACACATGCTTAAATCAGGTTGCACATCAAAAATAAGATCATTTCTTTTTAACTAAATAGATTTGAATTTTATTGAAAAAAAATTTTAAACATCTTTAAGAAGCTTATAGGATTTAAGCAATTCCTATGTATGTGTACTAAAATATATATATTTCTATATATAATATATATTAGAAAAAAATTGTATTTTTCTTTTATTTGAGTCTACTGTCAAGGAGCAAAACAGAGAAATGTAAATTAGCAATTATTTATAATACTTAAAGGGAAGAAAGTTGTTCACCTTGTTGAATCTATTATTGTTATTTCAATTATAGTCCCAAGACGTGAAGAAATAGCTTTCCTAATGGTTATGTGATTGTCTCATAGTGACTACTTTCTTGAGGATGTAGCCACGGCAAAATGAAATAAAAAAATTTAAAAATTGTTGCAAATACAAGTTATATTAGGCTTTTGTGCATTTTCAATAATGTGCTGCTATGAACTCAGAATGATAGTATTTAAATATAGAAACTAGTTAAAGGAAACGTAGTTTCTATTTGAGTTATACATATCTGTAAATTAGAACTTCTCCTGTTAAAGGCATAATAAAGTGCTTAATACTTTTGTTTCCTCAGCACCCTCTCATTTAATTATATAATTTTAGTTCTGAAAGGGACCTATACCAGATGCCTAGAGGAAATTTCAAAACTGTGATCTAATGAAAAAATATTTAATAGTTCTCCATGCAAATACAAATCATATAGTTTTCCAGAAAATACCTTTGACATTATACAAAGATGATTATCACAGCATTATAATAGTAAAAAAATGGAAATAGCCTCTTTCTTCTGTTCTGTTCATAGCACAGTGCCTCATACGCAGTAGGTTATTATTACATGGTAACTGGCTACCCCAACTGATTAGGAAAGAAGTAAATTTGTTTTATAAAAATACATACTCATTGAGATGCATAGAATAATTAAGAAATTAAAAGACACTTGTAATTTTGAATCCAGTGAATACCCACTGTTAATATTTGGTATATCTCTTTCTAGTCTTTTTTTCCCTTTTGCATGTATTTTCTTTAAGACTCCCACCCCCACTGGATCATCTCTGCATGTTCTAATCTGCTTTTTTCACAGCAGATTCTAAGCCTCTTTGAATATCAACACAAACTTCAACAACTTCATCTATAGATGCCAAATAATAAATTCATTTTTATTTACTTAACCACTTCCTTTGGATGCTTAGGTCATTCTGATGTTTTGCTATTGAAACCAATGCTATACTGAACACTTCTGTCACTAAAACTTTGCACACACTCATGAATAGCTTCTTAGGATAAATTTTTAGAGATGGATTTGCTAAATCAGAGACCATTTTTTAAAATTAAAAAACAATTATTCATATCGTTTGGCATGTAAGACAGTAAATTTTCCTTTTATTTTGACAGGATTCAACTGGAAGCTTTGTGCTGCCTTTCCGGCAAGTCATGTATGCTCCATATCCCACCACACACATAGATGTGGATGTCAATACTGTGAAGCAGATGCCACCCTGTCATGAACATATTTATAATCAGCGTAGATACATGAGATCCGAGCTGACAGCCTTCTGGAGAGCCACTTCAGAAGAAGACATGGCTCAGGATACGATCATCTACACTGACGAAAGCTTTACTCCTGATTTGTACGTAATGCTCTGCCTGCTGGTACTGTAGTCAAGCAATATGAAATTGTGTCTTTTACGAATAAAAACAAAACAGAAGTTGCATTTAAAAAGAAAGAAATATTACCAGCAGAATTATGCTTGAAGAAACATTTAATCAAGCATTTTTTTCTTAAATGTTCTTCTTTTTCCATACAATTGTGTTTACCCTAAAATAGGTAAGATTAACCCTTAAAGTAAATATTTAACTATTTGTTTAATAAATATATATTGAGCTCCTAGGCACTGTTCTAGGTACCGGGCTTAATAGTGGCCAACCAGACAGCCCCAGCCCCAGCCCCTACATTGTGTATAGTCTATTATGTAACAGTTATTGAATGGACTTATTAACAAAACCAAAGAAGTAATTCTAAGTCTTTTTTTTCTTGACATATGAATATAAAATACAGCAAAACTGTTAAAATATATTAATGGAACATTTTTTTACTTTGCATTTTATATTGTTATTCACTTCTTATTTTTTTTTTAAAAAAAAAGCCTGATCAGTAAATTCAAAAGGAAAAGTAATGATAATTAATTGTTGAGCATGGACCCAACTTGAAAAAAAAAATGATGATGATAAATCTATAATCCTAAAACCCTAAGTAAACACTTAAAAGATGTTCTGAAATCAGGAAAAGAATTATAGTATACTTTTGTGTTTCTCTTTTATCAGTTGAAAAAAGGCACAGTAGCTCATGCCTGTAAGAACAGAGCTTTGGGAGTGCAAGGCAGGCGGATCACTTGAGGCCAGGAGTTCCAGACCAGCCTGGGCAACATAGTGAAACCCCATCTCTACAAAAAATAAAAAAGAATTATTGGAATGTGTTTCTGTGTGCCTGTAATCCTAGCTATTCCGAAAGCTGAGGCAGGAGGATCTTTTGAGCCCAGGAGTTTGAGGTTACAGGGAGTTATGATGTGCCAGTGTACTCCAGCCTGGGGAACACCGAGACTCTGTCTTATTTAAAAAAAAAAAAAAAAAATGCTTGCAATAATGCCTGGCACATAGAAGGTAACAGTAAGTGTTAACTGTAATAACCCAGGTCTAAGTGTGTAAGGCAATAGAAAAATTGGGGCAAATAAGCCTGACCTATGTATCTACAGAATCAGTTTGAGCTTAGGTAACAGACCTGTGGAGCACCAGTAATTACACAGTAAGTGTTAACCAAAAGCATAGAATAGGAATATCTTGTTCAAGGGACCCCCAGCCTTATACATCTCAAGGTGCAGAAAGATGACTTAATATAGGACCCATTTTTTCCTAGTTCTCCAGAGTTTTTATTGGTTCTTGAGAAAGTAGTAGGGGAATGTTTTAGAAAATGAATTGGTCCAACTGAAATTACATGTCAGTAAGTTTTTATATATTGGTAAATTTTAGTAGACATGTAGAAGTTTTCTAATTAATCTGTGCCTTGAAACATTTTCTTTTTTCCTAAAGTGCTTAGTATTTTTTCCGTTTTTTGATTGGTTACTTGGGAGCTTTTTTGAGGAAATTTAGTGAACTGCAGAATGGGTTTGCAACCATTTGGTATTTTTGTTTTGTTTTTTAGAGGATGTATGTGTATTTTAACATTTCTTAATCATTTTTAGCCAGCTATGTTTGTTTTGCTGATTTGACAAACTACAGTTAGACAGCTATTCTCATTTTGCTGATCATGACAAAATAATATCCTGAATTTTTAAATTTTGCATCCAGCTCTAAATTTTCTAAACATAAAATTGTCCAAAAAATAGTATTTTCAGCCACTAGATTGTGTGTTAAGTCTATTGTCACAGAGTCATTTTACTTTTAAGTATATGTTTTTACATGTTAATTATGTTTGTTATTTTTAATTTTAACTTTTTAAAATAATTCCAGTCACTGCCAATACATGAAAAATTGGTCACTGGAATTTTTTTTTTGACTTTTATTTTAGGTTCATGTGTACATGTGCAGGTGTGTTATACAGGTAAATTGCGTGTCATGAGGGTTTGGTGTACAGGTGATTTCATTACCCAGGTAATAAGCATAGTACCCAATAGGTAGTTTTTTGATCCTCACCCTTCTCCCACCCTCAAGTAGGCCCTGGTGTTGCTGTTTCCTTCTTTGTGTCCATGTATACTCAGTGTTTAGCTCCCACTTAGAAGTGAGAACATGCGGTAGTTGGTTTTCTGTTCCTGGATTAGTTCACTTAGGATAATGACCTCTAGCTCCATCTGGTTTTTATGGCTGCATAGTATTCCATGGTGTATATGTATCACATTTTCTTTATCCAGTCTACCATTGATAGGCATTTAGGTTGATTCCCTGTCTTTGTTATCATGAATAGTGCTGTGATGAACATACACATGCATGTGTCTTTATGGTAGAAAAATTTGTATTCCTTTAGGTACATATAGAATAATGGGGTTGCTAGGGTGAATGGTAGTTCTATTTTCAGTTATTTGAGAAATCTTCAAACTGCTTTTCATAATAGCTAAACTAATTTACAGTCCCGCCAGCAGTGTATAAGTGTTCCCTTTTCTCCACAACCTTGCCAACATCTGTGATTTTTTGACTTTTTAATAATAGCCATTCCTAGAGAATTGATTTGCAATTCTCTATTAGTGATATTAAGCATTTTTTCATATGCTTTTTAGCTGTCTGTATATATTCTTCTGAAAAATTTTCATGTCCTTTGCCCAGTTTGTAGTGGGGTGGGTTGTTTTTTGCTTGTTAATTAGTTTTAAGTTCCTTCCAGATTCTGCATATCCCTTTGTTGGATACATGGTTTGCAGATATTTTTCTCCCATTGTGTAGGTTGTCTTTTACTCTGTTGATAGTTTCTTTTGCCATGCAGGAGCTCGTTAGGTCCCATTTGTGTTTGTTTTTGTTGCAGTTGCTTTTGGCGTCTTCATCATAAAATCTGTGCCAGGGCCTATGTCCAGAATGGTATTTCCTAGGTTGTCTTCCAGGGTTTTTACAATTTTAGATTTTACGTTTATGTCTTTAATCCATCTTGAGTTGATTTTTGTATATGGCACAAGGAAGGGGTCCAGTTTCACTCCAATTCCTATGGCTAGCAATTATCCCAGCACCATTTATTGAATACGGAGTCCTTTCCCCATTGCTTGTTTTTTGTCAACTTTGTTGAAGATCAGATGGTTGTAAGTGTGTGGCTTTATTTCTTGGCTCTCTATTCTCCATTGGTCTATGTGTCTGTTTTTATAACAGTACCCTGCTGTTCAGGTTCCTATAGCCTTTTAGTATAAAATCGGCTAATGTGATGCCTCCAGCTTTGTTCTTTTTGCTTAGGATTGCTTTGGCTATTTGGGCTCCTTTTTGGGTCCATATTAATTTTAAAACAGTTTTTTCTGGTTTTGTGAAGGATGTCATTGGTAGTTTATAGGAATAGCATTGAATCTGTAGATTGCTTTGGGCAGTATGGCCATTTTAACAATATTAATTCTTCCTATCTATGAATATGGAATGTTTTTCCATGTGTTTGTGTCATCTCTTTATACCTGATGTATAAAGAAAAGCTGGTATTATTCCTACTCAATCTGTTCCAAAAAATTGAGGAGGAGGAACTCTTCCCTAATGAGGCCAGCATCATTCTGATACCAAAACCTGGCAGAGACACAACAGAAAAAAGAAAACTTCAGGCCAATATCCTTGATGAATATAGATGCAAAAATCCTCAACAAAATACTAGCAAACCAAATCCAGCAGCACATCAAAAAGCTGATCTACTTTGATCAAGTAGGCTTTATCCCTGGGATGCAAGGTTGGTTCAACATACACAAATCAATAAGTGTGATTCATCACATAAACAGAGCTAAAAACAAAAACCACAAGATTATCTCAATAGGTAGAGAAAAGGTTGTCAATAAAATTTAACATCCTCCATGTTAAAAACCTTCAGTAGGTCAGGTGTAGTGACTCACACCTGTAATCCCAGCACTTTGGGAGGCCAAGGCGGGCATATCTCTTAAGCCCAGGAGTTCAAGACGAGCCTAGGCAGCATGGTGAAACCCCATCTCTACAAAAAAAAAAAAAAAAAAAAATTAGCTTGGTATGGTGACATGCACCTATAGTCCCAGCTATTCAGGAGGTTGAGGTGGGAGGATTGTTTGAGCCCGGGAGGCAGAGGTTGGCAGCGAGCTGAGATCATGCCACCGCACTCCAGCCTGGGCAACGGAGTGAGACCCTGTCTCAAAAAAGAAAAATCACAAACAATCCTAAACAAACTAGGCATTGAAGGAACATGCCTCAAAAAAATAAGAACCATCTATGACAGACCCATAGCCAATATCTTACCAAATGGGCAAAAGCTGGAAGTATTCTCCTTGAGAACCGTAACAAGACAAGGATGTCCACTCTCACCACTCCTTTTCAGCATAGTTCTGGAAGTCCTAGCCAGAGCAATCAGGAAAGAGAAAGAAAGAAAGACATTCAGATAGGAAGAGAAGAAGTCAAACTATTTCTGTTTGCAGGCAGTATAATTCTGTACCTAGAAAATCTCATAGTCTCTGCCCAGAAACTCCTAAATCTGTTAAAAATTTCAGCAAAGTTTTGGCATTCTCTATACTCCAACACCTTCCAAAGTGAGAGCAAAATCAAGAACACAGTCCCATTCACAATAGCCGCAAAACGAATAAAATACCTAGGAATCCAGCTAACCAGGGAGGTGAAAGATCTCTATGAGAATTACAAAACACTGCTGAAAGAAATCAGAGATGACACAAACAAATGGAAATGTTCTTTTTTAACACCTTGCTTTATCTAATTCACTTATGATGAAGATACTCATTCAGTGGAACAGGTATAATAAGTCCACTCGATTAAATATAAGCCTTATTCTCTTTCCAGAGCCCAAGAAGGGGCACTATCAGTGCCCAGTCAATAATGACGAAATGCTAATATTTTTCCCCTTTACGGTTTCTTTCTTCTGTAGTGTGGTACACTCGTTTCTTAAGATAAGGAAACTTGAACTACCTTCCTGTTTGCTTCTACACATACCCATTCTCTTTTTTTGCCACTCTGGTCAGGTATAGGATGATCCCTACCACTTTCAGTTAAAAACTCCTCCTCTTACTAAATGTTCTCTTACCCTCTGGCCTGAGTAGAACCTAGGGAAAATGGAAGAGAAAAAGATGAAAGGGAGGTGGGGCCTGGGAAGGGAATAAGTAGTCCTGTTTGTTTGTGTGTTTGCTTTAGCACCTGCTATATCCTAGGTGCTGTGTTAGGCACACATTATTTTAAGTGGCCATTATATTACTACTACTCACTCTGGTCGTTGCCAAGGTAGGTAGTACTTTCTTGGATAGTTGGTTCATGTTACTTACAGATGGTGGGCTTGTTGAGGCAAACCCAGTGGATAATCATCGGAGTGTGTTCTCTAATCTCACTCAAATTTTTCTTCACATTTTTTGGTTTGTTTTGGTTTTTGATGGTAGTGGCTTATTTTTGTTGCTGGTTTGTTTTTTGTTTTTTTTTGAGATGGCAAGAATTGGTAGTTTTATTTATTAATTGCCTAAAGGTCTCTACTTTTTTTAAAAGATGAGAGTAGTAAAATAGATTGATAGATACATACATACCCTTACTGGGGACTGCTTATATTCTTTAGAGAAAAAATTACATATTAGCCTGACAAACACCAGTAAAATGTAAATATATCCTTGAGTAAATAAATGAATGTATATTTTGTGTCTCCAAATATATATATCTATATTCTTACAAATGTGTTTATATGTAATATCAATTTATAAGAACTTAAAATGTTGGCTCAAGTGAGGGATTGTGGAAGGTAGCATTATATGGCCATTTCAACATTTGAACTTTTTTCTTTTCTTCATTTTCTTCTTTTCTTCAGGAATATTTTTCAAGATGTCTTACACAGAGACACTCTAGTGAAAGCCTTCCTGGATCAGGTAAATGTTGAACTTGAGATTGTCAGAGTGAATGATATGACATGTTTTCTTTTTTAATATATCCTACAATGCCTGTTCTATATATTTATATTCCCCTGGATCATGCCCCAGAGTTCTGCTCAGCAATTGCAGTTAAGTTAGTTACACTACAGTTCTCAGAAGAGTCTGTGAGGGCATGTCAAGTGCATCATTACATTGGTTGCCTCTTGTCCTAGATTTATGCTTCGGGAATTCAGACCTTTGTTTACAATATAATAAATATTATTGCTATCTTTTAAAGATATAATAATAAGATATAAAGTTGACCACAACTACTGTTTTTTGAAACATAGAATTCCTGGTTTACATGTATCAAAGTGAAATCTGACTTAGCTTTTACAGATATAATATATACATATATATATCCTGCAATGCTTGTACTATATATGTAGTACAAGTATATATATATGTTTGTGTGTGTATATATATATAGTACGAGCATATATACATATTACCAGCATTGTAGGATATATATATGTTTATATATTAAAAAAAAGTTATAAACTTAAAACCCTATTATGTTATGTAGAGTATATGTTATATATGATATGTAAAATATATAACATATACTCTATGATAGAGTGTAATATATTTTTTATATATATTTTAACATTTATAAAATGATAGAATTAAGAATTGAGTCCTAATCTGTTTTATTAGGTGCTTTTTGTAGTGTCTGGTCTTTCTAAAGTGTCTAAATGATTTTTCCTTTTGACTTATTAATGGGGAAGAGCCTGTATATTAACAATTAAGAGTGCAGCATTCCATACGTCAAACAACAAACATTTTAATTCAAGCATTAACCTATAACAAGTAAGTTTTTTTTTTTTTTTTGAGAAAGGGAGGTTGTTTATTTGCCTGAAATGACTCAAAAATATTTTTGAAACATAGTGTACTTATTTAAATAACATCTTTATTGTTTCATTCTTTTAAAAAATATCTACTTAATTACACAGTTGAAGGAAATCGTAGATTATATGGAACTTATTTCTTAATATATTACAGTTTGTTATAATAACATTCTGGGGATCAGGCCAGGAAACTGTGTCATAGATAAAGCTTTGAAATAATGAGATCCTTATGTTTACTAGAAATTTTGGATTGAGATCTATGAGGTCTGTGACATATTGCGAAGTTCAAGGAAAATTCGTAGGCCTGGAATTTCATGCTTCTCAAGCTGACATAAAATCCCTCCCACTCTCCACCTCATCATATGCACACATTCTACTCCTACCCACCCACTCCACCCCCTGCAAAAGTACAGGTATATGAATGTCTCAAAACCATAGGCTCATCTTCTAGGAGCTTCAATGTTATTTGAAGATTTGGGCAGAAAAAATTAAGTAATACGAAATAACTTATGTATGAGTTTTAAAAGTGAAGTAAACATGGATGTATTCTGAAGTAGAATGCAAAATTTGAATGCATTTTTAAAGATAAATTAGAAAACTTCTAAAAACTGTCAGATTGTCTGGGCCTGGTGGCTTATGCCTGTAATCCCAGCACTTTGGGAGTCCGAGGTGGGTGGATCACAAGGTCAGGAGATCGAGACCATCCTGCCAACATGGTGAAACCCCGTCTCTACTAAGTATACAAAAATTAGCTGGGCGTGGCAGCGTGTGCCTGTAATCCCAGCTACCTGGGAGGCTGAGGCAGGAGAATCGCTTGAACCCCGGAGGTGTAGGTTGCAGTGAGTCAAGATCGCGCCACTGCACTTTAGCCTGGTGACAGAGCTAGACTCCGTCTCAAAAAAAAAAAAAAATATCAGATTGTTCCTACACCTAGTGCTTCTATACCACACTCCTGTTAGGGGGCATCAGTGGAAATGGTTAAGGAGATGTTTAGTGTGTATTGTCTGCCAAGCACTGTCAACACTGTCATAGAAACTTCTGTACGAGTAGAATGTGAGCAAATTATGTGTTGAAATGGTTCCTCTCCCTGCAGGTCTTTCAGCTGAAACCTGGCTTATCTCTCAGAAGTACTTTCCTTGCACAGTTTCTACTTGTCCTTCACAGAAAAGCCTTGACACTAATAAAATATATAGAAGACGATACGTGAGTAAAACTCCTACACGGAAGAAAAACCTTTGTACATTGTTTTTTTGTTTTGTTTCCTTTGTACATTTTCTATATCATAATTTTTGCGCTTCTTTTTTTTTTTTTTTTTTTTTTTTTTTTTCCATTATTTTTAGGCAGAAGGGAAAAAAGCCCTTTAAATCTCTTCGGAACCTGAAGATAGACCTTGATTTAACAGCAGAGGGCGATCTTAACATAATAATGGCTCTGGCTGAGAAAATTAAACCAGGCCTACACTCTTTTATCTTTGGAAGACCTTTCTACACTAGTGTGCAAGAACGAGATGTTCTAATGACTTTTTAAATGTGTAACTTAATAAGCCTATTCCATCACAATCATGATCGCTGGTAAAGTAGCTCAGTGGTGTGGGGAAACGTTCCCCTGGATCATACTCCAGAATTCTGCTCTCAGCAATTGCAGTTAAGTAAGTTACACTACAGTTCTCACAAGAGCCTGTGAGGGGATGTCAGGTGCATCATTACATTGGGTGTCTCTTTTCCTAGATTTATGCTTTTGGGATACAGACCTATGTTTACAATATAATAAATATTATTGCTATCTTTTAAAGATATAATAATAGGATGTAAACTTGACCACAACTACTGTTTTTTTGAAATACATGATTCATGGTTTACATGTGTCAAGGTGAAATCTGAGTTGGCTTTTACAGATAGTTGACTTTCTATCTTTTGGCATTCTTTGGTGTGTAGAATTACTGTAATACTTCTGCAATCAACTGAAAACTAGAGCCTTTAAATGATTTCAATTCCACAGAAAGAAAGTGAGCTTGAACATAGGATGAGCTTTAGAAAGAAAATTGATCAAGCAGATGTTTAATTGGAATTGATTATTAGATCCTACTTTGTGGATTTAGTCCCTGGGATTCAGTCTGTAGAAATGTCTAATAGTTCTCTATAGTCCTTGTTCCTGGTGAACCACAGTTAGGGTGTTTTGTTTATTTTATTGTTCTTGCTATTGTTGATATTCTATGTAGTTGAGCTCTGTAAAAGGAAATTGTATTTTATGTTTTAGTAATTGTTGCCAACTTTTTAAATTAATTTTCATTATTTTTGAGCCAAATTGAAATGTGCACCTCCTGTGCCTTTTTTCTCCTTAGAAAATCTAATTACTTGGAACAAGTTCAGATTTCACTGGTCAGTCATTTTCATCTTGTTTTCTTCTTGCTAAGTCTTACCATGTACCTGCTTTGGCAATCATTGCAACTCTGAGATTATAAAATGCCTTAGAGAATATACTAACTAATAAGATCTTTTTTTCAGAAACAGAAAATAGTTCCTTGAGTACTTCCTTCTTGCATTTCTGCCTATGTTTTTGAAGTTGTTGCTGTTTGCCTGCAATAGGCTATAAGGAATAGCAGGAGAAATTTTACTGAAGTGCTGTTTTCCTAGGTGCTACTTTGGCAGAGCTAAGTTATCTTTTGTTTTCTTAATGCGTTTGGACCATTTTGCTGGCTATAAAATAACTGATTAATATAATTCTAACACAATGTTGACATTGTAGTTACACAAACACAAATAAATATTTTATTTAAAATTCTGGAAGTAATATAAAAGGGAAAATATATTTATAAGAAAGGGATAAAGGTAATAGAGCCCTTCTGCCCCCCACCCACCAAATTTACACAACAAAATGACATGTTCGAATGTGAAAGGTCATAATAGCTTTCCCATCATGAATCAGAAAGATGTGGACAGCTTGATGTTTTAGACAACCACTGAACTAGATGACTGTTGTACTGTAGCTCAGTCATTTAAAAAATATATAAATACTACCTTGTAGTGTCCCATACTGTGTTTTTTACATGGTAGATTCTTATTTAAGTGCTAACTGGTTATTTTCTTTGGCTGGTTTATTGTACTGTTATACAGAATGTAAGTTGTACAGTGAAATAAGTTATTAAAGCATGTGTAAACATTGTTATATATCTTTTCTCCTAAATGGAGAATTTTGAATAAAATATATTTGAAATTTTGCCTCTTTCAGTTGTTCATTCAGAAAAAAATACTATGATATTTGAAGACTGATCAGCTTCTGTTCAGCTGACAGTCATGCTGGATCTAAACTTTTTTTAAAATTAATTTTGTCTTTTCAAAGAAAAAATATTTAAAGAAGCTTTATAATATAATCTTATGTTAAAAAAAACTTTCTGCTTAACTCTCTGGATTTCATTTTGATTTTTCAAATTATATATTAATATTTCAAATGTAAAATACTATTTAGATAAATTGTTTTTAAACATTCTTATTATTATAATATTAATATAACCTAAACTGAAGTTATTCATCCCAGGTATCTAATACATGTATCCAAAGTAAAAATCCAAGGAATCTGAACACTTTCATCTGCAAAGCTAGGAATAGGTTTGACATTTTCACTCCAAGAAAAAGTTTTTTTTTGAAAATAGAATAGTTGGGATGAGAGGTTTCTTTAAAAGAAGACTAACTGATCACATTACTATGATTCTCAAAGAAGAAACCAAAACTTCATATAATACTATAAAGTAAATATAAAATAGTTCCTTCTATAGTATATTTCTATAATGCTACAGTTTAAACATATCACTCTTATATAATACTATTTTGATTTTGATGTAGAATTGCACAAATTGATATTTCTCCTATGATCTGCAGGGTATAGCTTAAAGTAACAAAAACAGTCAACCACCTCCATTTAACACACAGTAACACTATGGGACTAGTTTTATTACTTCCATTTTACAAATGAGGAAACTAAAGCTTAAAGATGTGTAATACACCGCCCAAGGTCACACAGCTGGTAAAGGTGGATTTCATCCCAGACAGTTACAGTCATTGCCATGGGCACAGCTCCTAACTTAGTAACTCCATGTAACTGGTACTCAGTGTAGCTGAATTGAAAGGAGAGTAAGGAAGCAGGTTTTACAGGTCTACTTGCACTATTCAGAGCCCGAGTGTGAATCCCTGCTGTGCTGCTTGGAGAAGTTACTTAACCTATGCAAGGTTCATTTTGTAAATATTGGAAATGGAGTGATAATACGTACTTCACCAGAGGATTTAATGAGACCTTATACAATCCTTAGTTCAGTACCTGACTAGTGCTTCATAAATGCTTTTTCATCCAATCTGACAATCTCCAGCTTGTAATTGGGGCATTTAGAACATTTAATATGATTATTGGCATGGTAGGTTAAAGCTGTCATCTTGCTGTTTTCTATTTGTTCTTTTTGTTTTCTCCTTACTTTTGGATTTTTTTATTCTACTATGTCTTTTCTATTGTCTTATTAACTATACTCTTTGATTTATTTTAGTGGTTGTTTTAGGGTTATACCTCTTTCTAATTTACCAGTTTATAACCAGTTTATATACTACTTGACATATAGCTTAAGAAACTTACTGTTGTTGTCTTTTTGCTGTTATGGTCTTAACGTTTTTATTTCTACAAACATTATAAACTCCACACTTTATTGTTTTTTAATTTTACTTATACAGTCAATTATCTTTTAAAGATATTTAAATATAAACATTCAAAACACCCCAATTAAAAGTCAGAGATTGTTAATACCACATGATCTCACTTACACACAGAATTGAAAAACTTGGAACTCATAGAAGCAGAGAGTAAAAACATGGTTACCAGGTGCTGGGGAGAGGCGGTGGGCTGGGGAGATGTTGGTCAAAGTTAGACAGGAGGAATAAGTTCAAGAGATCTATTGTACAACTTATTCAGTTAGATAGGAGGAATAAGCTAAAGATCAAGAGATCTATTGTACAATGTGACTATAACCAACAACATATATTGTACACTTGAAAATTGCTAACAGTATCTTTTAAGTGTTCTCTCTACAAATAAATATGTGAGGTAATGTATATATTAATTAACTGTAGTCATTTCACAATGTATACTTATTTCAAAACATCATATTGTATGCTATAAATATATACAACTTTTATTTTTCAATTTTAGAAATGTCCTTAAAAAATCAGATTTTCAGATCAGATAAAAAAGCAAGACCCAACTATATGCTGCCAACAGGAAACACACCTTAAAAATAAAGGACGAACAAACAGATTAAAAGTAAAAGGATGGAGAAAAGATACATCATATTGGTAATTAGAAGAAAACTGGAGTGACAATATGAAACAAAATAGATTTCAGAGCAAAGAATATTACCAGGGGTAAAAATGATCATTTTATAATGATAAAAGAGTCAGTTCAGCAAAAGGATATAACAGTCCTAAATGTTTTTTCACCTCATAGCTGTGTCAAAATAGATGAAGCAAAAACTGATAGAACTGTAAGAAGTAGACAAGTCCACAATTATGTTTGGAGATTTTTTTTTTTTTTTTTTTTTGTCGCCCAGGCTGGAGTGCAGTGGCAGGATCTCAGCTCACTGCAAGCTCCGCCTCCCAGGTTCACGCCATTCTCCTGCTTCAGCCTCCCCAGTAGCTGGGACTACAGGCGGCCACCACCACGCCTGGCTAATTTTTTTGTATTTTTAGTAGAGACGGGGTTTCACCGTGTTAGCCAGGATGGTCTCGATCTCCTGACCTCGTGATCTGCCTGCCTCGGCCTCCCAAAGTGCTGGGATTACAGGCATGAGCCACTGCACGCAGCCTGGAGATTTTAATATCCTTTCAATGTTTAGTAGAACAAGAATACACAAAATCAGTAAGGATATAGAAGATTAGAACAAGACTATCAAACAATTTGACTTAAATGACATTTGTAGAGCACAGCAGTCCCCAACAACAATAAATCACACATTCTTTCCAAGAGTACATGAAACATGTACCAAGATAGACCGTATTTTGAGCCATGAAACAAATCTTGATAAATTTAAAAGGATTCAAGTCATAGAAAATATGTTCTCTGACCACAATGGAATTAAATTATTAACCAATAACAAATACCTGGGAAAACCTCAAAAACTTGGACACCAGCGCTTTTAAAAGACTAAATAATTTCTAAATTATCTGTGTTGGGGGGAAAAGAGAAATGGATTAGAGAGCAAAAAGGGTATCAGAGTGCTGTGGTACGATTTTTATGAAGAGTGGAACAGAATCTGCCTTTGGCGTTTCCCCACTACAGCCCATTCTTCACATTGATAACAGCATGATCCTTCTAAAATTAAATCTAACGATCACTTCTGCTTAATGGCTCTCCAACACTTACAGAATTAGGTCCAAAATTCTAGCACAGTTTCTGTTCATCTTTCTAACCTTTCTTCCCACAGGTCTAGCTAGTACGTATTTCTTTTATTGCATTTATTACACTATTCCTTTGCTTATCTATCTCCCCACCTAGGCTAAAGAACAAGATTCTTGTCTTTTTCATTTTTGTGTCTCAGTGCCTAGCATGGTGCCAGGCACACAGCATGCTTCCAGTAAATGTTAGCTGGATGGATGTAATGAGTATATTAAATATTAATTTATTTGTTTTTCCCCAAAAAGAATTATTTCCTGCAAATCAAGGAAATTGCTTTCTTTATATAATCAAAAACTTATTTTCCCAGAAGATTCTTCATTAAAAATTAAGCCTATGCACAACCTAGCTCTAAAGTTTCAAAGATTTTAGGCAGCAATTTTTCAATCTTTTTGAAGTAATACATTTGAATCTTTTCAAATTTCTGTTTCTGCATTTGTGCCACACCATCTCATCTCTTGCTGAAATGTTTTTGTTAAATTAATTGCTTGATAAATTGCTAAGTACTTTTCATCAGACCAATTAGGACAATAGTAAGTATCCATCTGTGGAGCGCGGACATTCAAGAAATCTGATCCAGTATTTAGAAAGTCATTCCTGAGCTGAGTTGGCTCAAACTGGCACCTTCTGGCATTTGCTTGTGGGTGGGGAATGTGGAATGCTTTGAAAGCTGAATGAGTTTGTCAAGTTTTAAAATTCCCTTATGGCTAAAGGAAAACAACATTCATTGTTTAAAAACACCATTGTTTGTTTTTTCTGCTTTTTTGTTCTTTGGAGCCTGAATCTGCAAAAACACTCACACCCAGCATTTTGCTTCATGTACCACTCCTAAGATGTTTTTAGAGACTTGAATAGTGTCTCCGCACTACTTTTTATTGTGATTGTTCAGAATGTTCATAACAAATGGTAAAAAGTCAGTTTTAGTGCTCAAATTGAGTTTTATGGAGAAAGACCATAATTTATGTTTGTCATTGTAAATTGATAGGAGAATTTTTGGAAGTTTGCGTCCTAGAACCAGATTTCCAAGGCTCAGATCCTTATTTTCTCACTTCCTAGCTGTGTGACCTTAGACAAGGTATTAAACCTGTCTGTGCTGCCTCAGTGTCCTCATCTATTCTTTAAGAGTAAGAATAGAACCTACCCGATAGAGTCACTTGAAGATTAAGTGGGTTAGTAAATTCAGAATGCTTGGAACAGTAACTAGCACAGAATAAGTGTCCAATAAAATTGGGTTGCAGCTATTATCAGTATTATTCCTGTCATAATCATCATCACCATTAAGCAATTAAATGTAGAGTTCCAAAATTTGATTATGAAACTACAGTTATACAGCCATGATTCCCGGTGATACCACGTCAGTAACAAGATTATTTCCTTAGCTTGAGCCAGTCACTACCTCATTGCATGTGGCAGAGTGTGTTGCCGTAGGCAAATGTCATTGTAGGGAATGAAAAAAAAATTGCCTGTGAGCTGCTCTCCAGAGGCCTCATCCCATTTTCCCATCGTCCACTTTACTCCATCTCCACTGCCACTATTAGGACCTTATCATTTCTTGTCTAGATTAATTCAACAGCTTCCTTCCTTCTAGTCTCCATGATTTCACCCACTAGCCATCCCCTCCCCTTTGCCCAATTTTCTCCATTTATGGTAGAGTGATCTTTCTAATAGGAAACTCCTGACTTGCCTTAAAAAGCCCTCATTGAGGCCGGACGTGGTGGCTCATGCCTGTAATCCCAGCACTTTGGGAGGCCGAGGCAGGTGGATCACGAGGTCAAGAGATTGAGACCATCGTGACTAACACAGTGAAACCCCATCTGTACTAAAAATACAAGAAATTAGCCAGGCGTGGTGGCGGGTGCCTGTAGTCGCAGCTACTTGGGAGGCTGAGGCAGGAGAATGGCGTGAACCCGGGAGGCAGAGCTTGCAGTGAGCCGAGATTGCGCCACTGCACTCCAGCCTGGGCGACAGAGTGAGACTCCGTCTCAAAAAAAAAAAGCCCTCATTGACAACCTTCAACCCACAATCCATGGTGAAGCACAGGAGCCTTGGGGATCTGCCCCCAGCACACCTCTCCACCCTTGTCTCTCACTGCTCCTGCCTTCATGGAGAGCCCTGATGAACTATTTGTAGTTTCCCCTGACTCACCTTGCTGTTACTGGGCCTGTGTGCGTGTTGCTCCCACTACCTGCAATACGCTTACCCACTTCACCTGGGTGAACTTTACTTAGGATTCACCTTAGGTGGGCATCATGTTCTTCCAGGCCCCTCCTCTAACTTTTAGTTGAGAGTATTCCAGACTTAAGGCTCCATGGGATAGGGATCTTGTCTATGCACCAGCTTATTCCCAACTGCCTGGCACGTAATGCATTTATTAAATATATATTGAATTGATTACCCTACTTGGGGCTCTTGTTTGCTTCTACACTTACAGTTCTAGCATAGCACTTAACTCATTATCATGCATCATTATTATGGGTTTGTTTTGTCTCCCATTAGACTGTGAGCTCCACAAGGCTGTGTCCTTGTCTTATACATCATTGTATTTCCAGCTTCCAACATAGTGCTTGCCATGACACAGGAAGTCAGTAAGCTCTGAATGAATGAATAGTATCTACATACCATTAATCTGAGGTTTAAAGTTTCCCCAAATTCTGAAGCAAGGGGATTTACGGACTTCCCTGACAATTTTTGGATGTCATCCCAATGATACCACTAACATTTTAAGGGACAGCTTGCATATATACATTTTTCTGGATGGCAGTTTTTTTTCCCACAGGCTTCATCAGATATTTCTCCATAGCCTTCCTCAGATTCTCAAAGGGGTCTCTGATTCCCCCAAAAGATAAGAAACTGTCATAAAAAATTATTTCTAAATATCAATTGTTAAATAAAATGTTTGCAAAGCAGCCTGATGAATCATTTCAGGCCACTTGACCCCGATGAGTTAGAGAGTTTGTGCTCTGCAATCTGACTGCTTCCAGCAGTCTCACTGCTGCTGGACTGTGGCACTTCCAATTGGCAGCAGGGCAAGTTTCTTCTGGATGAATATTCTGTCATAGGGGTCCCCCTTCCACACATACCTGTAGGAGCAGTTTGAAACTCATATGCATGGTCTTCCTGGTTCTAGGCACATGAGTCATTTAAGCTGCTGGAGCCAGGACCAGCTAGTATGCTAGCCCGGCATTCAGAAAGTTAAAATTTGGGGTCAAAACTGAGAACCTTCTTTGATCCACCTTGGCCAGACATTTTCTCTGGCTTCCATTAATAGCCTCAACATTTTTTTTTTTTCTGGCCTAGACCCACACAGGCAAGAGACCAGAGCTTCTCTAAGGAGCTAAGGGAAAGCACATTTTAAAAATAACTTGAGCAAATGAATTCATCTGGCAAAAGCAACCCCACTACGTAAAATAAACCTTTTTAGTTTCGCAATAGCAGTTCCTGAAAATGTAAACAACCTCAGGGTCTACATGCACTGAATCATTTGCTGAACAGAAAGTCCCTGGTCCAAATTCTGCAAGAATAAACACCTTACAAAACTAGGGGTCAATGACCTTCATATGGGAACAAGGAGGGTGTGGGGGGCAGCAACCCACCCTGAGGACAATGAGAAAGTCTTGAGACTTGATATTCAAAATGCTGGCTTTCTAAACCAAAAACTGGCATGAGTGGAGGGAGAAGGGGAGGGTGGGCACAGTCTATGCCTCAGGCTCTTGCTCAGACCCTACCAGGCCCCTGCCTTCCCTAGGGAAAGCGAGAGTCTACTCACTGTCATGAAGCCAGAGGAAGGCCCTGCAGGTTTCACTGTGTGTTCTGTTGACAAGATGATGGTTCCATTGAAACTGTAATAACATACTTGGCCAACTAAGCCCATACGATCGTAGTAACTTTGTACCCAGTCCTAGCTTTTCAAACATAATGATAATATGTTCTTTCTAATGTGGCCCATACTGTTCTAATGAACTTATGCTGAGTTTTTCTGAGTACTAGAATAATATTCGCCATAAATAATAGATATAATTATTCTCATTTAATATTTGCGTAGCTCTTCTTTAAAGCAGAAAGTATTTTCTCATTCCTTACTAGAACCTTTCTGTGTGAGGAGCACTGAGCTAGAACCCATATCTTAGAATGGTCAGAATTTGGAGAAATTCAGGGAAAAGGCACTGGACTCATTTTTAAAGACTAGAAAATGCAACCTCCAGAAAAAGATTCAAGAGTTTTTTACTCCCAGAGATGTAGGAAAGATTGGAGTAAATCTTAATATTATATTTCAGGTAAACAAAGGATCACTGTCAAAATAGCAGCATTTATTGAGTAATGGCTGTGTGCCAGGTACTTTACAGTTTCACATTTAACCCTCATAATAACCTTGTAAAGTGGATATCCCCTCAGTACATGATGAGAACACTGAAGCTTAGGTTAAATGATTGTCCAAATCGGACAATCATTTTCAAAATCTCCCCCTTTTTTTCTCCTTTCTTATCTGCAAGGCAGATTGCCCTTTCCCTTTCAGTGAAACTTGTGCATGACCACATGACTCTCTTTGGCCAATGAAACATGAACAAGCAGCGTTTATCACTTTCAGATGGAAGGCTTTGCATGAGCTTTGCCTCCTTTTCACTCTGCCACAGTGGCCACTAACATTCCAGATAGTGGCGCTCTGCAGGCTAGGTCCTATAGTGGGAGCTATGGGCAGAGCCCCCTTTCCCACCCCCATCAAGATGTGCATGCTGCATAAGCCATGCATTAATCTTTGCAGTTTTAAGCCACTAAGTTTTGGAGTTATATTAATCATTAATCATGGTTCTCAAGAGAAACAGAGTGGGGGAGTGGTATTCATTATGGGAATTGGCTTACATGATTATGGAAGCTGAGTAGTCCCCCAGTCTGCTGTTTTTGAGCTGGAGAACTAGAGGAGCCAGTGGTATAATTCAGCCCAAGCCTGAAGGCCTGAGAAATGGGATGGGGGAATTGGGAGGGTGGGTGTGCTAGGGTAGGATAAGTCCTGAAGTTCAAAGGCCAGCCAGAAGGTGGATGTTTCAGCACCAGAAGAGAGAGCAAATTCGCTTTTCTTCTGCCTTTTTGTCCTCTCTGGGCCCTCAATGGATTGGATGATGCCCTCCCACATTGGTAAGGGTGGATCTTCTATACTCAGTCTGCTAATTTCTTCCAGAAACATCTTCACAGACACATCCAGAAATAATGTTTTACCAGCTATCTCGGTATCCCTTAGCCTAGTCCATATTTAAAAATTAATGATCACAAGCAGTTGTTTGTTTCCACAGCAAAACCTGGGTGACAGACCAAGTGACCCAGATGACTAGAATTTGACCTTCTTTTGTTGCCCACACCATACTCTGAACTAACATGCTGTGCTGCCTTCCAAGTGGAGAATGATGGCTAAGTATCTTCTACCTAATTTGAGTCACAGAAAAAAAAAAAAAAGGTTATTAACTGCAGTGACAAGAATTGTGATTCCCCAGGGGGCAGATCAAGACTGATAGATAAGAGAAGTGAGGAACATCTGGGGAATGTCCATTGAAAATTTACTCAGAAGAGAAGAATAATTAATATAATAATATGATATATTGAATTATAATAAATAATATTTTGATGTATTTCCTTCCAGGCATGTTTAAGTTATAGACTTTGAGTATATTTTCTCAAAGGGGGTTCTATGTAAGAGACTATTTCTTAATATAGTTCCTAGCTTGGAATTGCTCTTGCTGGTTTAAGCTGAGCTTATTTTATTACAGACTTCACAACAATAACGTTTTCCTTCACTAGTCAGTACACAAGATGGTCTTCATTTCCAGTTTGGAATCCCACACTATCAGAGCCTGAGACAAGGACTAGTATGCAGTTAGTTTGTTTGGGAGGTGATTCCAGGAAGTGGGAATGAGAGATCAGTCAGCCTGCAACACGAAGGAGGAAAAGTCAATATAAGGATGAATTTGGCAATTGGCCGTTTCATGCAACTGGGGCTAAATTTTGCTTGGCTCTCTAAGAAATGTAAAGAATGCCTCCCGTAATTGCTCACCTCAAGTATTTATTCATTGGCTCTCATGCTCCATTGGTTGTCCATGAGAACTTTAGCCCTCCCTCGCTGCAGCACAGACACTGTGCTTTCTCCTAGGCTGAGCAAGCTCCTGCATCTGTGGAAACCGTCCCGGGGCAGATAGTGAAATAATGACTGCTGCGTGCTTGAGATCTGGGAAAGAGGCCACATCATAAGTGCACTGAAATCAGAGATGTGTCAAGAGATGTGACACAGGGCATCTGAGGTGTCTACTGCACCAGCTATAACTCCCTAAACGCTAATCTCAGTTCTTACAGAGGGGATGGATGCAAGGGAACAGTCATGATTGAGAGCACCGAAGAAGCTCTGTATGAACCTTAGGCAAGTTTCCTAATCTCCAAAATGAAGGTAATAATACCCACCTTCCAAGATCTTCGGGAGGAATAGATGAACTAATGTATGTGAAAATGTCCAGCACAGGTCCTAACCCATAGTAGGTGCTCACCAAATGTTAGTTCCCTGCCCTCCACGTTGTGTGTATCCGGAGCTGCACTAGATGCTGAGGCAAATGGTCTCAAATGTACTTTAACACTTAATGACTGAGATTTTTTCTGAGCTGCCTACAGGTTATTGACTATATTCATTATTAATAATAATATATATGGCCACTTCAGGCAACTGGGGCTAAATTTTGCTTGGCTCTCTAAGAAATGTAAAGAATGCCTCCTGTAATTGCTCACCTCAAGTATTTATTCATTGGCTCTCGTGCTTTATTGGTTGTCCCTGAGGACTTTAGCCCTCTCTCACTGCAGCACAGACACTGTGCTTTCTCCTAGTTTCTGTGGCAAGTGACAGGAGCCCACCTCAAACTAAAGCAAAAGGGACTTCATTGGCTCTTGTAGCTAGGAATTCCAGGGTTGACACTGGCTTTGGGCACTACTGGATGCAGGAATTCAAACAATGTCTTCAACTCTTTCTTTTGGTGTTTCTCTCAGCTGTGCTTCTCTTGTCGTTTCTTTTTCCCATTTTACAGATAAGTTCATCCGTAACTGAGAGAGGTGAAAAGGGGATGGCTGCAGAGAACTCTGGCTTATATCATCCTTGCTTGCTGACCTCAAGGTCCATGTATAAATTCTCAGAGAAGAAGCCCTCTGGTTGGTGATGCTTGGAACATGCCCTGGAGGGTGGGCCCCTTGAAGTGGAGCTTGCTGGAACCACATGGGCTGGAGCAAGGCGCTAGGGCCAGAAGAGAGAGGTAGGCAGGGCTGCTGGCCAGGCACTCTTCACCAAGACAAGGCAAGAGGAGGGGCATGATTGAGGCAGTGATACAGAAAGCAGACAGTAGAGGTCGTGGCAAGTGTGCCGTTACTTGCTACCTGTGGTTGATGGGAGAGTCACACCACATTTAGGAGGAGAGAATCCATTTGCCACTTCTGACAATGCCACAAGAATCACATATTTCATCCAGAGGTTGAATTTGGCCCATGCTGAGCTTTAAAATACAGAGCTGTCTTGGAACAATGGCTCAGTACATTCATTTGGTGTCCAACAAAGCCTGCCTCTGTTGCCTTCCCTCTCTCTGTGTGCCCTTCAAGATCTTCATTGTGCTTTGGGGAGAGAAAGAGAAAATGTCATATCAGGGTAGCTCACCCCATGTGTCCTGGACTCAGGAAAAGAGTATCTTATCACCTTACTCTTTTGTTATTATAAAAAATAAAGTTGAACGTCTTCAAATAAAATAAAGAAGTATAGAAAAAATTTTAAATTAACCTGTTATGATTCTACCTAGAGAACCATTGTCAACATCTTGGTATATGTACTTCCAGATACTTTCCTATGAATATATACATTGTAGATTTTTTAATATTAAAAGGCTATCATGCTGCTTTGTATACAGGCTTTCTTTACTGATATGTAATATAATACACAGACAAATATACAAATCCTAAGCCATCAACTCATTGAATTTTTATTCGTTGTTTTTAATACCTGCATTGTGTTCCATTGTTAGGCTATGTCACAACATATTTAATTAAGCCCCTATTGATGAATATTAATTTACTCTATTTGCCAGTTCATTCCAGTCCAACATTTATTGAGTGTCTACTTACGGGCCAGGCACTCTTGTATTCATCAAGATCACCACATTATCTGTATCAGTTATTTATTGCCACAATAAAACTGCATAACAAATCACTCCAAAATGTAGCACCTTAAAACTACAACTACTTATTATTTCTCAAGAGTCAATGGGTCAGCTGAGCAGTTCTGCCGATAGGGGTCAAGGTCAACACATTTCAACTAGACTACTTGTAAAAAAGAATGAGTGTCTGGGTAGGTGTGTTCTTCTAAAAATAAAACAAGGAATGAGGAAATTGCAGGTAGGATAAGAGGGGTGGTTGGCAACCAAACCCCACAAAAGGCAGACAAATTTTAAGGAAACATAATGCCAGACTCCTATGTCATCATCCAAGTAGATGCAGTGAAGTATAACCTGGGGCGTAGTAGGGTAGGAGTGGGGAGAGCAGAGGAGAAGGAAGGGAGATTGCTTTTCATCACTTTTGGATTCCCTAATAACAGACATGACTGCCAGTATTAAAATTTAACAAAGGATATCTGATCATTAATTTTCCTGTATAAGTCACTGGTGATCTTCAACATCTCTCCCTCCCTTCCTCCCTTCCTTCCTCCCACCCTCCCTTCCTTCCTTCTTTCCTCTTTTGCTTTCAACTTCCTTTTCTCGTTTCCTTTTGCTTTCTTTCTCTTCTCCCTTTTTTCTGTCACTCTGGGCGTATGTAGTAGTGTAAAAAGGTTGACAGAGAAATCAAATATAACAGGAGCAGGGCCCTGAGAAAAGCACCTGGCATCCTGTAGGCAAACCATTGTTTCTAAAAGAAGGGACTGAGAGATTGAGGAGCTCAGGACATTGCCAAATGAACAAGGCAAGCACATTTATTCAGTACCAAACAAACGGAAAACGGCCTTTCCAAATAACTGACCTATAAAACAGCCTTTTCACAAGAGTACCGTAATTACTGGCCAACAGCAACAATGAAAAACAACTCCCAAACAAAGAAATATTTCTGGATTAAAAGCCATGAGATCTGGATTCTAACAAGCTGTGCTCCTCAAACTACAAGTACAAAATCTGGCTCTAAACTAACAAGCTATGAGCCTCAAACTGATGACTGGCATGTTTGGGTCTCCATCTCCTTCTTGGGGGTTGGGGTCTTAGAGACCCTTTTCCACGCCCTGATTCTCTTACTAGTGTGTATGCTTTCCTTTTGACTTCTCATGCTGACCGTCTGAGCAGGAGTGAGAAGCAATTTCAAAGGAAAACATCGTTTATCATCTGCTGAAAGAAACCAAAAAGAACACAGGAAAACAAAAAGACAAGGAAAGGGAATGAAAATGTAATTCATTTTATTAAAAAGAAGAATTATTCTTCTGGGACACTGGATAGAAACCTTAATGAGTTACCTAGCTATCATAAATCCTCTAACAGAGAAGAGAAGAGAAAGAAACAAAGACGGAAGAGGGCAGGATAAAAGAAAGAAAAAAGGAAGGGAAAAATGAAGGAAGGAAGTTATCTATTCATTTCTACAGAGACTCTGCTGAGCAGTAGACAAGAAGACTTGGGAAAAATTTAACTGAAACTTTTCCAAAAATCTTTTCAGAGGGATTTTTTCCCTCTGAAAAGCATCATTAGAGGCTGTTCAATACCCAAGGCAAGCCTCTTTCATATTACTTACTGTACATGAAACACTCATGCAATTGAGGCTAGCCAGAGGCCATTTAGAAATTCAATAATTATTCAACCCAAGGGGCTTTCCAAATGGTGAAGTAGCTTCTTAAGAGGAAATTAATATTGAGCAGTATAGCAAACCTAATTGGAATCTTGAGAAAATAGTTCTGTGTCGTTAGAACAGCTAGAGGCTAAAGAAGATCAGGTTGGATGATACCTTCATTTTTGTCTCTTTCCTTAATTATGATGTAAAGGGAAAAATCTTGTTTATTTTCTATGCCAGGAGGGTAGAGGGTGATTTGGAGAGGTTCCAAGTTTATCAAAATCTACCTTCAGTCTGGCAGTAGAAAAGTTTACTTCCTTCATTTCTTTCCTATAGACATTCAAAGAGAGCTAAGGAGATCCAAAAACCTTTTTTTCTATATTTGCAATGCAAGGCAGTTGGGAATTAATGACTGATTTGTTGGTGAGGGCAGTGGGCATTGATCACAAAAGCAGTAAAGCTGTGTTTCTCAAAGAGAGAAAGTCTCTTTGAGATCTTCATTATTTTACTATTTAGAAGAGAAAGGGGCGTTATATCACGTTGGAAGCATCCATGAGTCACTAGTCTCTTCTCTATCTTTCTATGCCTTTCTGTATTAATTACTTTGAAAGCACAACATTCCAAACCCATTGAGCACACAGTGGTCTGATTTCTCCACTTGTGAAAGGTGCTAAAGTCTCACTGTAGGATTAATTTGGGGGTCCAGGCTATGGGCTTGTAGATATGACTACCTTAGACTTTGGTTCTCCTGGCAACTAACCCTTTTTGGATCGTATCTAAGTTGACCTGTTTCACAGTGAGAGAACTCCTCTCCATTACTCAGAATACTGAGGCAGATCACAAGTGTACCACACCTGGCTAATGTTAAGCCAGACAGAAACATCAGGCTCATCTCTTGAGAAGAAGGGTCGCTTATTAAGGATACAAACTATTTTTTTTTTTTTTTTTGAGACAGGGTCTCATTGCCCAGGTTAGAGTGCAGTGGTGCAATCATAGCTCACTGCAGCCTCAACCACATGGGTATTTTTAAATAAGAAAAAAATACCATCTGATAGATATGAAGGAGCATTGGGTCACTATAAACAAAACAGATTCTAAGAGCAGGAAGAAAGAGTACAGTCTCTTTTCAATAATTTTTTTTTAAACTTGGGAAAGAACACTCACTCTATTCCTATAGACCAGAAAGCAGATAATTGTCCATTATGATTCCACATGACACTATCTTGTTCAGCTGTCACTGAAACAACTTTGAACACTGTCATATGTTCTTCCCAGCTCCTGAACTCTGACCTTTTTATGCCTTAGTTCCACTTTCACAAAAAGGGATTGATGTAATGTGCATTTCAGAGGAAACGACTATAGACATTTAGTGTCATTATAAATGTTGAGAAGTATGCTGGCAGAAATTATGCCTTAAGATCATATATGGATTCTTGTATGGTTTGAAATTGCTTAAAAGATATATATGATCTCTAAAATGTGTGTGTATATATATATGATGTCTTCTTATATATCTATATGTGATATATTTATATATATATAAATCTGTGTATATCACATATATAAATTTGCTGTTATTTGAATTGCCATTACCTCAGTGCTTAGGGGAAGCCATGCACGTTTGTTTCTTTTCAGTACCCAGAGTTAATTAACATAAGTTATCACAGAAGCTCCCATAAGCATTGAGACAATTTCTCTATACCTGTGACTATTTAAGGTTTTGAAAACAAAACAGAAGCAGGTAAGGAGGAAGTACGCTTTACTATTGAAGATTTATTAGGTACACATTTAGATTTGTGAACTCACATTGCTTAGGATGAAAGGGACTCTTGAGGATGTCTGCTGTTTGTTAGTGAACTGCCTGTAACAATTACAATTAGCACACACATGAGCACAATGAACTGGGTAGTCAGACTCAGCCAAAATGAATAGAAATAGCCTCTTACCAAATTTACTTTGAGTAGCCCTTGGACTCTGAGCACTGCTGCCCAGAGCAATATGACTGTAGGTCCAAGTTTGTCAATGACTATGCAAATGTGCTTTCTTCGCTTTTACTCTATTGTCATCTGTCTATTACAATGTTGCTATGGTGACACCTTTCCAATATCCCTGTGCTTCTTTGGTATCCTCTAAGGGGAAGCTGTAATGAAGTGGCTTGGCAAAAGAATCCTCTTGGAATTTTTTTTTTTTCATATGCTACTGAAAACCAGCATGATTTTCCTCTTATGGGAAATGTATAAAGTATGAGTTGGAAATGATGGAAATTAATCTGTACTGACTTGGGCAAGGAATGTGAATGTTATTCATTCTGTTCCAAACTACCTGAAAATATTCTCTTTCTGTTCCTACTTTCCAGGAGATAACATCTTAAGGGACACTGAAGCTTGTGCGTGTGTGAGTAGAACACGTGCTGGGGGCTCTTGAGCTCATGAGGGAGGGGCTACATGTCGGTGGGGTGATAACTGTATGCTGGAAACAATGATAGGTGGTGACCCTGGAGCACTTACCATGTGACAGGTGTTATGCTAAGCATGTTGTATGCATTCCTTCATTGAATGACAGCTACCTATATTATCCTCATTTTATAAGATGAGGTAACAGAGCTTCAGAAAGGTTAGACTCAGCTGCTATGGGTCTGTCTGACTCTGGTGTTCTTCCTCTTAAAAACTGGGGCACTTTGGAAATGAGATTCCTCGGTGATGAACAGAAATATTGCTTAGCGGCTGTATTTTTGTATCTGGCAGTTTTCCCATATTTGAGTCTTATATTCACAATCGGTATCTTTACATTACACAAAAGTGACACAGAATTAGAGTCATTTAATCCAGGGTTGATATCATTAAGTCATGACTATTTATTAAATGTTTCTTACAATATCTGAGATGATATTGCAAAAGATGTAAGTGATTTTAGAAGTTCTCACTTCGTAGTTAGTTGCAGAAACCTCTTTTGGAGGAGGGATGTTTTCTCTATATATCCTAATTTCTACTTAATATATTTCCACACCTCTTTGAAGTGTGTAGTAAGAATGGTAAAATGCAGTACTTCGTCATTTGGTACAGTTCAATCAATATGCATTAAGATGTGATCATATGGGTAATAGAAAAATGTGAAAGATCCAATTCTTTTTCTCCAGAAGGCAGGAAGCTCATATTTGATTTCTGTTACTATAAACTATAAAAACGTTTCAAATGTAGTTTACCCGTAACCATCACCCTGCAAGGGTGATATTGCTCCCCGCCAATTTACGGAGGAGAATACTGAGGCTTTAAGGTTGTAGATAGACCAAGACCACACAAGTAGAGAGTGGCGGGCTGTGGGTTGAGCTTTAAAATCCAGGTTCATCCATGACTCCCAGTGTGTTCTAGTAAATCCACTAGAATCTGAGTATTTTCCAATGATTTATGCTCCGCTCTGTGTCAGGCAGTTCATGGTATTTTTCAACAATCAGAAAATCCTGGGGAAGGCAAACTGTTTCCCCCTCTCTAGGTGCCTTGGAAGTGGCCGTTGTGGACCCAGAGATCATCCTTTCTGATCTGACACCTTCTTCACTGCCCTGGCCCAGTGTCTTTTCTGCAAGGCTGGAAGCCCCCTTAGACTGGTCATGTCCCATCTCTTTCCGGAGGGAAGATGATCCCAAAGACGACTTTTCTCTCCACGGTGCTGCCATACCGCAGGCGGCCGCCAGGGGTCCCCGCTCGGCGTCCCCGCGAGACAGTCGAGCCCCGGCCGGCTGCGCGGCGCGCTGGGTGCATGAGGGGGCTGCTCCGGAGCGACGGCGGCTGCAGCTGGAGCCAGGCGCTCGCCCGTCCGCCGGTTGGCTCGCCGGGACCTCGCGCACCGGCGGCAGAGTCCCTTGCGTGGATTGGCAAGCGACGCCCCACCTGCCCCGAGCTCACCATTTTCTTTCGCGCTGGCTGCAGCTGACCCGGCGAAGGGAGCCGACCGGGCCCTGGGCTGGAGGTAAAACCCCACGGTGAGTAAGAACCCGCTCCAAGCTAGGGGAGGCGGCGCAGCCCGGTGGCTGCTCGCTCCCGATCTCGCCCGGGCGGGCGGCGAGGTTTGGGGCGCACCTGGGCGCGGGTGCAAGAAGGTGCGGGAGGCGGCGGACCGGTCTTCTGCCCGCCGGCCACGGGCTTCCGGGGCTGGAGTCCTCTTCAGACCCCTGCCGGCGCCTGGGTTTCTGGCCGGCTCCTCGTGTGCACTTCCCGGCAGGAACAAGGGTCGCCCACTTTCCACCCCGGGATCTTGATTTGTCCTTGATTTGAAAAGATATAAATCAATAAGATCGTCCTTCTTTCGGGGTGCAAGACTCCGAGCCCATCCCCAGCCGCGGACGCCTGCAGGGTGCGTGTTGGGCTGTGGGTGGCGGGAAGACAAACTTTTACAAAAGTGCGCCTGGGCTGGGGGACAACGCTTGGGCGTCCTGATCCTGAGGGAGGAGTCTCGGCTTGGGGCAGCGTAGGGGAAGTCCGCACCGTCAGCCAGGTCGCCCCCGGGGCTGACGATGCCTCACGGAGGTGGGGAGCGTGTAAAGGCCGTACAAATCGCGCTTAACTTTGGGGCCAACAACTGTCAAACATCTGGAATCCCAGCCCCTCCCTTTCCCTGAACTGGGGAAGAAGGTGAAAACCCTTCAACTTTTCTTTGATTGCCCCTTCCCACCTTCAGACCCCTGCTGGGAGGGTAAAGCGCCGACCCCTGGTGCCTGGCAAGTACCAGAGACTCTAAATCTCTCGGGATCCCCCCCTCGCGCTCTTTCCTGACCCTCTCCCCTAACCCTCCCCACAGAGATCTCTCTACGCAGCCGACTGAGATCGTGGCGAATGGCCTTTTGTTTCTCCGCGTTTCCCCTATTGTTTGCCTTTCCAACATCTGGCGGGGCTTGGGGAGAGAAGGAAGCCCCTCTGGTCCCCCTCCCCGGCCCCCACGCCAGCTCCGGCAGGGGATCCCAGCTGGGAAAGTGGAGGAGCCCGACCCCAGCGAGGCCGCCCCACCCCGCCCTTGTGGTTAGAGGGCGGAGGGAAAGTTGTTCCTTCCCCGCCTCCGCTGCTGCCTGTGGCCCAGGGCGCATTTCTCAGATCTCAGCCCAGGCGCGCCGCAAAGGCGCAAATCCGAGAAGGTGCTGCTTTCGAGACAGTGGAAGCGCGTTCCGCCCCAATCCAGAGCGTCCAGTGGTTGGTTCCAGAGGATTTCAATCTCTAGCCAAAGGCGTTGGGGCTGGGCCGCTGCTAGGGCAGTGGGAGGGGATCGGGGCACCTTTGGTAGGCGGAAAGCTGAGATTCTGGGGTCCACAAGTTTCCAAGGGCGGGAGGGCAGGCTAGTCGCCAAAAAGAGAACGAAGATGCAAATAACGAGGAAGCCTTATGACGTTGCCTGGAAATAGTAGTGTGGTGGTTCACTCCGGAATGAACGTGGAGTTCTGGCTTTGAGTACCGCTCCAAGTTTAAATCCCAAGTCCCCTTTCTTCATTGTAGAAAAAGAGGACTCAGACGACGCAACACAGATACGGCTAGAGCACAGTTCCTGCTTCCACGTCCCAGAGAACAAGTGGCTTAGGATGGTCCCGAGTTCCCCTGTGGGTGCGCTTGTTGGGTTGCAGGCGGCCCTGTTTCCCTGCACAAGTCAGATGCTTACACATTGTGTTCATTCTTAGTGTGGATTATTGATTAAAGAACTGGGGCAAAAGCAAAGTAGCTACTCTGAGAAGTCAGGGTCCCCAGATGGTGCCCAGCGAGTTGTCTTGCCTCTGAGGGGAGGCTGACTGAGACTGTGCACCTGTTAGAACCTATGCTACCCCATAGCCTTGCAGTTGACTTGCTGTTGCCAGCTTTTCCTGTGGGATCCCCAATGAGTCCCTCTTCCAAGGAAGCTCAATTACACTTTTGATTCCTCCTCAACCCAGGGGAAGAAAGAGGCTTCTGTAGGAACATTATGATCTATGTACCCACTCAGACATTGTCAGTGGATACCAGAAGCTTGGCTCTGCACAGCTCTGAGAGTTTTCCCTTTGCGCACTCAACAGAACTTTTGAGTTTCCATTTAACATAAAAGAAGTGAGACTGCTAAGCCAGGAATGCGACACATAGAGCACTTTCTCTAGTGATTTCTGGGTATTATATCTCTTTACCTTCCCAACGGTGGAACCAGGAAAAGAAAAAAAAAGCAACATCTTTGAAGTACTGCAAGGCACTTTACAAACATTTCATTATGAAAATGATCCCCAAGGAAGGATTCCTTTGAAATTTAGCAGCAGCAACCCAGAAGCAACAAAAAAGACCAAAGTTACTCAAGAAGTACCCAAAGGCATCATTAACAAAATAAAAGAGCATTTCTTGTCTTGGCCTACCCCGCTGAGGAAAACAGGGTAATTATAGTGGAAGTTAAGCTTGCATGCCTGCAGGTCGACTCTAGAGGATCCCCGGGTACCGAGCTCGAATTCGCCCTATAGTGAGTCGTATTACAATTCACTGGCCGTCGTTTTACAACGTCGTGACTGGGAAAACCCTGGCGTTACCCAACTTAATCGCCTTGCAGCACATCCCCCTTTCGCCAGCTGGCGTAATAGCGAAGAGGCCCGCACCGATCGCCCTTCCCAACAGTTGCGCAGCTGAATGGCGAATGGCGCCTGATGCGGTATTTTCTCCTTACGCATCTGTGTGGTATTTCACACCGCATATGGTGCACTCTCAGTACAATCTGCTCTGATGCCGCATAGTTAAGCCAGCCCCGACACCCGCCAACACCCGCTGACGCGAACCCCTTGCGGCCGCATCGAATATAACTTCGTATAATGTATGCTATACGAAGTTATTAGCGATGAGCTCGGACTTCCATTGTTCATTCCACGGACAAAAACAGAGAAAGGAAACGACAGAGGCCAAAAAGCTCGCTTTCAGCACCTGTCGTTTCCTTTCTTTTCAGAGGGTATTTTAAATAAAAACATTAAGTTATGACGAAGAAGAACGGAAACGCCTTAAACCGGAAAATTTTCATAAATAGCGAAAACCCGCGAGGTCGCCGCCCCGTAACCTGTCGGATCACCGGAAAGGACCCGTAAAGTGATAATGATTATCATCTACATATCACAACGTGCGTGGAGGCCATCAAACCACGTCAAATAATCAATTATGACGCAGGTATCGTATTAATTGATCTGCATCAACTTAACGTAAAAACAACTTCAGACAATACAAATCAGCGACACTGAATACGGGGCAACCTCATGTCCGAGCTCGCGAGCTCGTCGACAGCGACACACTTGCATCGGATGCAGCCCGGTTAACGTGCCGGCACGGCCTGGGTAACCAGGTATTTTGTCCACATAACCGTGCGCAAAATGTTGTGGATAAGCAGGACACAGCAGCAATCCACAGCAGGCATACAACCGCACACCGAGGTTACTCCGTTCTACAGGTTACGACGACATGTCAATACTTGCCCTTGACAGGCATTGATGGAATCGTAGTCTCACGCTGATAGTCTGATCGACAATACAAGTGGGACCGTGGTCCCAGACCGATAATCAGACCGACAACACGAGTGGGATCGTGGTCCCAGACTAATAATCAGACCGACGATACGAGTGGGACCGTGGTCCCAGACTAATAATCAGACCGACGATACGAGTGGGACCGTGGTTCCAGACTAATAATCAGACCGACGATACGAGTGGGACCGTGGTCCCAGACTAATAATCAGACCGACGATACGAGTGGGACCATGGTCCCAGACTAATAATCAGACCGACGATACGAGTGGGACCGTGGTCCCAGTCTGATTATCAGACCGACGATACGAGTGGGACCGTGGTCCCAGACTAATAATCAGACCGACGATACGAGTGGGACCGTGGTCCCAGACTAATAATCAGACCGACGATACGAGTGGGACCGTGGTCCCAGTCTGATTATCAGACCGACGATACAAGTGGAACAGTGGGCCCAGAGAGAATATTCAGGCCAGTTATGCTTTCTGGCCTGTAACAAAGGACATTAAGTAAAGACAGATAAACGTAGACTAAAACGAGGTCGCATCAGGGTGCTGGCTTTTCAAGTTCCTTAAGAATGGCCTCAATTTTCTCTATACACTCAGTTGGAACACGAGACCTGTCCAGGTTAAGCACCATTTTATCGCCCTTATACAATACTGTCGCTCCAGGAGCAAACTGATGTCGTGAGCTTAAACTAGTTCTTGATGCAGATGACGTTTTAAGCACAGAAGTTAAAAGAGTGATAACTTCTTCAGCTTCAAATATCACCCCAGCTTTTTTCTGCTCATGAAGGTTAGATGCCTGCTGCTTAAGTAATTCCTCTTTATCTGTAAAGGCTTTTTGAAGTGCATCACCTGACCGGGCAGATAGTTCACCGGGGTGAGAAAAAAGAGCAACAACTGATTTAGGCAATTTGGCGGTGTTGATACAGCGGGTAATAATCTTACGTGAAATATTTTCCGCATCAGCCAGCGCAGAAATATTTCCAGCAAATTCATTCTGCAATCGGCTTGCATAACGCTGACCACGTTCATAAGCACTTGTTGGGCGATAATCGTTACCCAATCTGGATAATGCAGCCATCTGCTCATCATCCAGCTCGCCAACCAGAACACGATAATCACTTTCGGTAAGTGCAGCAGCTTTACGACGGCGACTCCCATCGGCAATTTCTATGACACCAGATACTCTTCGACCGAACGCCGGTGTCTGTTGACCAGTCAGTAGAAAAGAAGGGATGAGATCATCCAGTGCGTCCTCAGTAAGCAGCTCCTGGTCACGTTCATTACCTGACCATACCCGAGAGGTCTTCTCAACACTATCACCCCGGAGCACTTCAAGAGTAAACTTCACATCCCGACCACATACAGGCAAAGTAATGGCATTACCGCGAGCCATTACTCCTACGCGCGCAATTAACGAATCCACCATCGGGGCAGCTGGTGTCGATAACGAAGTATCTTCAACCGGTTGAGTATTGAGCGTATGTTTTGGAATAACAGGCGCACGCTTCATTATCTAATCTCCCAGCGTGGTTTAATCAGACGATCGAAAATTTCATTGCAGACAGGTTCCCAAATAGAAAGAGCATTTCTCCAGGCACCAGTTGAAGAGCGTTGATCAATGGCCTGTTCAAAAACAGTTCTCATCCGGATCTGACCTTTACCAACTTCATCCGTTTCACGTACAACATTTTTTAGAACCATGCTTCCCCAGGCATCCCGAATTTGCTCCTCCATCCACGGGGACTGAGAGCCATTGCTATTGCTGTATTTGGTAAGCAAAATACGTACATCAGGCTCGAACCCTTTAAGATCAACGTTCTTGAGCAGATCACGAAGCATATCGAAAAACTGCAGTGCGGAGGTGTAGTCAAACAACTCAGCAGGCGTGGGAACAATCAGCACATCAGCAGCACATACGACATTAATCGTGCCGATACCCAGGTTAGGCGCGCTGTCAATAACTATGACATCATAGTCATGAGCAACAGTTTCAATGGCCAGTCGGAGCATCAGGTGTGGATCGGTGGGCAGTTTACCTTCATCAAATTTGCCCATTAACTCAGTTTCAATACGGTGCAGAGCCAGACAGGAAGGAATAATGTCAAGCCCCGGCCAGCAAGTGGGCTTTATTGCATAAGTGACATCGTCCTTTTCCCCAAGATAGAAAGGCAGGAGAGTGTCTTCTGCATGAATATGAAGATCTGGTACCCATCCGTGATACATTGAGGCTGTTCCCTGGGGGTCGTTACCTTCCACGAGCAAAACACGTAGCCCCTTCAGAGCCAGATCCTGAGCAAGATGAACAGAAACTGAGGTTTTGTAAACGCCACCTTTATGGGCAGCAACCCCGATCACCGGTGGAAATACGTCTTCAGCACGTCGCAATCGCGTACCAAACACATCACGCATATGATTAATTTGTTCAATTGTATAACCAACACGTTGCTCAACCCGTCCTCGAATTTCCATATCCGGGTGCGGTAGTCGCCCTGCTTTCTCGGCATCTCTGATAGCCTGAGAAGAAACCCCAACTAAATCCGCTGCTTCACCTATTCTCCAGCGCCGGGTTATTTTCCTCGCTTCCGGGCTGTCATCATTAAACTGTGCAATGGCGATAGCCTTCGTCATTTCATGACCAGCGTTTATGCACTGGTTAAGTGTTTCCATGAGTTTCATTCTGAACATCCTTTAATCATTGCTTTGCGTTTTTTTATTAAATCTTGCAATTTACTGCAAAGCAACAACAAAATCGCAAAGTCATCAAAAAACCGCAAAGTTGTTTAAAATAAGAGCAACACTACAAAAGGAGATAAGAAGAGCACATACCTCAGTCACTTATTATCACTAGCGCTCGCCGCAGCCGTGTAACCGAGCATAGCGAGCGAACTGGCGAGGAAGCAAAGAAGAACTGTTCTGTCAGATAGCTCTTACGCTCAGCGCAAGAAGAAATATCCACCGTGGGAAAAACTCCAGGTAGAGGTACACACGCGGATAGCCAATTCAGAGTAATAAACTGTGATAATCAACCCTCATCAATGATGACGAACTAACCCCCGATATCAGGTCACATGACGAAGGGAAAGAGAAGGAAATCAACTGTGACAAACTGCCCTCAAATTTGGCTTCCTTAAAAATTACAGTTCAAAAAGTATGAGAAAATCCATGCAGGCTGAAGGAAACAGCAAAACTGTGACAAATTACCCTCAGTAGGTCAGAACAAATGTGACGAACCACCCTCAAATCTGTGACAGATAACCCTCAGACTATCCTGTCGTCATGGAAGTGATATCGCGGAAGGAAAATACGATATGAGTCGTCTGGCGGCCTTTCTTTTTCTCAATGTATGAGAGGCGCATTGGAGTTCTGCTGTTGATCTCATTAACACAGACCTGCAGGAAGCGGCGGCGGAAGTCAGGCATACGCTGGTAACTTTGAGGCAGCTGGTAACGCTCTATGATCCAGTCGATTTTCAGAGAGACGATGCCTGAGCCATCCGGCTTACGATACTGACACAGGGATTCGTATAAACGCATGGCATACGGATTGGTGATTTCTTTTGTTTCACTAAGCCGAAACTGCGTAAACCGGTTCTGTAACCCGATAAAGAAGGGAATGAGATATGGGTTGATATGTACACTGTAAAGCCCTCTGGATGGACTGTGCGCACGTTTGATAAACCAAGGAAAAGATTCATAGCCTTTTTCATCGCCGGCATCCTCTTCAGGGCGATAAAAAACCACTTCCTTCCCCGCGAAACTCTTCAATGCCTGCCGTATATCCTTACTGGCTTCCGCAGAGGTCAATCCGAATATTTCAGCATATTTAGCAACATGGATCTCGCAGATACCGTCATGTTCCTGTAGGGTGCCATCAGATTTTCTGATCTGGTCAACGAACAGATACAGCATACGTTTTTGATCCCGGGAGAGACTATATGCCGCCTCAGTGAGGTCGTTTGACTGGACGATTCGCGGGCTATTTTTACGTTTCTTGTGATTGATAACCGCTGTTTCCGCCATGACAGATCCATGTGAAGTGTGACAAGTTTTTAGATTGTCACACTAAATAAAAAAGAGTCAATAAGCAGGGATAACTTTGTGAAAAAACAGCTTCTTCTGAGGGCAATTTGTCACAGGGTTAAGGGCAATTTGTCACAGACAGGACTGTCATTTGAGGGTGATTTGTCACACTGAAAGGGCAATTTGTCACAACACCTTCTCTAGAACCAGCATGGATAAAGGCCTACAAGGCGCTCTAAAAAAGAAGATCTAAAAACTATAAAAAAAATAATTATAAAAATATCCCCGTGGATAAGTGGATAACCCCAAGGGAAGTTTTTTCAGGCATCGTGTGTAAGCAGAATATATAAGTGCTGTTCCCTGGTGCTTCCTCGCTCACTCGACCGGGAGGGTTCGAGAAGGGGGGGCACCCCCCTTCGGCGTGCGCGGTCACGCGCACAGGGCGCAGCCCTGGTTAAAAACAAGGTTTATAAATATTGGTTTAAAAGCAGGTTAAAAGACAGGTTAGCGGTGGCCGAAAAACGGGCGGAAACCCTTGCAAATGCTGGATTTTCTGCCTGTGGACAGCCCCTCAAATGTCAATAGGTGCGCCCCTCATCTGTCAGCACTCTGCCCCTCAAGTGTCAAGGATCGCGCCCCTCATCTGTCAGTAGTCGCGCCCCTCAAGTGTCAATACCGCAGGGCACTTATCCCCAGGCTTGTCCACATCATCTGTGGGAAACTCGCGTAAAATCAGGCGTTTTCGCCGATTTGCGAGGCTGGCCAGCTCCACGTCGCCGGCCGAAATCGAGCCTGCCCCTCATCTGTCAACGCCGCGCCGGGTGAGTCGGCCCCTCAAGTGTCAACGTCCGCCCCTCATCTGTCAGTGAGGGCCAAGTTTTCCGCGAGGTATCCACAACGCCGGCGGCCGGCCGCGGTGTCTCGCACACGGCTTCGACGGCGTTTCTGGCGCGTTTGCAGGGCCATAGACGGCCGCCAGCCCAGCGGCGAGGGCAACCAGCTCGAGGGCTTCGCCCTGTCGCTCGACTGCGGCGAGCACTACTGGCTGTAAAAGGACAGACCACATCATGGTTCTGTGTTCATTAGGTTGTTCTGTCCATTGCTGACATAATCCGCTCCACTTCAACGTAACACCGCACGAAGATTTCTATTGTTCCTGAAGGCATATTCAAATCGTTTTCGTTACCGCTTGCAGGCATCATGACAGAACACTACTTCCTATAAACGCTACACAGGCTCCTGAGATTAATAATGCGGATCTCTACGATAATGGGAGATTTTCCCGACTGTTTCGTTCGCTTCTCAGTGGATAACAGCCAGCTTCTCTGTTTAACAGACAAAAACAGCATATCCACTCAGTTCCACATTTCCATATAAAGGCCAAGGCATTTATTCTCAGGATAATTGTTTCAGCATCGCAACCGCATCAGACTCCGGCATCGCAAACTGCACCCGGTGCCGGGCAGCCACATCCAGCGCAAAAACCTTCGTGTAGACTTCCGTTGAACTGATGGACTTATGTCCCATCAGGCTTTGCAGAACTTTCAGCGGTATACCGGCATACAGCATGTGCATCGCATAGGAATGGCGGAACGTATGTGGTGTGACCGGAACAGAGAACGTCACACCGTCAGCAGCAGCGGCGGCAACCGCCTCCCCAATCCAGGTCCTGACCGTTCTGTCCGTCACTTCCCAGATCCGCGCTTTCTCTGTCCTTCCTGTGCGACGGTTACGCCGCTCCATGAGCTTATCGCGAATAAATACCTGTGACGGAAGATCACTTCGCAGAATAAATAAATCCTGGTGTCCCTGTTGATACCGGGAAGCCCTGGGCCAACTTTTGGCGAAAATGAGACGTTGATCGGCACGTAAGAGGTTCCAACTTTCACCATAATGAAATAAGATCACTACCGGGCGTATTTTTTGAGTTATCGAGATTTTCAGGAGCTAAGGAAGCTAAAATGGAGAAAAAAATCACTGGATATACCACCGTTGATATATCCCAATGGCATCGTAAAGAACATTTTGAGGCATTTCAGTCAGTTGCTCAATGTACCTATAACCAGACCGTTCAGCTGGATATTACGGCCTTTTTAAAGACCGTAAAGAAAAATAAGCACAAGTTTTATCCGGCCTTTATTCACATTCTTGCCCGCCTGATGAATGCTCATCCGGAATTTCGTATGGCAATGAAAGACGGTGAGCTGGTGATATGGGATAGTGTTCACCCTTGTTACACCGTTTTCCATGAGCAAACTGAAACGTTTTCATCGCTCTGGAGTGAATACCACGACGATTTCCGGCAGTTTCTACACATATATTCGCAAGATGTGGCGTGTTACGGTGAAAACCTGGCCTATTTCCCTAAAGGGTTTATTGAGAATATGTTTTTCGTCTCAGCCAATCCCTGGGTGAGTTTCACCAGTTTTGATTTAAACGTGGCCAATATGGACAACTTCTTCGCCCCCGTTTTCACCATGGGCAAATATTATACGCAAGGCGACAAGGTGCTGATGCCGCTGGCGATTCAGGTTCATCATGCCGTTTGTGATGGCTTCCATGTCGGCAGAATGCTTAATGAATTACAACAGTACTGCGATGAGTGGCAGGGCGGGGCGTAATTTTTTTAAGGCAGTTATTGGTGCCCTTAAACGCCTGGTTGCTACGCCTGAATAAGTGATAATAAGCGGATGAATGGCAGAAATTCGATGATAAGCTGTCAAACATGAGAATTGGTCGACGGCCCGGGCGGCCGCAAGGGGTTCGCGTTGGCCGATTCATTAATGCAGCTGGCACGACAGGTTTCCCGACTGGAAAGCGGGCAGTGAGCGCAACGCAATTAATGTGAGTTAGCTCACTCATTAGGCACCCCAGGCTTTACACTTTATGCTTCCGGCTCGTATGTTGTGTGGAATTGTGAGCGGATAACAATTTCACACAGGAAACAGCTATGACCATGATTACGCCAAGCTATTTAGGTGAGACTATAGAATACTC
